# Improved accuracy and less fault prediction errors via modified sequential minimal optimization algorithm

**DOI:** 10.1371/journal.pone.0284209

**Published:** 2023-04-13

**Authors:** Muhammad Asim Shahid, Muhammad Mansoor Alam, Mazliham Mohd Su’ud

**Affiliations:** 1 Malaysian Institute of Information Technology, Universiti Kuala Lumpur, Kuala Lumpur, Malaysia; 2 Faculty of Computing, Riphah International University, Sector I-14, Islamabad, Pakistan; 3 School of Computer Science, University of Technology Sydney, Ultimo, NSW, Australia; 4 Persiaran Multimedia, Multimedia University, Cyberjaya, Malaysia; Vellore Institute of Technology: VIT University, INDIA

## Abstract

The benefits and opportunities offered by cloud computing are among the fastest-growing technologies in the computer industry. Additionally, it addresses the difficulties and issues that make more users more likely to accept and use the technology. The proposed research comprised of machine learning (ML) algorithms is Naïve Bayes (NB), Library Support Vector Machine (LibSVM), Multinomial Logistic Regression (MLR), Sequential Minimal Optimization (SMO), K Nearest Neighbor (KNN), and Random Forest (RF) to compare the classifier gives better results in accuracy and less fault prediction. In this research, the secondary data results (CPU-Mem Mono) give the highest percentage of accuracy and less fault prediction on the NB classifier in terms of 80/20 (77.01%), 70/30 (76.05%), and 5 folds cross-validation (74.88%), and (CPU-Mem Multi) in terms of 80/20 (89.72%), 70/30 (90.28%), and 5 folds cross-validation (92.83%). Furthermore, on (HDD Mono) the SMO classifier gives the highest percentage of accuracy and less fault prediction fault in terms of 80/20 (87.72%), 70/30 (89.41%), and 5 folds cross-validation (88.38%), and (HDD-Multi) in terms of 80/20 (93.64%), 70/30 (90.91%), and 5 folds cross-validation (88.20%). Whereas, primary data results found RF classifier gives the highest percentage of accuracy and less fault prediction in terms of 80/20 (97.14%), 70/30 (96.19%), and 5 folds cross-validation (95.85%) in the primary data results, but the algorithm complexity (0.17 seconds) is not good. In terms of 80/20 (95.71%), 70/30 (95.71%), and 5 folds cross-validation (95.71%), SMO has the second highest accuracy and less fault prediction, but the algorithm complexity is good (0.3 seconds). The difference in accuracy and less fault prediction between RF and SMO is only (.13%), and the difference in time complexity is (14 seconds). We have decided that we will modify SMO. Finally, the Modified Sequential Minimal Optimization (MSMO) Algorithm method has been proposed to get the highest accuracy & less fault prediction errors in terms of 80/20 (96.42%), 70/30 (96.42%), & 5 fold cross validation (96.50%).

## Introduction

Cloud computing (CC) first appeared in information technology and has since become a popular business model for providing IT infrastructure, components, and applications [1]. Cloud computing is defined by five distinct characteristics: on-demand self-service, extensive network access, resource pooling, rapid elasticity, and measured service. There are four deployment models available as well: private clouds, community clouds, public clouds, and hybrid clouds. Furthermore, it has three service models: SaaS (software as a service), PaaS (platform as a service), and IaaS (infrastructure as a service). Through dynamic service provisioning, CC aims to supply computation and resources over the internet. There are numerous challenges and issues associated with CC deployment. These are data protection, data retrieval, and availability issues, administrative capabilities, regulatory and compliance constraints, security, load adaptability, execution monitoring, LB, FT, CC governance, interoperability, and portability. The cloud is merely a metaphor for the internet. The internet is commonly represented as a cloud in a computer network [2].

The Antarex secondary dataset comprises trace data acquired from the homonymous experimental HPC system at ETH Zurich during fault injection to perform machine learning (ML) based fault detection experiments for HPC systems. The dataset is separated into two sections one for CPU and memory-related benchmark apps and fault programs, and another for hard drive-related applications and fault programs. Antarex dataset has four folders, one for each dataset block, namely CPU/Memory and HDD, in single-core and multi-core forms [3]. To generate the primary dataset the benchmark is the Weibull distribution approach. The Weibull distribution is also commonly used in reliability as a time-to-failure model. It extends the exponential model to incorporate failure rate functions that are not constant. This includes both rising and decreasing failure rate functions and has been used effectively to explain both initial burning failures and wear-out failures [4].

ML has played an active part in the resilient method area, mapping the recovery time to a function that can be improved (i.e. by converging the recovery time to a fraction of milliseconds). The recovery time will decrease as the system learns to deal with new errors. Researchers have recently become more interested in resilient approaches. The resilience of a system is defined as the speed with which it can recover and resume regular operation following a system outage or failure. Resilient methods include techniques dealing with the ability to respond to the client despite failure, monitoring of the system state, ability to learn and adapt from faults and predictions. In RSMs, the learning, and adaptation of a system based on Machine Learning. Techniques dealing with the capacity to react to the client despite failure, monitoring the system status, learning and adapting from faults, and predictions are examples of resilient approaches. The learning and adaptability of a system based on machine learning are used in resilient approaches [5].

## Materials and methods

### Literature review

Shahid et al. [6] suggested that CC has emerged as a distinct trend in recent years. As a result, distributed systems have evolved into large-scale computer networks. Cloud computing firms like IBM, Amazon, Yahoo, and Google offer cloud services to customers all over the world. Under this novel paradigm, end users are not required to install programs on their local PCs, instead, apps and services are delivered to them on demand.

Shahid et al. [7] investigate how cloud designs primarily involve the exchange of computing services among various users. Apps, hardware, and software systems are examples of shared resources. Cloud architectures are typically composed of three major layers: IaaS, SaaS, and PaaS. Faults can occur at any of these layers, but software-level recovery procedures are discovered and used.

Ahmed et al. [8] cloud technology is defined as the various software runtimes used on cloud computing platforms such as Hadoop, Dryad, and communication frameworks such as HDFS (Hadoop Distributed File System), Amazon S3, and others. Today, there are numerous cloud service apps available that are primarily used on CC platforms such as Nimbus and Eucalyptus, allowing various enterprises to build clouds to improve resource performance efficiency. The use of cloud computing system technology broadens several parallel processing capabilities.

Kamiri & Mariga [9] suggested, that ML is a subfield of artificial intelligence that deals with the creation of algorithms and procedures that allow a computer to learn and gain intelligence through experience. The research methodology used in machine learning research is critical because it influences the accuracy and dependability of the results. Machine learning models learn from historical data, which can be primary or secondary in nature. As a result, there is a vast knowledge base from which robots can learn and make decisions.

Sarker [10] based on sample input-output pairs, the process of learning a function that translates input to output was introduced. To infer a function, it uses labeled training data and a set of training examples. Supervised learning occurs when specific goals are specified to be achieved from a specific set of inputs, i.e., a task-driven method. Classification (data separation) and regression are the most commonly supervised tasks (fitting data). Supervised learning, for example, is used to predict the class label or sentiment of a piece of text, such as a tweet or a product review.

Butt et al. [11] investigate that ML is the logical evaluation of computations and quantifiable models used by computer systems to perform a particular attempt without the need for explicit instructions, based on models, and acceptance. It falls under the umbrella of computerized reasoning. Machine learning is so important in the cloud that it will be used by all clouds soon.

Sun et al. [12] suggested that ML has recently grown at a breakneck pace, attracting a large number of academics and practitioners. It has emerged as one of the most prominent research areas, with applications in a wide range of industries, including machine translation, speech recognition, image recognition, recommendation systems, and so on.

Kochhar et al. [13] the NB classifier are one of the most useful machine learning algorithms. The NB classifier is based on the Bayes theorem, which requires significant independence (nave) between qualities or features (predictors). Because it requires little work to develop and has no complicated repeating parameter setting or computation, the Naive Bayesian classification model is very useful for very large datasets. Despite its simplicity, the Nave Bayes classifier is one of the most widely used algorithms because it frequently outperforms and outperforms more complicated and refined classification algorithms.

Chang & Lin [14] proposed that LIBSVM is a Support Vector Machines library (SVM). The goal is to make applying SVM to applications as simple as possible for users. LIBSVM has been widely used in machine learning and other fields. LIBSVM has grown to be one of the most widely used SVM programs. LIBSVM provides support for a variety of SVM formulations for classification, regression, and distribution estimation. LIBSVM is widely used in numerous fields.

Mohamad [15] proposed that Proposed based on many independent factors, multinomial logistic regression is used to estimate the probability of multiple possible outcomes for a categorical dependent variable with more than two categories. The MLR model compares various categories using a combination of binary logit models. The multinomial logit model is composed of k-1 binary logit models that assess the influence of predictors on the likelihood of success in that category for k response variable categories.

C.R. LI & J. GUO [16] proposed that the SMO limits B to only two multipliers that can be calculated analytically and don’t require any extra matrix storage. There are two methods for determining which multipliers to optimize. The first heuristic prioritizes unbound multipliers that are more likely to violate the KKT specifications. The second choice heuristic, after selecting the first Lagrange multiplier, selects the second Lagrange multiplier that maximizes the difference between the two prediction errors. To save training time, the SMO technique is based on a single program multiple data (SPMD) paradigm. It divides the entire dataset into smaller subsets and uses several processors to update the error array of each subset in parallel.

Sen et al. [17] suggested that the K-nearest neighbor saves all available records and predicts the class of new occurrences in probability using similarity measures from the nearest neighbors. Unlike other classification techniques that construct a mapping function or internal model, this classification technique is known as a lazy learning method because it stores the data members in inefficient data structures such as hash tables, reducing the computation cost to check and apply the appropriate distance function between the new observation and all k number of different data points stored and then come to any conclusion about the label of the new data point. The results are generated by applying simple majority support to the K-nearest neighbors of each new data point.

Sarker [10] proposed a Random Forest classifier as a well-known ensemble classification approach used in machine learning and data science in a variety of application fields. This method uses a parallel ensemble, which involves fitting multiple decision tree classifiers to different data sets sub-samples in parallel with the conclusion or final result determined by majority voting or averages. Over-fitting is reduced as a result, and forecast accuracy and control are improved. As a result, the RF learning model with multiple decision trees frequently outperforms a single decision tree model. It employs a combination of bootstrap aggregation (bagging) and random feature selection to generate a series of decision trees with controlled variance.

Khazaei & Rezvani [18] this study proposes a multi-objective VM placement strategy to reduce energy costs and optimize scheduling. They present a modified memetic algorithm and compare its performance to baseline and state-of-the-art methods. The proposed method can lower energy costs, carbon footprints, SLA violations, and total IoT response time.

Bharany et al. [19] this paper critically examines FT techniques in CC systems and discusses the error, fault, and failure taxonomy. Furthermore, the purpose of this paper is to investigate many critical research topics and advanced techniques, such as AI, deep learning, the Internet of Things, and ML, that could be used as an intelligent FT strategy in the cloud environment.

Shahid et al. [20] this study compares the performance of existing load-balancing algorithms such as PSO, RR, ESCE, and throttled load balancing. Using a cloud analyst platform, this study provides a detailed performance evaluation of various load-balancing algorithms. Many of the previous papers mentioned in the literature focused on round robin and equally distributed current execution, as well as throttled load-balancing algorithms, and were based on efficiency and response time in virtual machines without taking into account the task-virtual machine relationship or the practical significance of the application. Table 5 in S1 Appendix shows a summary of the literature review in S1 Appendix section.

### Problem statement

Reliability is a continuous metric that changes with each computing step. One of the most important service characteristics is reliability, which must be met in cloud computing for a stable operation. The dependability of overall task completion is the result of specific activities, & for too many thousands or millions of computing operations, this can quickly become a fading variety. A cloud system’s reliability is an assessment of how effectively the cloud system provides the service to the user based on the criteria listed above [21].

There is a need to design & implement ML models that can resolve low accuracy and high fault prediction error issues by acquiring high accuracy, and less fault prediction error.

### The objective of the research

This study aims to help users with QoS in a CC environment by using ML to achieve high accuracy and lower fault prediction error. To accomplish this, the following goals must be met:

- There is a need to identify the best ML-based fault prediction model to improve the accuracy and failure prediction.
- Propose an ML-based model to address low accuracy and high fault prediction errors.

### Research methodology

This section focuses on research methodology. Classification, research design, data collection procedure, exploratory data analysis, data pre-processing, data analysis techniques, and proposed algorithm have all been thoroughly explained in this section.

#### Research design

The following research design has been followed:

#### Proposed model

**Fig 1:**
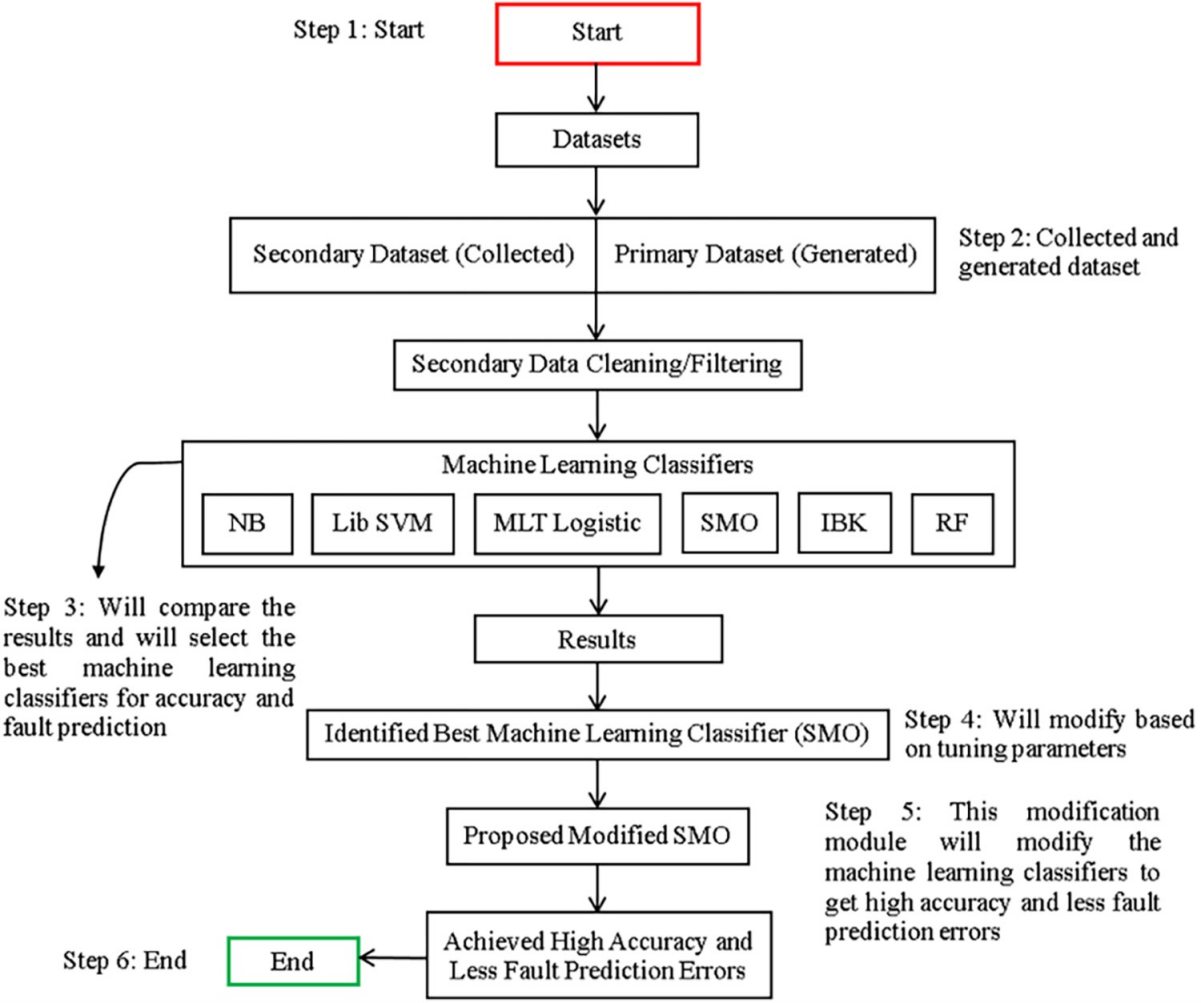
Implementation view of the research framework.

In this subsection, we propose our fault classification & prediction model. Fig 1 visualizes the whole research process reported in this paper. We will train our model using secondary & primary datasets and will do fault classification & prediction on the target datasets. By following this approach, we will identify which ML classifiers give the highest accuracy and less fault prediction error in terms of accuracy, prediction, & data validation by classes.

Furthermore, in this section, the data collection & data generated procedure has been explained in detail as well. Fig 1 shows the implementation view of the research framework.

(Fig 1) demonstrates that a genuine, competent, and effective solution has been designed to achieve more accuracy and less fault prediction error in cloud computing from the secondary and primary datasets.

#### Classification

Classification will be used to identify the fault status (True/False and Repair/Failure) with greater accuracy and lower fault prediction error. Classification is a supervised learning approach in machine learning that refers to a predictive modeling problem that predicts a class label for a given sample. It converts an input variable (X) to an output variable (f) as a goal, label, or category (Y). For example, spam detection by email service providers, such as "spam" and "not spam," could pose a classification challenge [11]. Various algorithms will be used to determine the best result among four directories of the secondary dataset for classification. For the primary dataset, we will use one CSV file to classify various algorithms to find the best algorithm for modification.

#### Acquired secondary data

We acquired (Antarex HPC Fault Dataset) secondary data through the ZENODO website and this dataset is published in articles. This dataset and all test environment details are publicly available for use by the community. The Antarex secondary dataset is based on trace data from the homonymous experimental HPC system at ETH Zurich during fault injection, which is used to undertake ML-based fault prediction studies for researchers [22].

Tables 6–9 in S1 Appendix shows the details of the CPU-Mem Mono (Instances 4005), CPU-Mem Multi (Instances 4380), HDD Mono (Instances 3244), and HDD Multicores (Instances 2493) dataset in S1 Appendix section. This dataset block has 8 attributes (timestamp, type, args, seqNum, duration, cores, error, and isFault) and various instances. These instance types are numeric and nominal bases [22].

#### Exploratory data analysis on secondary dataset

We performed Exploratory Data Analysis (EDA) on Antarex secondary dataset. The purpose of EDA is essential to tackle specific tasks such as spotting missing and erroneous data, mapping and understanding the underlying structure of your data, and identifying the most important variables in the dataset. The dataset is separated into two sections, one for CPU and memory-related benchmark apps and fault programs, and another for hard drive-related applications and fault programs. Antarex datasets have four folders, one for each dataset block, namely CPU/Memory and HDD, in single-core and multi-core forms [22].

#### Data pre-processing on the secondary dataset

Data pre-processing is necessary before applying ML algorithms to secondary datasets. This dataset has duplicate values in 3 attributes named args, seqNum, and duration. Furthermore, this dataset has some none values and empty rows. All duplicate values, none values, and empty rows are removed using the Remove Duplicates option in excel. Tables 10–13 in S1 Appendix shows the details after applying data pre-processing of the CPU-Mem Mono (Instances 1740), CPU-Mem Multi (Instances 1408), HDD Mono (Instances 568), and HDD Multicores (Instances 551) in S1 Appendix section.

#### Generated primary data

We have generated a primary dataset through the Weibull distribution approach. The Weibull distribution is also often employed as a time-to-failure model for reliability. It extends the exponential model by including non-constant failure rate functions. This contains both rising and falling failure rate curves and has been successfully utilized to explain both initial burnings and wear-out failures [4]. We have coded different parameters in the java platform for primary data generated using the Weibull distribution approach. Table 1 is a summary of the parameters of the primary dataset generation. The primary dataset is shown in Table 2.

**Table 1:** Overview of the parameters of primary data generation.

User	Port No	Host No	Network Host	Distribution
1	16	192	Mips, Ram, Storage, and Bandwidth	Weibull (this includes both rising and decreasing failure rate functions).

**Table 2:** Short overview of the primary dataset.

FHID	HFTIME	LFT	DIS	DISHT	FTIME/RTIME	STATUS
328	1	-74003	Weibull	0.75:20	11965	Failure
328	1	-74003	Weibull	0.75:20	22765	Repair
453	2	-280036	Weibull	0.75:20	16299	Failure
453	2	-280036	Weibull	0.75:20	27099	Repair
227	1	-133119	Weibull	0.75:20	8498	Failure
227	1	-133119	Weibull	0.75:20	19298	Repair
190	1	-18201	Weibull	0.75:20	7236	Failure
190	1	-18201	Weibull	0.75:20	18036	Repair
688	3	-17508	Weibull	0.75:20	24386	Failure
688	3	-17508	Weibull	0.75:20	35186	Repair
333	1	-143411	Weibull	0.75:20	12150	Failure
333	1	-143411	Weibull	0.75:20	22950	Repair
848	3	-123990	Weibull	0.75:20	29921	Failure
848	3	-123990	Weibull	0.75:20	40721	Repair
1013	3	-29678	Weibull	0.75:20	35590	Failure
1013	3	-29678	Weibull	0.75:20	46390	Repair
454	2	-14341	Weibull	0.75:20	16339	Failure
454	2	-14341	Weibull	0.75:20	27139	Repair
992	3	-15158	Weibull	0.75:20	34875	Failure
992	3	-15158	Weibull	0.75:20	45675	Repair
343	2	-105315	Weibull	0.75:20	12513	Failure
343	2	-105315	Weibull	0.75:20	23313	Repair
277	1	-35992	Weibull	0.75:20	10225	Failure
277	1	-35992	Weibull	0.75:20	21025	Repair
186	1	-288717	Weibull	0.75:20	7068	Failure
186	1	-288717	Weibull	0.75:20	17868	Repair
411	2	-20145	Weibull	0.75:20	14836	Failure
411	2	-20145	Weibull	0.75:20	25636	Repair

Table 2 shows the details of the primary dataset. This primary dataset has 7 attributes (Failure Host ID (FHID), Host Failure Time (HFT), Last Failure Time (LFT), Distribution (Dis), Distribution Happen Time (DHT), Failure Time/Repair Time (FTime/RTime), and Status) and total (1400) instances. These instance types are numeric and nominal bases.

### Data analysis techniques

Different ML-based techniques have been used in this study for fault classification and prediction. Fault classification and prediction are carried out using various classifiers from NB, LibSVM, MLR, SMO, KNN, and RF algorithms.

#### Naïve bayes

**Fig 2:**
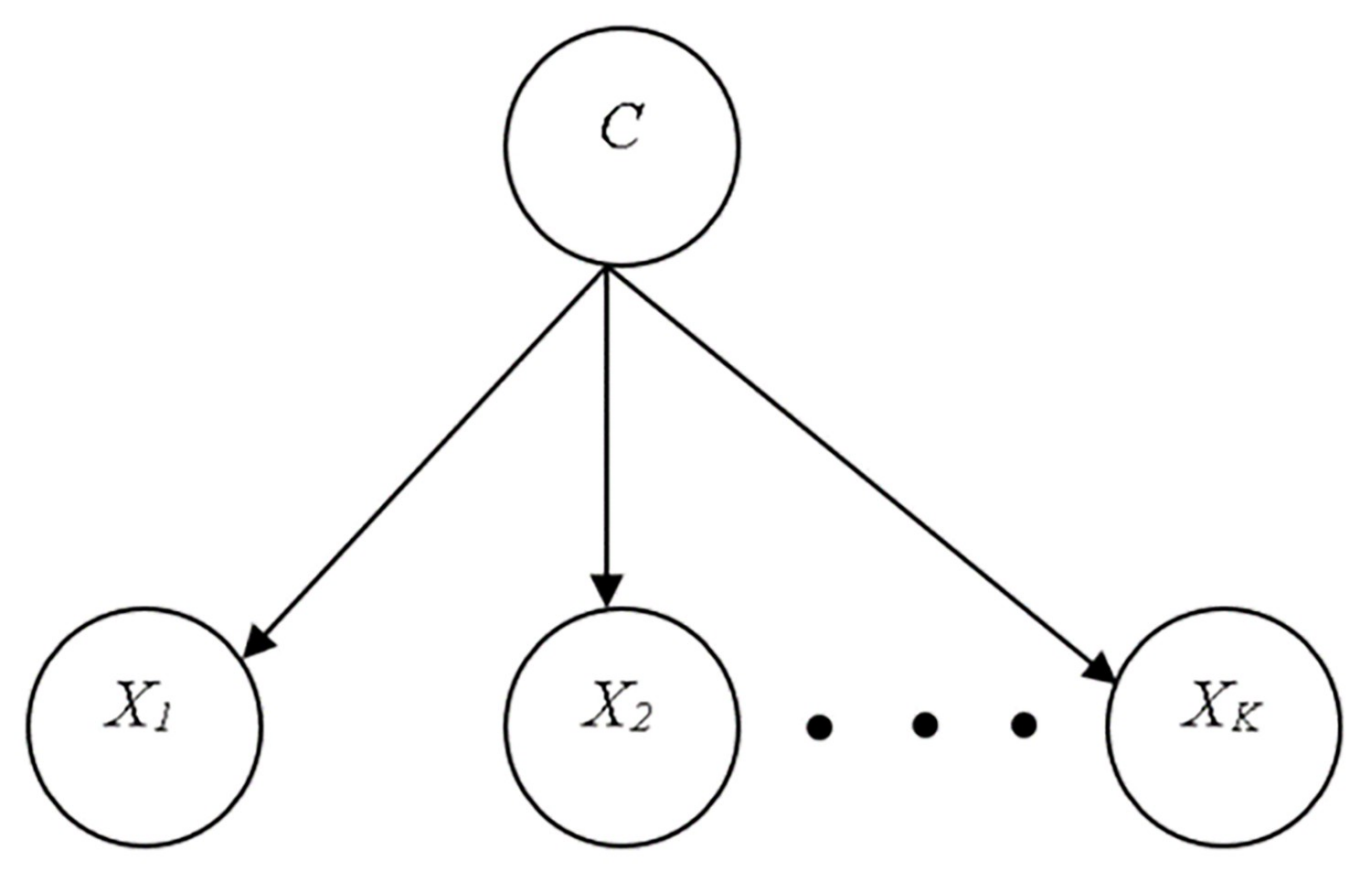
Naïve bayes classifier [23].

The NB classifier represents, employs, and learns probabilistic knowledge with well-defined semantics. The method is intended for supervised induction tasks where the performance goal is to correctly predict the class of test cases and the training examples include class information. A naive classifier is a type of Bayesian network that is built on two basic simplifying assumptions. It assumes, in particular, that the predictive qualities are conditionally independent of the class and that no hidden or latent features influence the prediction process. As a result, the graphic shape of a naive Bayesian classifier is shown in (Fig 2), with all arcs pointing from the class attribute to the observable, predictive attributes [23].

The Bayes’ rule is used in Eqs 1 to 3 to compute the probability of each class given a vector of observed values for the predictive qualities and then predicts the most likely class.


$$\begin{array}{c} p(C=c|X=x)=\frac{p(C=c)p(X=x|C=c)}{p(x=x)}\\ p(X=x|C=c)=p(˄iXi=xi|C=c)\\ =\Pi i\;p(Xi=xi|C=c) \end{array}$$ (1)

Let C be the random variable representing the class of an instance, & X be a vector of random variables representing the observed attribute values. Furthermore, let c denote a specific class label & x denote an observed attribute value vector.


p=(X=xC=c)=g(x;μc,σc),where (2)

$$g(x;\;\mu ,\sigma )=x=\frac{1}{\sqrt{2\pi \sigma }}e-\frac{n(x-\sigma {)}^{2}}{2x\sigma^{2}}$$ (3)

For continuous attributes, we can write the probability density function for a normal (or Gaussian) distribution.

#### Library support vector machine

**Fig 3:**
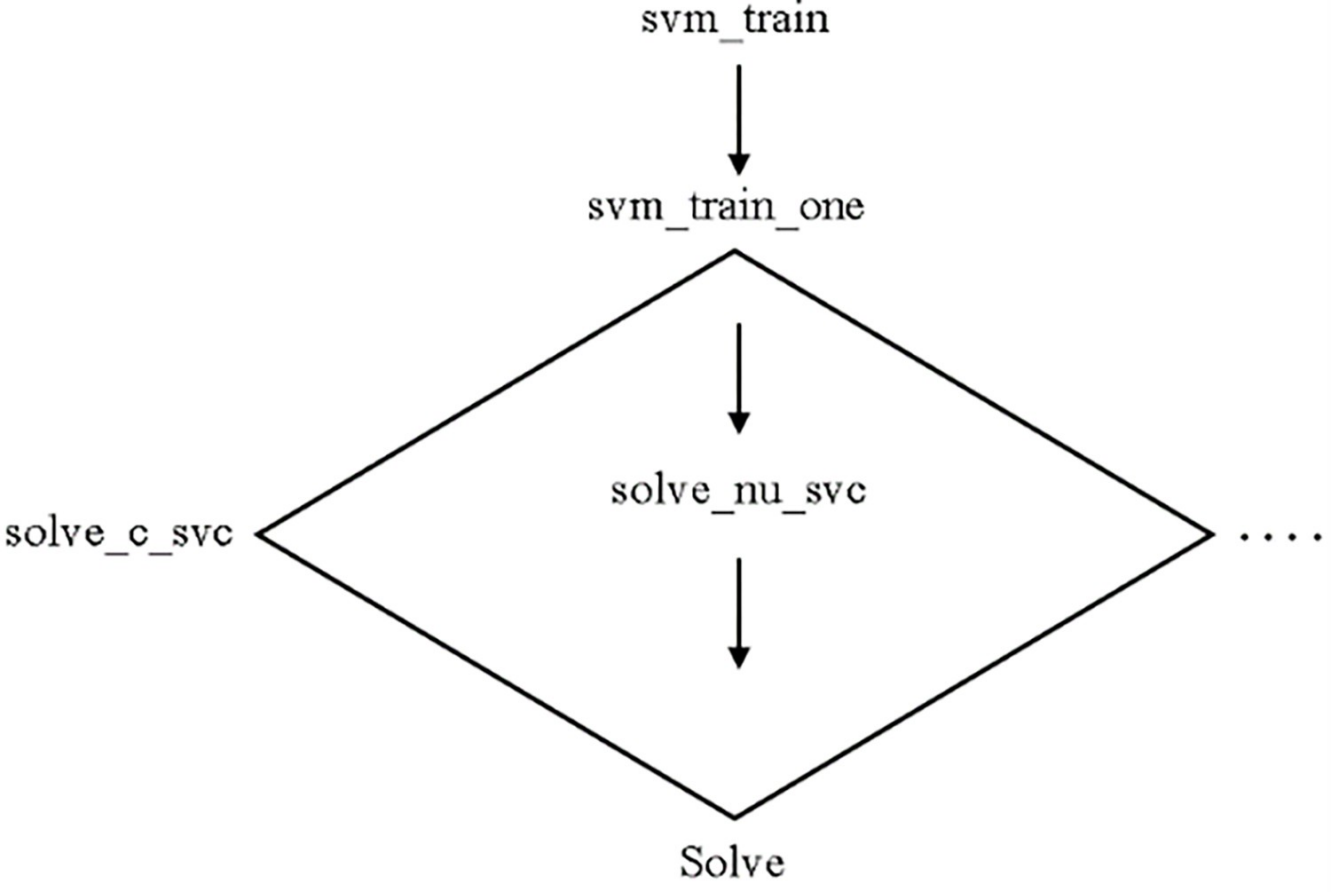
LibSVM classifier [14].

LIBSVM is a library for SVMs. The goal is to make it as simple as possible for users to apply SVM to their applications. LIBSVM has been widely adopted in ML and a variety of other fields. LIBSVM is frequently used in two steps: training a data set to generate a model, followed by using the model to predict information from a testing data set. LIBSVM supports numerous SVM formulations for classification, regression, and distribution estimation. (Fig 3) depicts the code organization of LIBSVM for training [14].

In Eq 4 where e = [1,…, 1]T is the vector of all ones, Q is an l by l positive semidefinite matrix, Qij ≡ yiyjK(xi, xj), and the kernel function is as follows:

K(xi,xj)(xi)T(xj) (4)

#### Multinomial logistic regression

**Fig 4:**
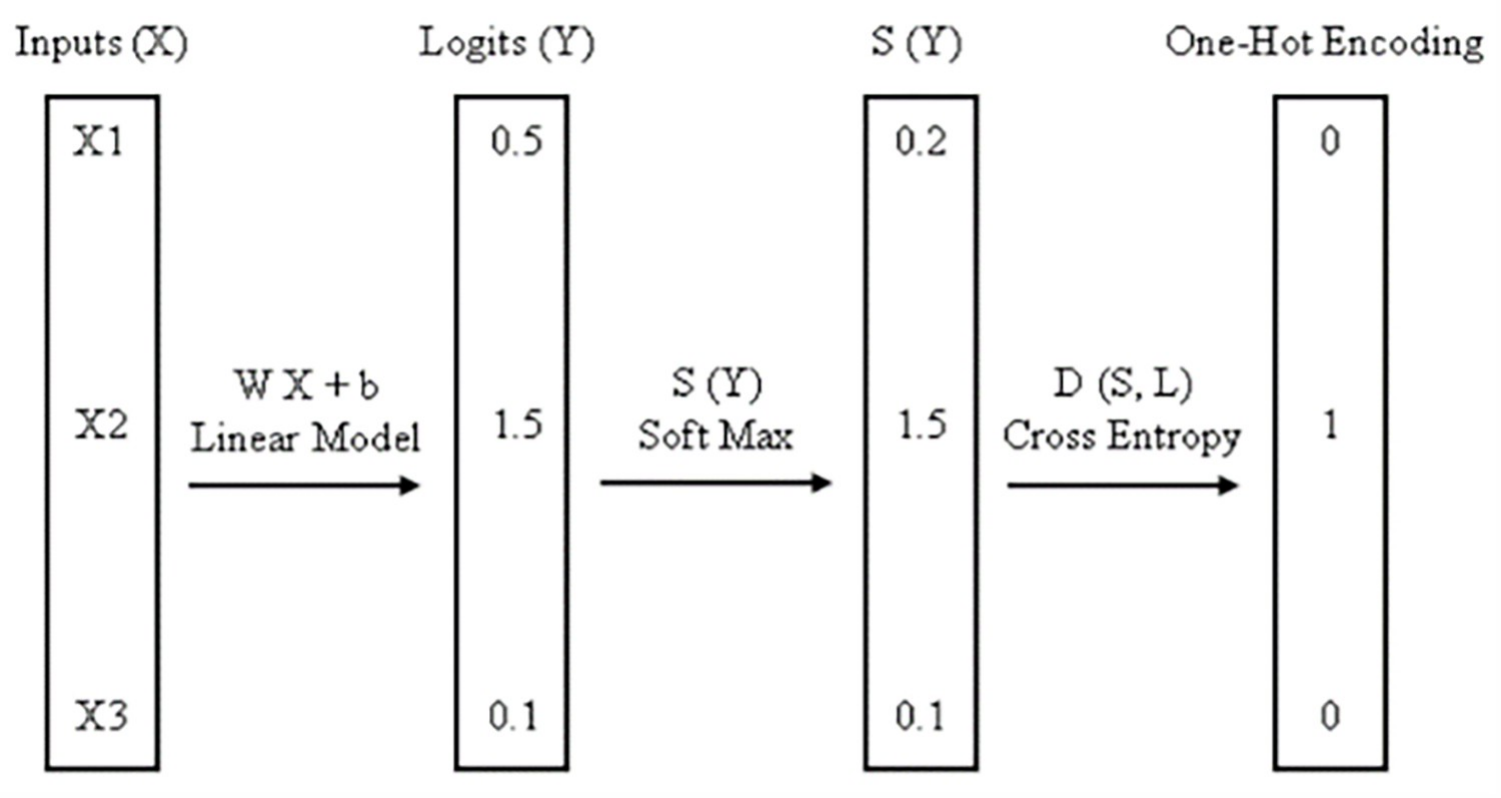
Multinomial logistic classifier [25].


Softmax is an abbreviation for MLR. Because of the hypothesis function it employs, regression is a supervised learning technique that can be used to solve a variety of problems, including text categorization. It is a regression model that applies logistic regression to classification problems with multiple possible outcomes [24]. The Multinomial Logistic Classifier is depicted in (Fig 4).

$$\begin{array}{c} L_{1}(w)=\frac{\lambda }{2}\sum_{k=1}^{K}||w_{k}{\left|\right|}^{2}-\frac{1}{N}\sum_{i=1}^{N}\sum_{k=1}^{K}y_{ikw_{k}^{T}x_{i}}\\ +\frac{1}{N}\sum_{i=1}^{N}log(\sum_{k=1}^{K}exp(w_{k}^{T}x_{i})) \end{array}$$ (5)

In Eq 5 MLR is employed where the objective function of the classifier is given as above.

### Sequential minimal optimization

**Fig 5:**
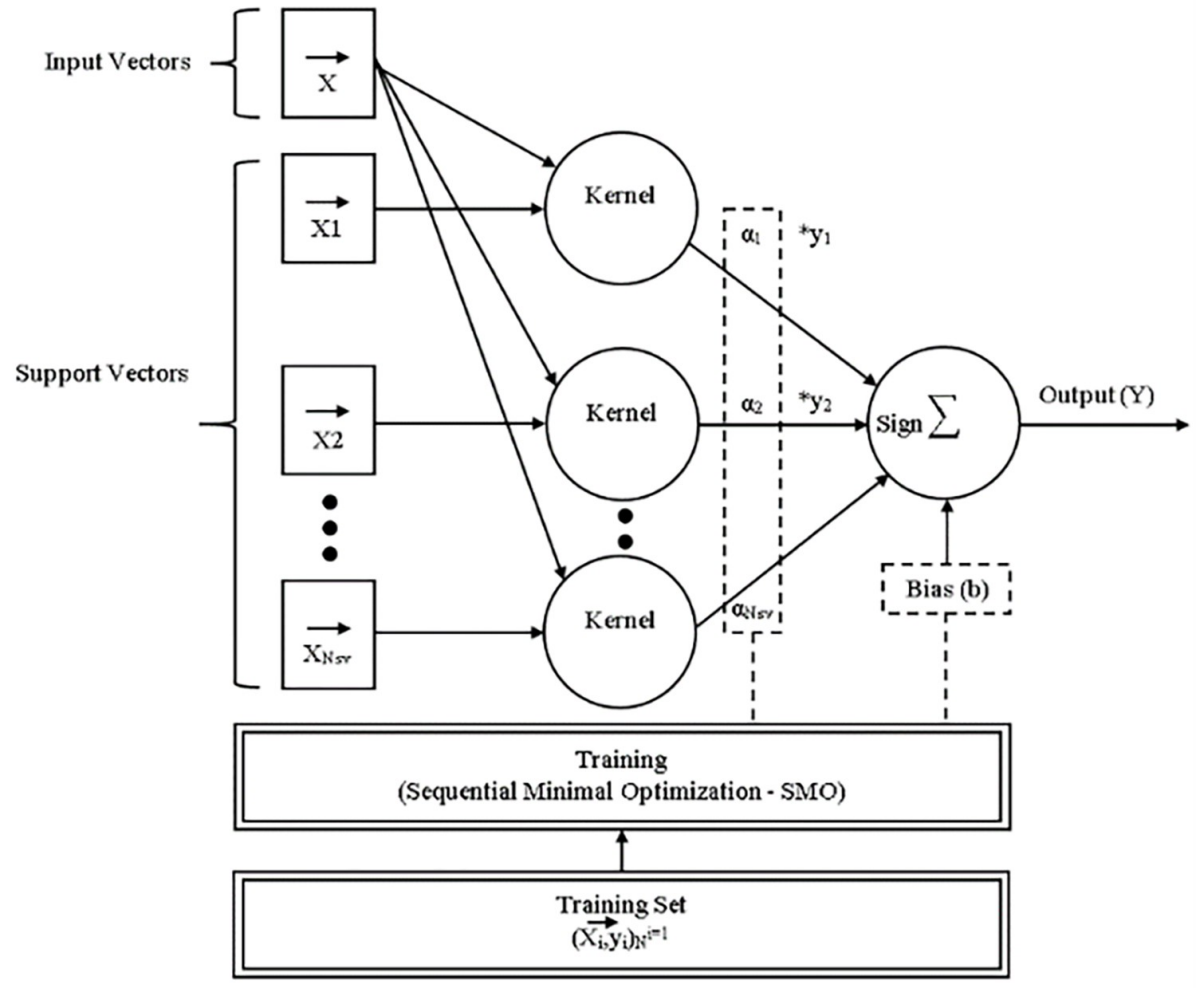
The general architecture of SMO inference and training [27].

To train an SVM, a very large quadratic programming (QP) optimization problem must be solved. SMO divides the enormous QP problem into the smallest feasible QP problems. These minor QP issues are handled analytically, which eliminates the need for a time-consuming numerical QP optimization as an inner loop. SMO’s memory requirements scale linearly with training set size, allowing it to handle extremely large training sets. SMO scales the training set size for various test problems somewhere between linear and quadratic because matrix computation is avoided, whereas the traditional chunking SVM technique scales the training set size somewhere between linear and cubic. Because SVM evaluation consumes the majority of SMO’s computing time, SMO is the fastest for linear SVMs and sparse data sets. SMO can be more than 1000 times faster than chunking in real-world sparse data collections [26]. (Fig 5) depicts the overall architecture of SMO inference and training.

In Eqs 6 to 8 the QP problem for training an SVM is:

$$w(\lambda )=\sum_{i=1}^{I}\lambda_{i}-\frac{1}{2}\sum_{i=1}^{I}\sum_{i=1}^{I}yiyjK(x_{i}-x_{j})\lambda_{i}\lambda_{j}$$ (6)

$$0\leq \lambda_{i}\leq C,i=1,\ldots ,1,$$ (7)

$$\sum_{i=l}^{l}y_{i}\lambda_{i}=0$$ (8)

In Eq 6 the QP problem for training an SVM is maximized and subject to 7 & 8.

#### K-nearest neighbor

**Fig 6:**
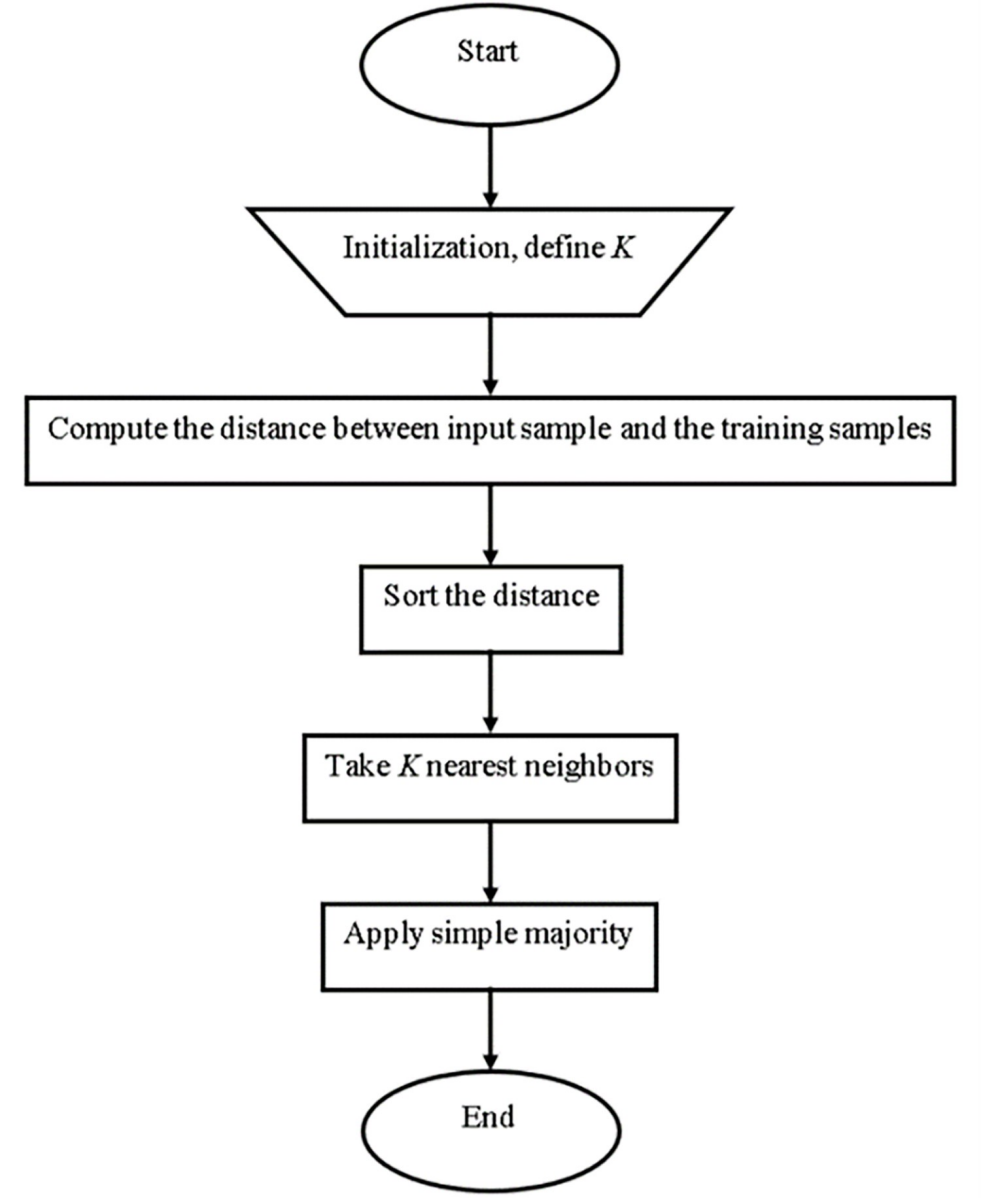
KNN architecture [29].

The KNN classification method is widely used. It is widely used because of its simplicity and quick calculation time [28]. The choice of value k is critical in this method, as shown in (Fig 6). The two parameters that must be accessible to different k values are training and validation error rates [29].

- Determine the parameter K defining the number of nearest neighbors [29].
- Calculate the distance between the query and all training examples [29].
- Using the kth minimum, sort the distance and find the closest neighbors [29].
- Gather the closest neighbors’ category [29].
- Use the majority in the category of nearest neighbors as the instance’s prediction value [29].

KNN classifiers such as Fine, Medium, Coarse, Cosine, Cubic, and Weighted KNN use data to categorize new data points based on similarity measurements.

- **Fine and Medium KNN:** The Fine and Medium KNN algorithms use the Euclidean distance function to calculate the nearest neighbors, as shown in Eqs 9 and 10.

$$d=\sqrt{(x_{1}-y_{1}{)}^{2}+(x_{2}-y_{2}{)}^{2}}$$ (9)

$$\sqrt{\sum_{i=1}^{k}\left(x_{i}-y_{i}\right)}$$ (10)

To calculate the NNs, the Fine and Medium KNN algorithms employ the Euclidean distance function, as indicated in Eqs 9 and 10.

#### Random forest

**Fig 7:**
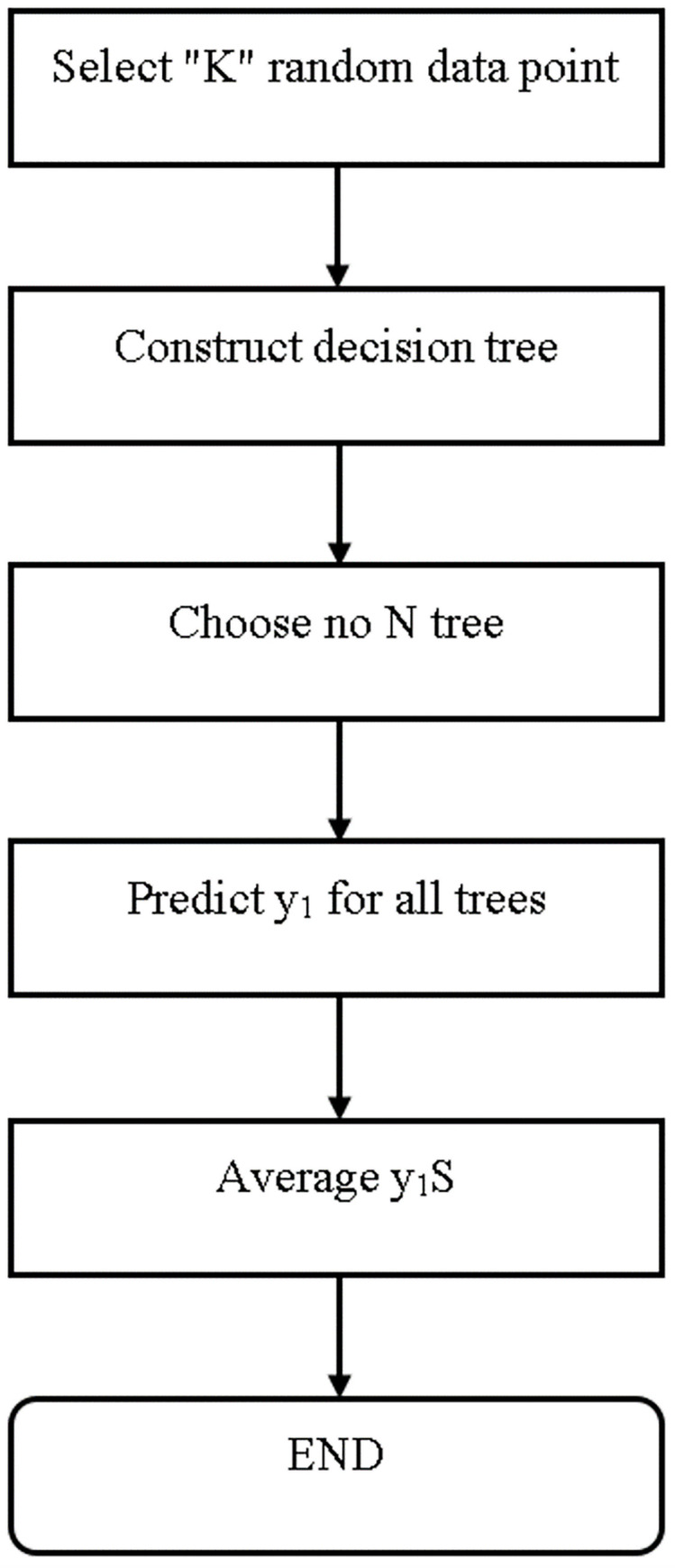
Flowchart of random forest classifier [30].

This method generates a large number of collaborative decision trees. In this algorithm, decision trees serve as pillars. RF is a set of decision trees that were defined during the pre-processing stage. After constructing many trees, the best feature from a random subset of features is chosen. Another idea generated by the decision tree algorithm is the creation of a decision tree. As a result, these trees combine to form a random forest, which is used to classify new objects based on the input vector. Each built decision tree is used to categorize. (Fig 7) depicts the flowchart of a random forest classifier [30].

The mathematical formula for random forest classifiers is shown below in Eq 11.


$$\begin{array}{c} n_{ij}=w_{i}C_{j}-w_{leftj}C_{leftj}-w_{rightj}C_{rightj}\\ n_{i}\;sub(j)=the\;importance\;of\;node\;j\\ w\;sub(j)=weighted\;number\;of\;samples\;reaching\;node\;j\\ C\;sub(j)=the\;impurity\;value\;of\;node\;j\\ left(j)=child\;node\;from\;left\;split\;on\;node\;j\\ right(j)=child\;node\;from\;right\;split\;on\;node\;j \end{array}$$ (11)

#### Parameters configuration of ML classifiers

ML classifiers have been configured by applying different parameters to achieve accuracy and fault prediction by class. Table 3 shows the different parameters of ML classifiers with values.

**Table 3:** Parameter configuration of ML classifiers.

Classifiers	Configuration Parameters	Values
Naïve Bayes	Batch size	100
	Debug	False
	Display model in old format	False
	Do not check capabilities	False
	Num decimal places	2
	Use kernel estimator	False
	Use supervised discretization	False
Library Support Vector Machine	SVM type	C-SVC (Classification)
	Degree	3
	EPS	0.001
	Gamma	0.0
	Kernel type	radial basic function
	Normalize	False
	Seed	1
Multinomial Logistic Regression	Batch size	100
	Do not check capabilities	False
	Num decimal places	4
	Ridge	1.0 × 10−^8^
Sequential Minimal Optimization	C complexity parameter	1.0
	Epsilon	1.0 × 10−^12^
	Filter type	normalize training data
	Kernel	Polykernel −10 1.0−C 25,007
	Num folds	1
	Random seed	1
	Tolerance parameter	0.001
K-Nearest Neighbor	KNN	1
	Batch size	100
	Cross validate	False
	Nearest neighbor search algorithm	linear NN search
Random Forest	Batch size	100
	Max depth	0
	Num decimal places	2
	Num features	0
	Num iterations	100
	Seed	1

### Modified sequential minimal optimization

**Fig 8:**
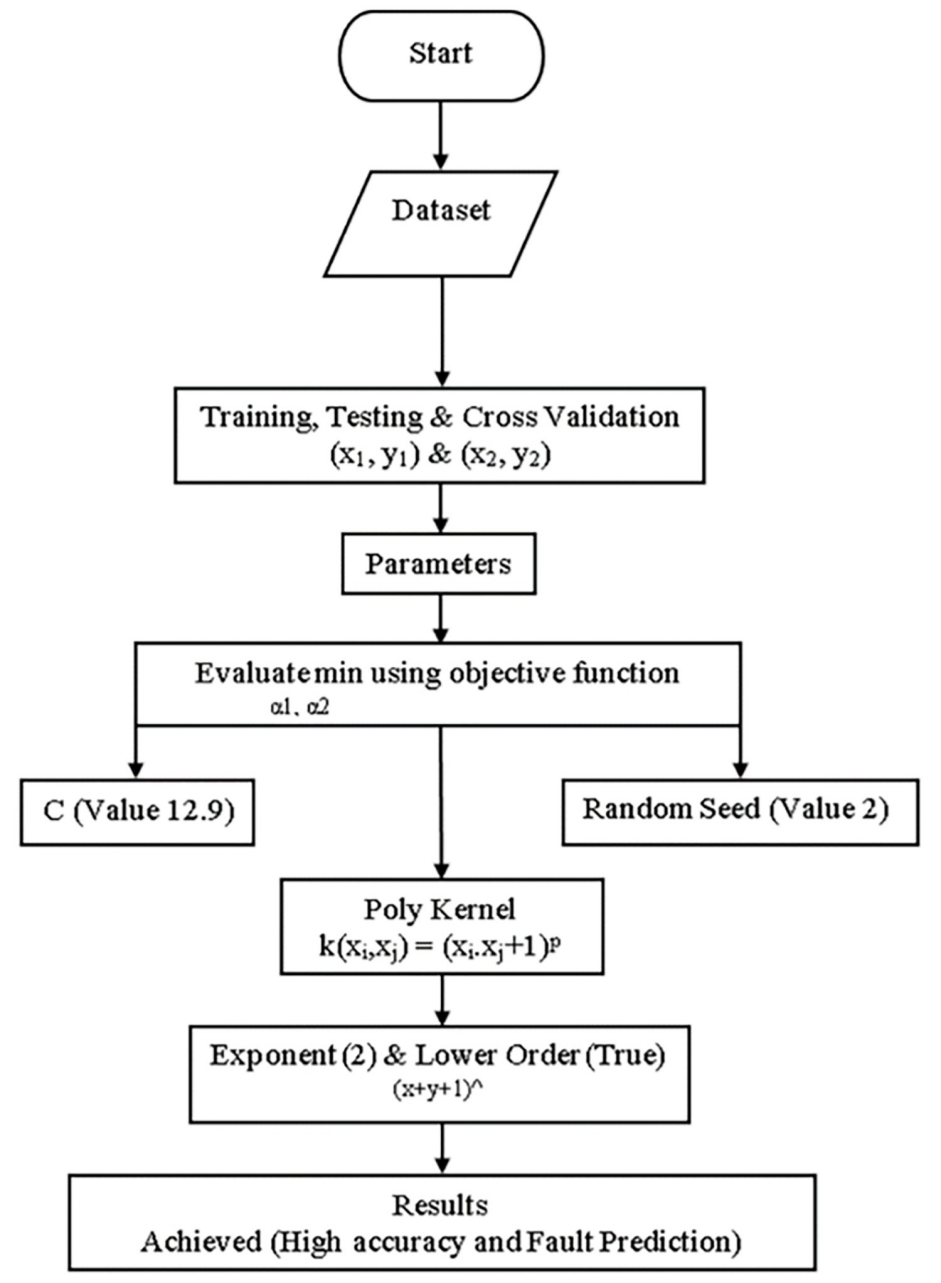
Block diagram of MSMO classifier.

The original SMO algorithm has low accuracy & a high fault prediction error. This research has to resolve low accuracy & high fault prediction errors by acquiring high accuracy with less fault prediction error from MSMO. The block diagram of an MSMO classifier is shown in (Fig 8). High accuracy & less fault prediction errors are based on the primary dataset that has been generated. High accuracy & less fault prediction error have been evaluated min α1, α2 using an objective function. High accuracy & less fault prediction error have been made by applying objective functions through algorithm parameters & kernel parameters. The C parameter has been determined as a trade-off between fitting the training data & maximizing the separating margin. C has a value between 0.01 & 100. The random seed has been set at 2. The only parameter for the polynomial kernel is the exponent, which has controlled the degree of the polynomial. By default, the kernel has computed the exponent as (x * y).

The mathematical formula for modified sequential minimal optimization classifiers is shown below in Eqs 12–15.


(x1,y1)and(x2,y2) (12)

In Eq 12 training, testing & validation is defined as above.


α1.α2 (13)

In Eq 13 the objective function is defined as above.


$$k(x_{i,}x_{j})=(x_{i}*x_{j}+1^{p})$$ (14)

In Eq 14 polynomial kernel is defined as above.


(x+y+1)^ (15)

In Eq 15 exponent & lower order are defined as above.

## Results and findings

This section includes data analysis and classification results from NB, LibSVM, MLR, SMO, and RF with a confusion matrix, as well as graphical representations of the results. Finally, the MSMO results, which are the main algorithm of this research study, are included here. This study compares conventional ML algorithms to achieve high accuracy and less fault prediction errors.

The secondary dataset archive contains four directories, one for each dataset block, namely CPU/Memory and HDD in single-core and multi-core variants [3]. Based on the results, a significant difference has been observed in the four directories of the secondary dataset, with CPU-Mem Multi cores outperforming the remaining directories such as CPU-Mem Mono, HDD Mono, and HDD Multi.

According to the comparisons, the primary dataset outperforms the secondary dataset, so in this study, the primary dataset results were sufficient to consider when modifying the ML algorithm.

Data was trained on 80/20, 70/30, and 5-fold cross-validation using NB, LibSVM, MLR, SMO, KNN, and RF classifiers, and the desired classification results were obtained (Secondary & Primary). The results of NB, LibSVM, MLR, SMO, KNN, and RF are compared in terms of accuracy, fault prediction error, and data validation by class using Eqs 16 to 26. Results from a secondary dataset (CPU-Mem Multi) demonstrated that NB outperformed LibSVM, MLR, SMO, KNN, and RF. Furthermore, the results of the primary dataset demonstrated that RF outperformed, but the time complexity is poor. According to the primary dataset results, RF and SMO have minor point value differences, but SMO has good time complexity. The software environment we used is WEKA 3.8.6 with Remove Percentage Filter.


$$Accuracy=\frac{\mathrm{TP}+\mathrm{TN}}{\mathrm{TP}+\mathrm{TN}+\mathrm{FP}+\mathrm{FN}}$$ (16)

In Eq 16 the accuracy is defined as above.


$$Recall\;or\;True‐Positive\;Rate=\frac{\mathrm{TP}}{\mathrm{TP}+\mathrm{FN}}$$ (17)

In Eq 17 the recall or true positive rate is defined as above.


$$True‐Negative\;Rate=\frac{\mathrm{TN}}{\mathrm{TN}+\mathrm{FP}}$$ (18)

In Eq 18 the true negative rate is defined as above.


$$Precision=\frac{\mathrm{TP}}{\mathrm{TP}+\mathrm{FP}}$$ (19)

In Eq 19 the precision is defined as above.


$$False‐Positive\;Rate=\frac{\mathrm{FP}}{\mathrm{TN}+\mathrm{FP}}$$ (20)

In Eq 20 the false positive rate is defined as above.


$$MCC=\frac{\mathrm{TP}.\mathrm{TN}-\mathrm{FP}.\mathrm{FN}}{\surd (\mathrm{TP}+\mathrm{FP})(\mathrm{TP}+\mathrm{FN})(\mathrm{TN}+\mathrm{FP})(\mathrm{TN}+\mathrm{FN})}$$ (21)

In Eq 21 the Matthews correlation coefficient is defined as above.


$$F‐Measure=\frac{2\mathrm{PPV}\times \mathrm{TPR}}{\mathrm{PPV}+\mathrm{TPR}}$$ (22)

In Eq 22 the F-measure is defined as above.

- The RMSE is a commonly used measure of the difference between predicted & observed values by a model or estimator [31].
- MAE is a distinct measure of two continuous variables [31].
- The relative absolute error normalizes the total absolute error by dividing it by the total absolute error of the simple predictor [32].
- The relative squared error normalizes the total squared error by dividing it by the simple predictor’s total squared error [32].

$$RMSE=\sqrt{\frac{1}{n}\sum_{i=1}^{n}\left(y_{i}-\hat{yi}\right)}$$ (23)

In Eq 23 the RMSE is defined as above.


$$MAE=\frac{1}{n}\sum_{i=1}^{n}\left|y_{i}-\hat{y_{i}}\right|$$ (24)

In Eq 24 the MAE is defined as above.


$$E_{i}=\frac{\sum_{j=1}^{n}\left|P_{\left(ij\right)}-T_{j}\right|}{\sum_{j=1}^{n}\left|T_{j}-\bar{T}\right|}$$ (25)

In Eq 25 the RAE is defined as above.


$$E_{i}=\sqrt{\frac{\sum_{j=1}^{n}{\left(P_{\left(ij\right)}-T_{j}\right)}^{2}}{\sum_{j=1}^{n}{\left(T_{j}-\bar{T}\right)}^{2}}}$$ (26)

In Eq 26 the RSE is defined as above.

### Comparison of classification models on a secondary dataset

We are presenting results associated with different classifiers using ISFAULT in secondary data and STATUS in primary data. As classification models, we opted for an NB, LibSVM, MLR, RF, KNN, and SMO with a poly kernel.

**Fig 9:**
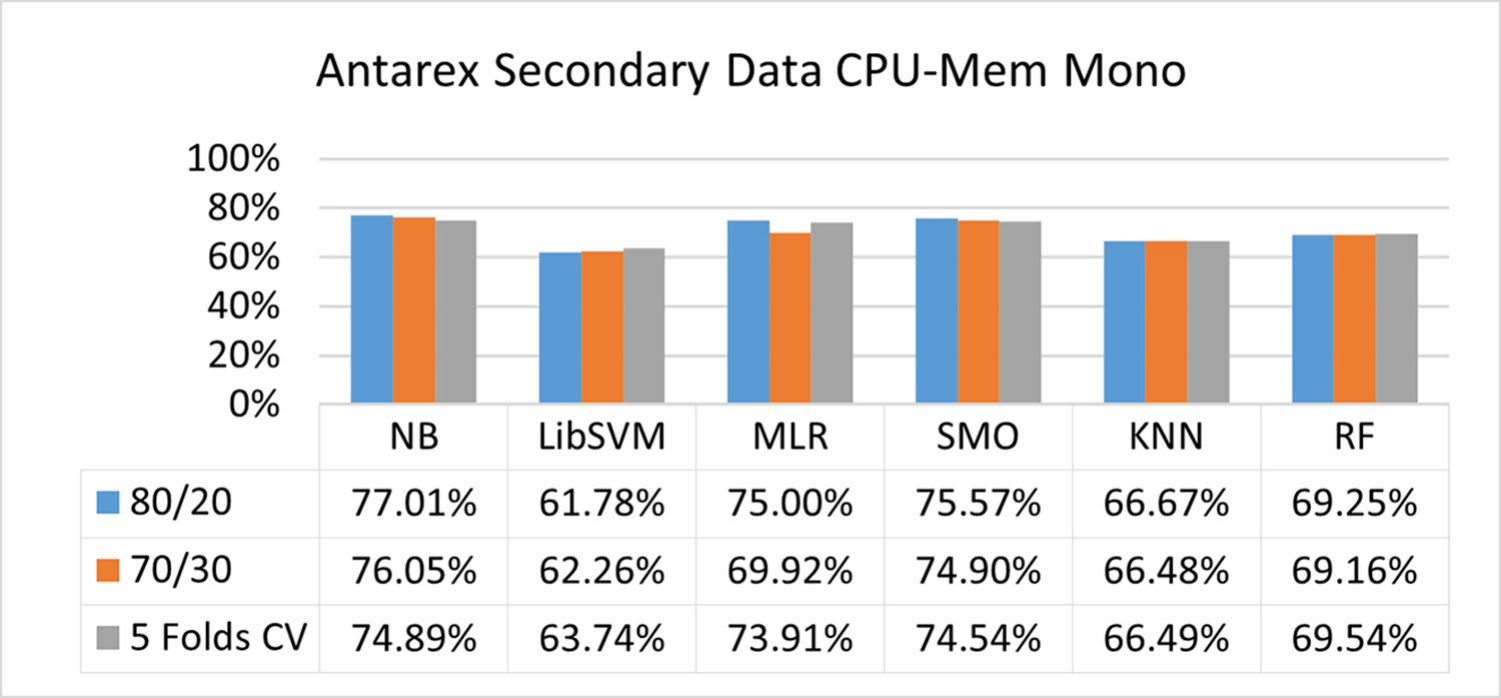
Accuracy by class (true/false) of CPU-mem mono on ML classifiers.

**Fig 10:**
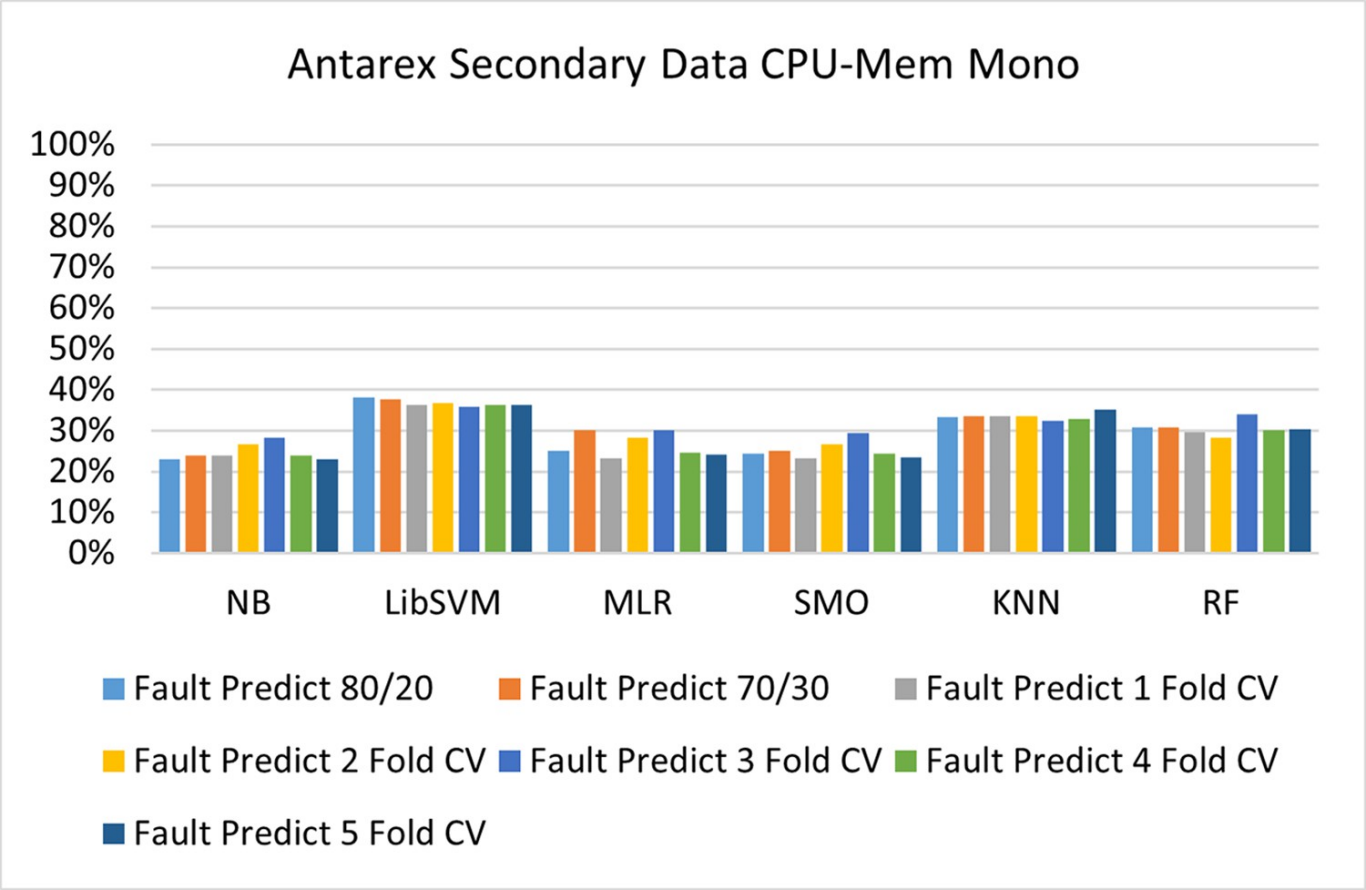
Fault prediction by class (true/false) of CPU-mem mono on ML classifiers.

**Fig 11:**
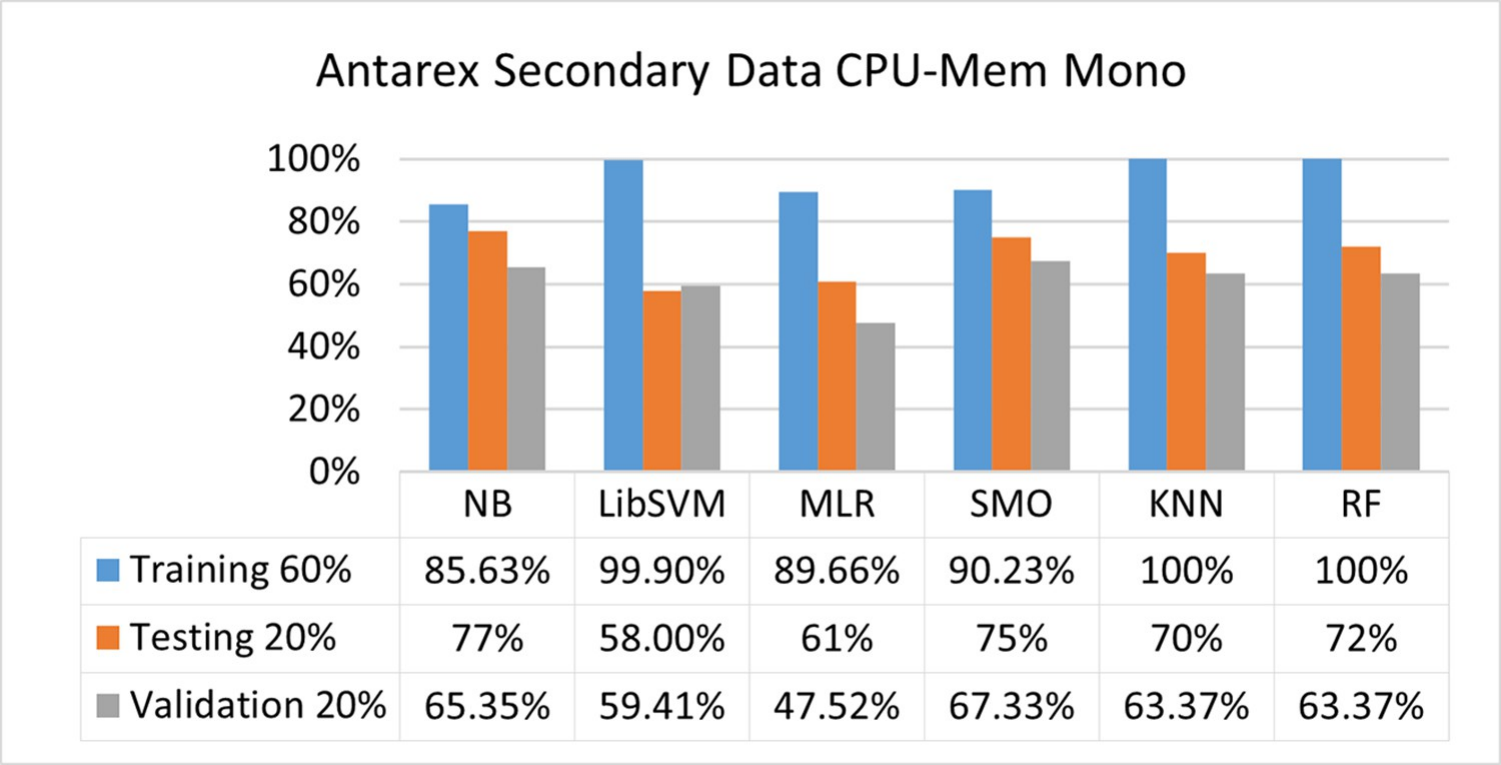
Accuracy by class (true/false) of CPU-mem mono on ML classifiers related to data validation results.

**Fig 12:**
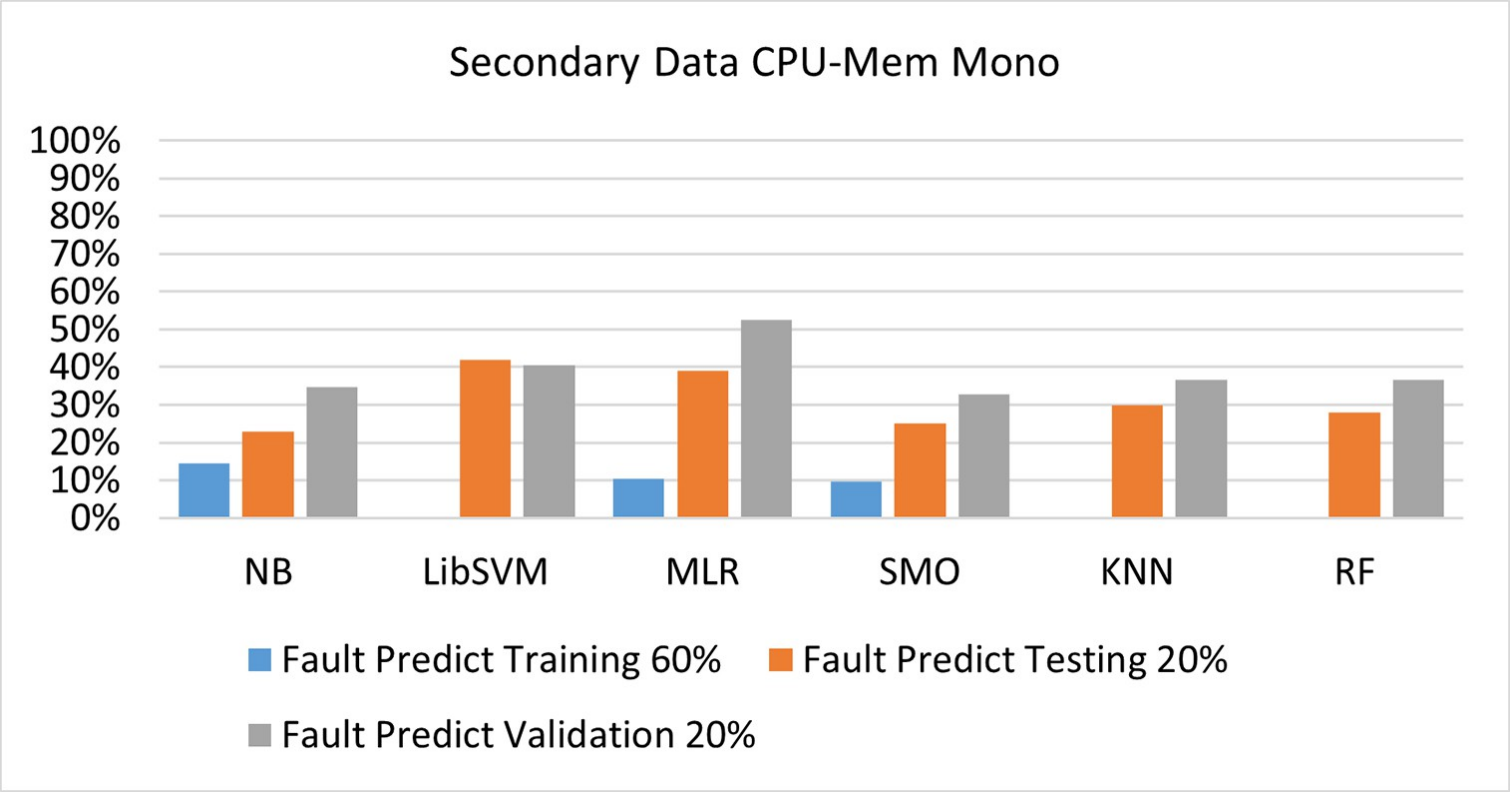
Fault prediction by class (true/false) of CPU-mem mono on ML classifiers related to data validation results.

**Fig 13:**
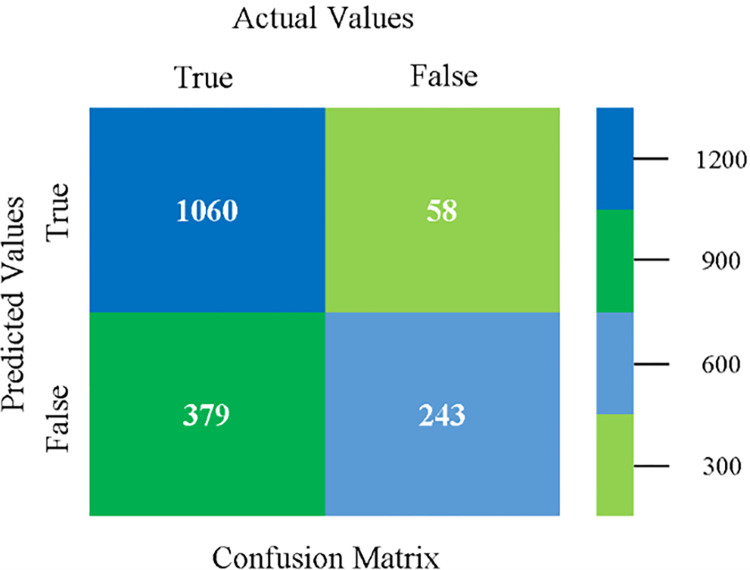
Confusion matrix of NB classifier based on CPU-mem mono in accuracy & fault prediction.

**Fig 14:**
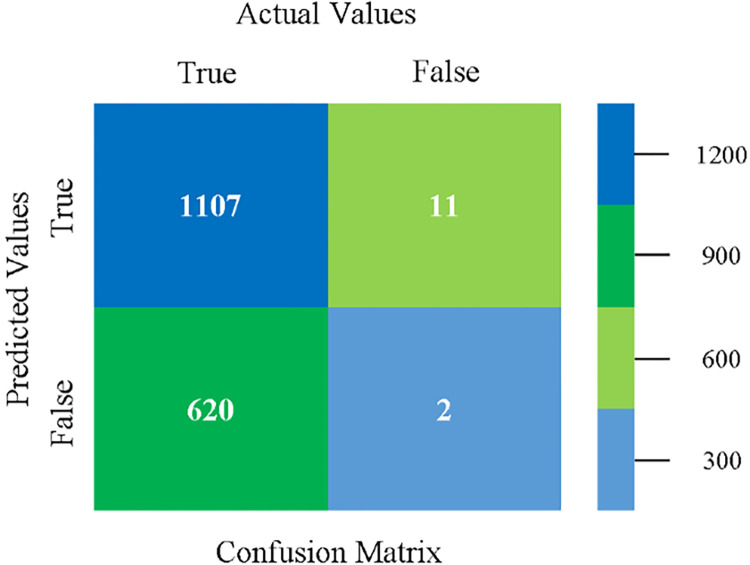
Confusion matrix of LIBSVM classifier based on CPU-mem mono in accuracy & fault prediction.

**Fig 15:**
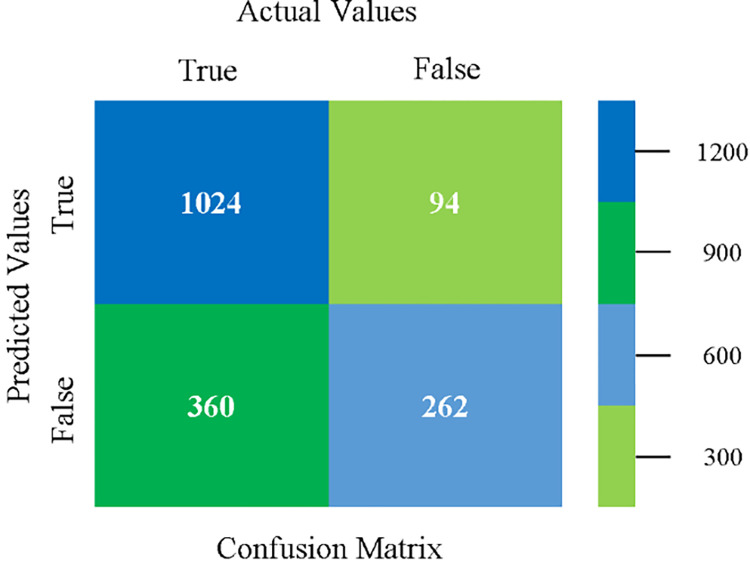
Confusion matrix of MLR classifier based on CPU-mem mono in accuracy & fault prediction.

**Fig 16:**
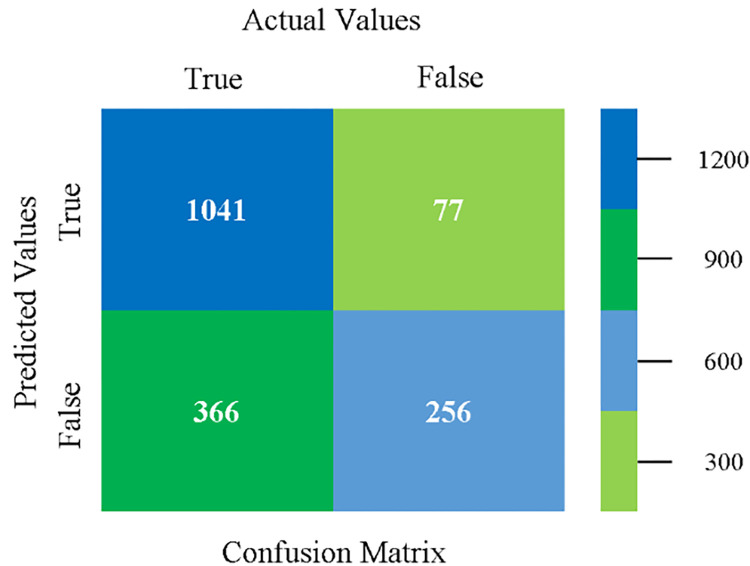
Confusion matrix of SMO classifier based on CPU-mem mono in accuracy & fault prediction.

**Fig 17:**
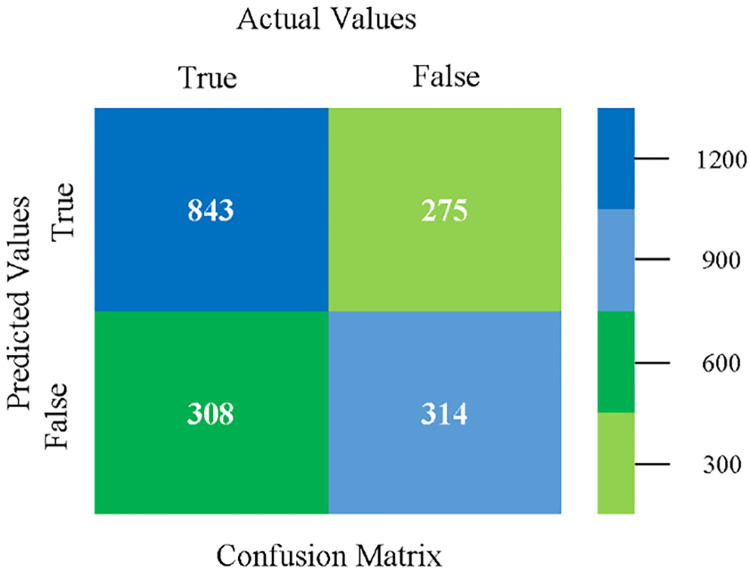
Confusion matrix of KNN classifier based on CPU-mem mono in accuracy & fault prediction.

**Fig 18:**
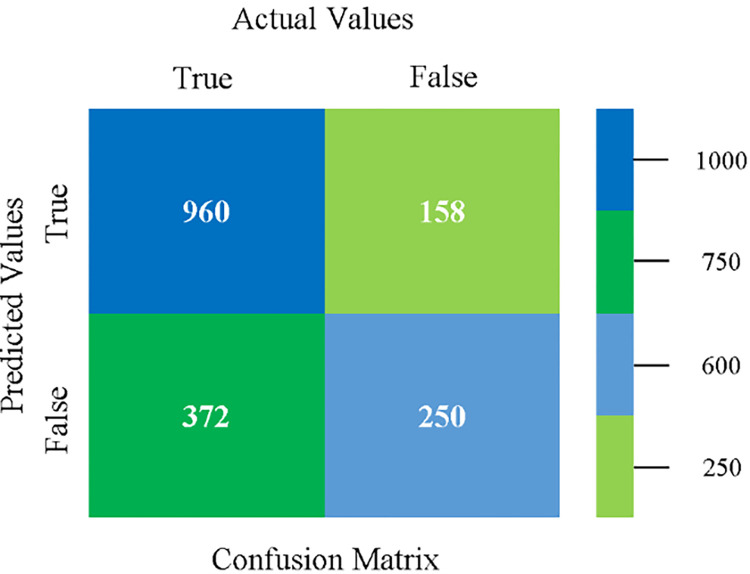
Confusion matrix of RF classifier based on CPU-mem mono in accuracy & fault prediction.

**Fig 19:**
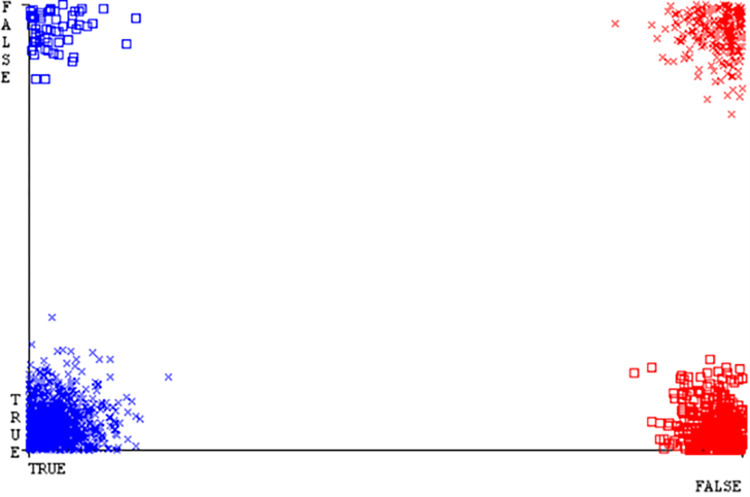
Classifier errors of NB classifier based on CPU-mem mono in accuracy & fault prediction.

**Fig 20:**
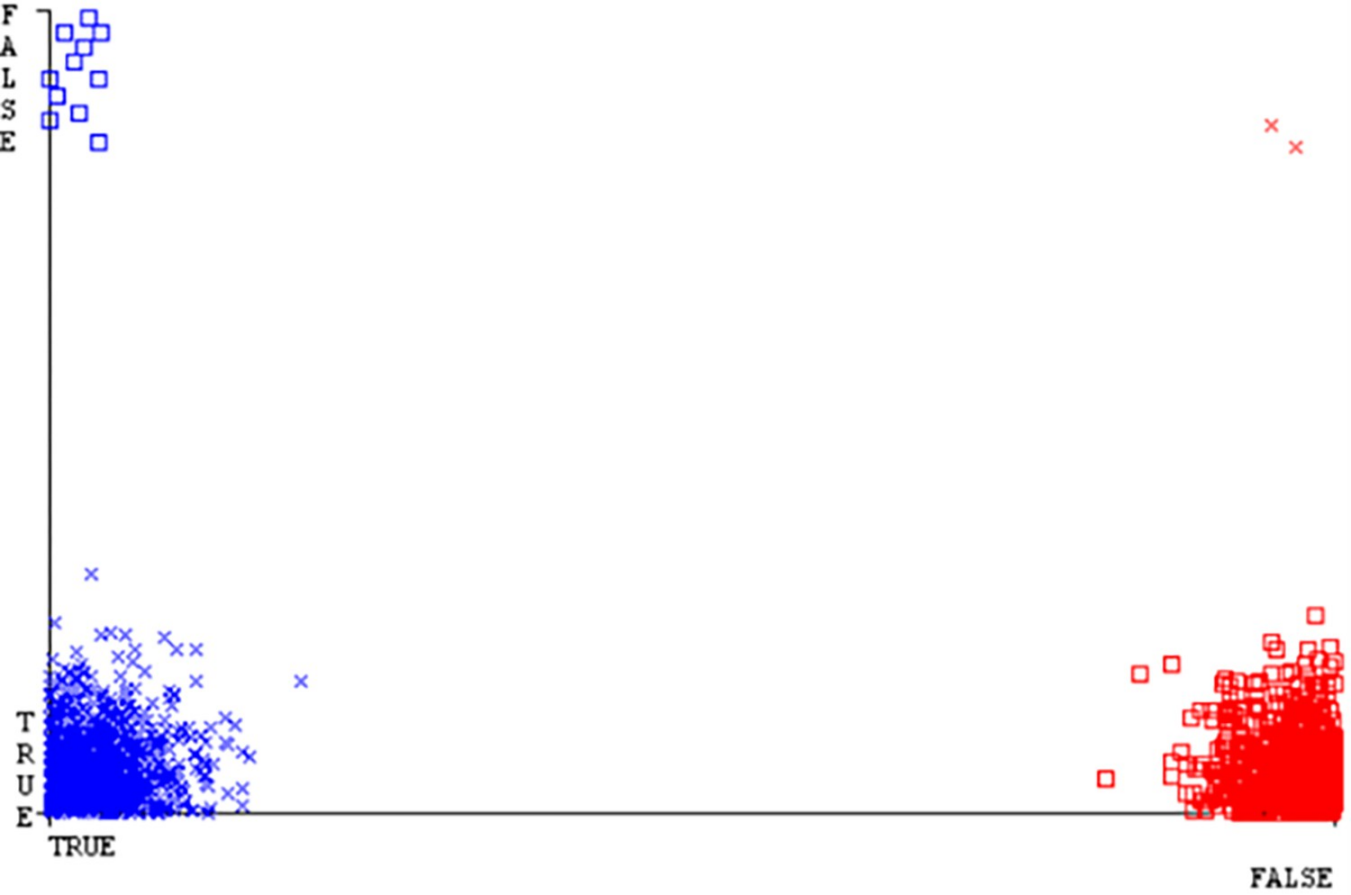
Classifier errors of LIBSVM classifier based on CPU-mem mono in accuracy & fault prediction.

**Fig 21:**
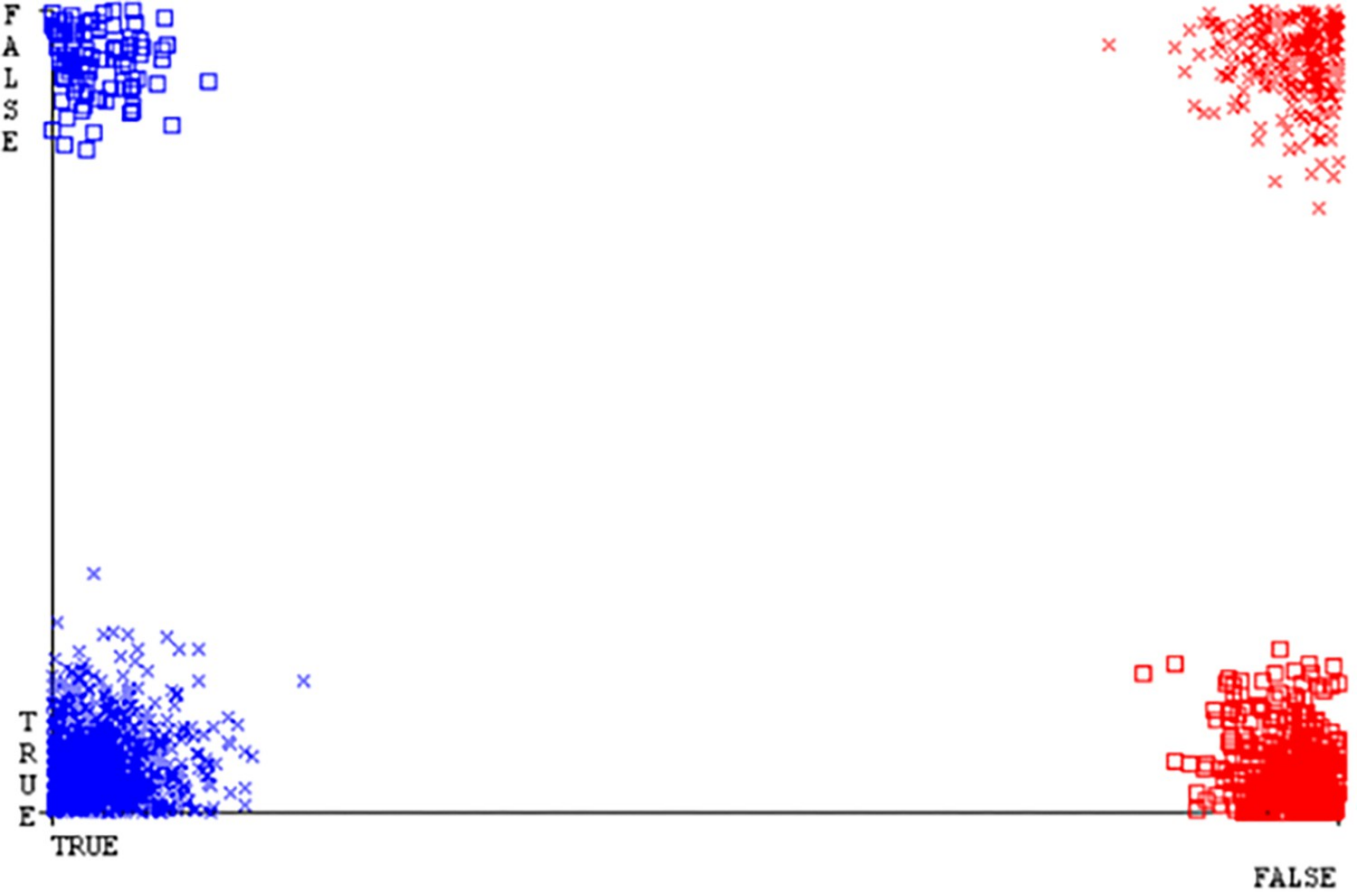
Classifier errors of MLR classifier based on CPU-mem mono in accuracy & fault prediction.

**Fig 22:**
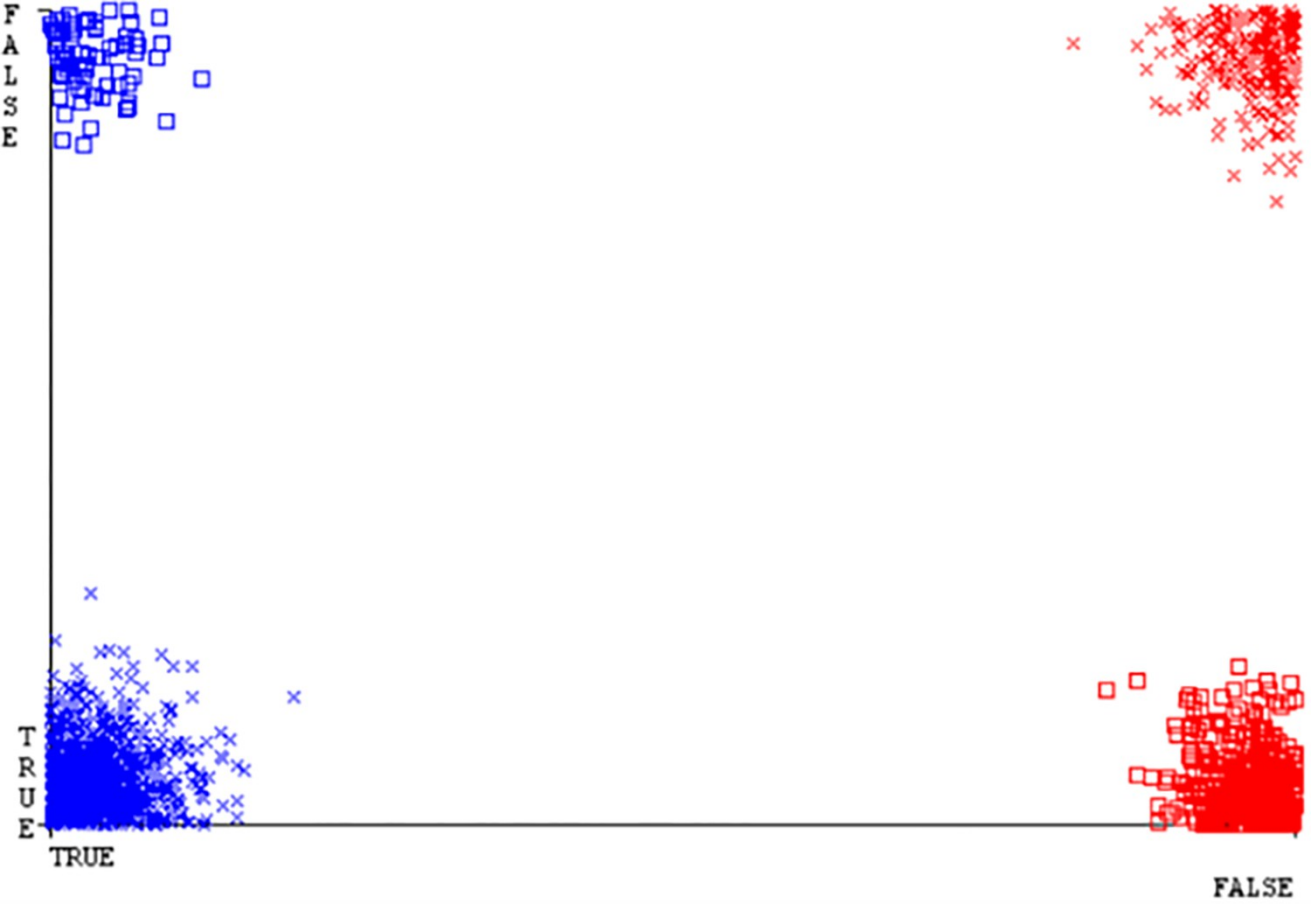
Classifier errors of SMO classifier based on CPU-mem mono in accuracy & fault prediction.

**Fig 23:**
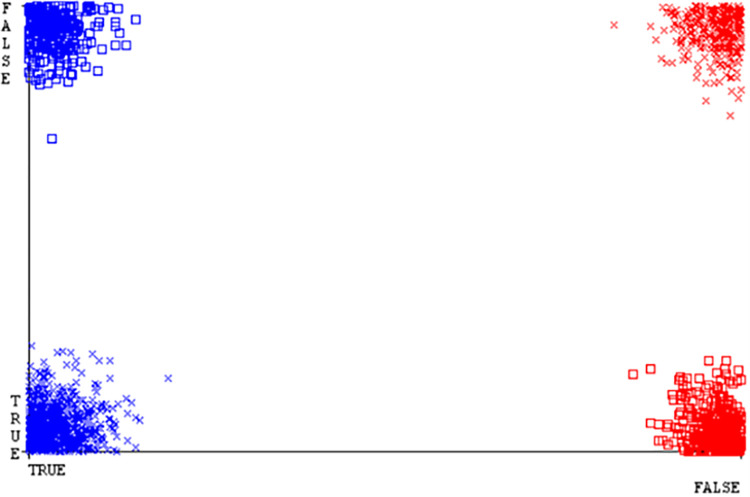
Classifier errors of KNN classifier based on CPU-mem mono in accuracy & fault prediction.

**Fig 24:**
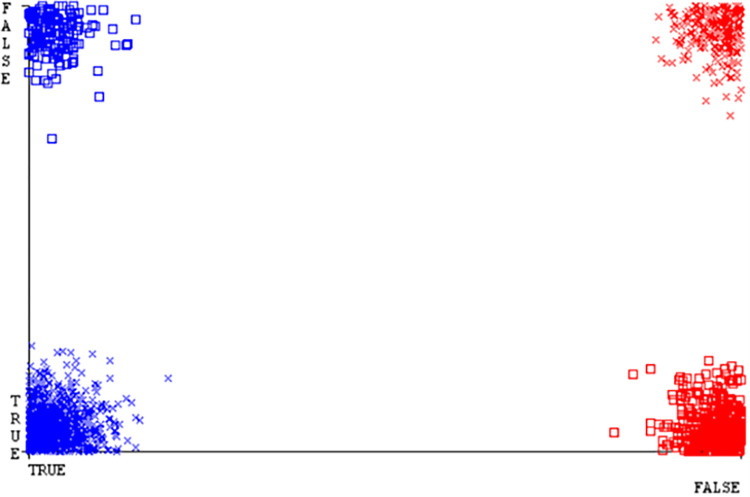
Classifier errors of RF classifier based on CPU-mem mono in accuracy & fault prediction.

**Fig 25:**
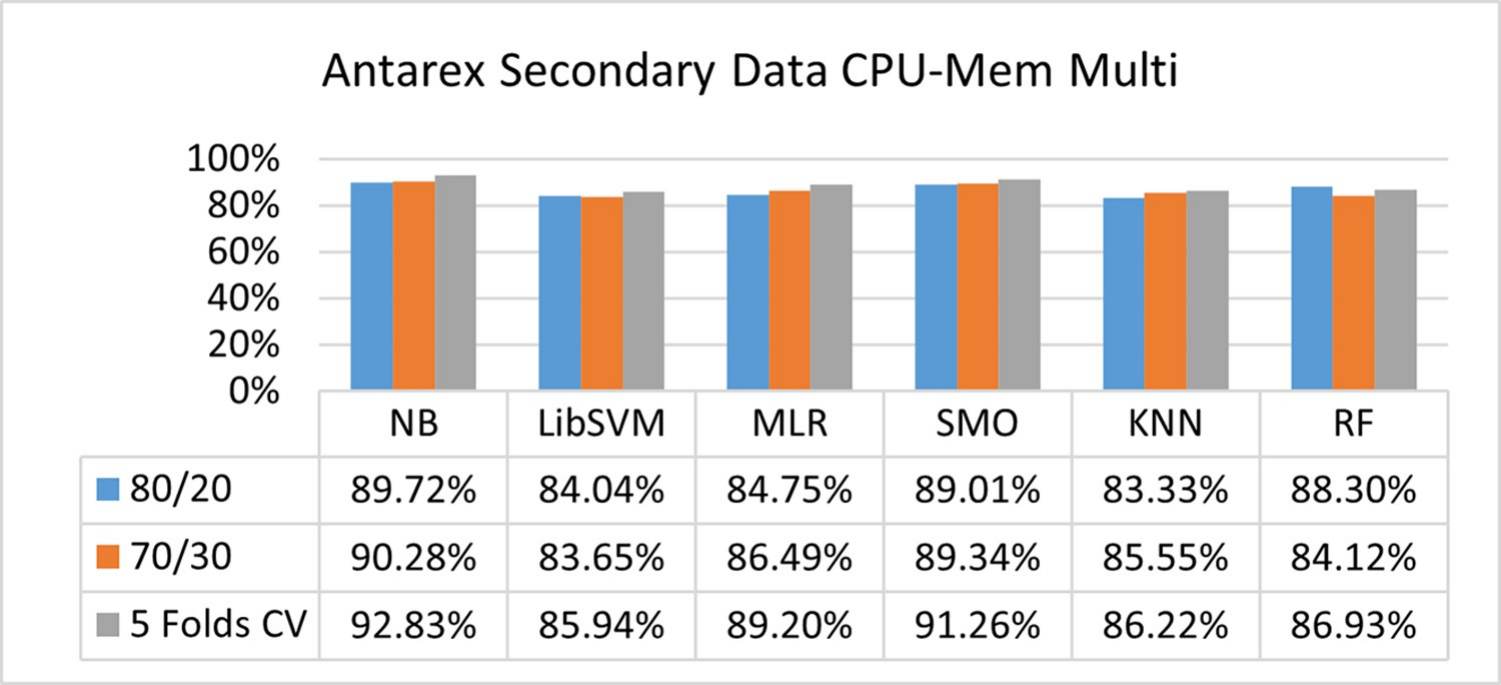
Accuracy by class (true/false) of CPU-mem multi on ML classifiers.

**Fig 26:**
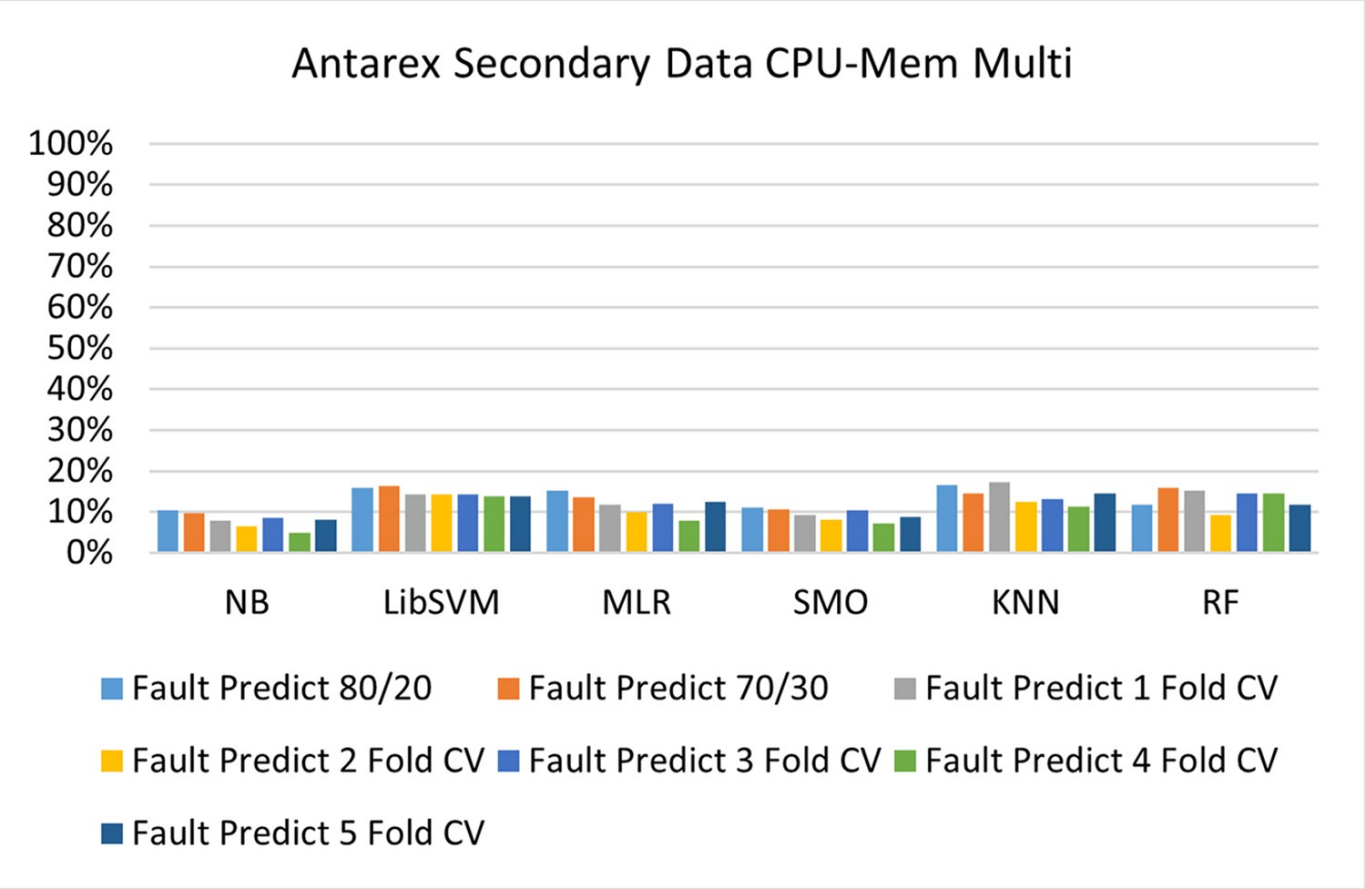
Fault prediction by class (true/false) of CPU-mem multi on ML classifiers.

**Fig 27:**
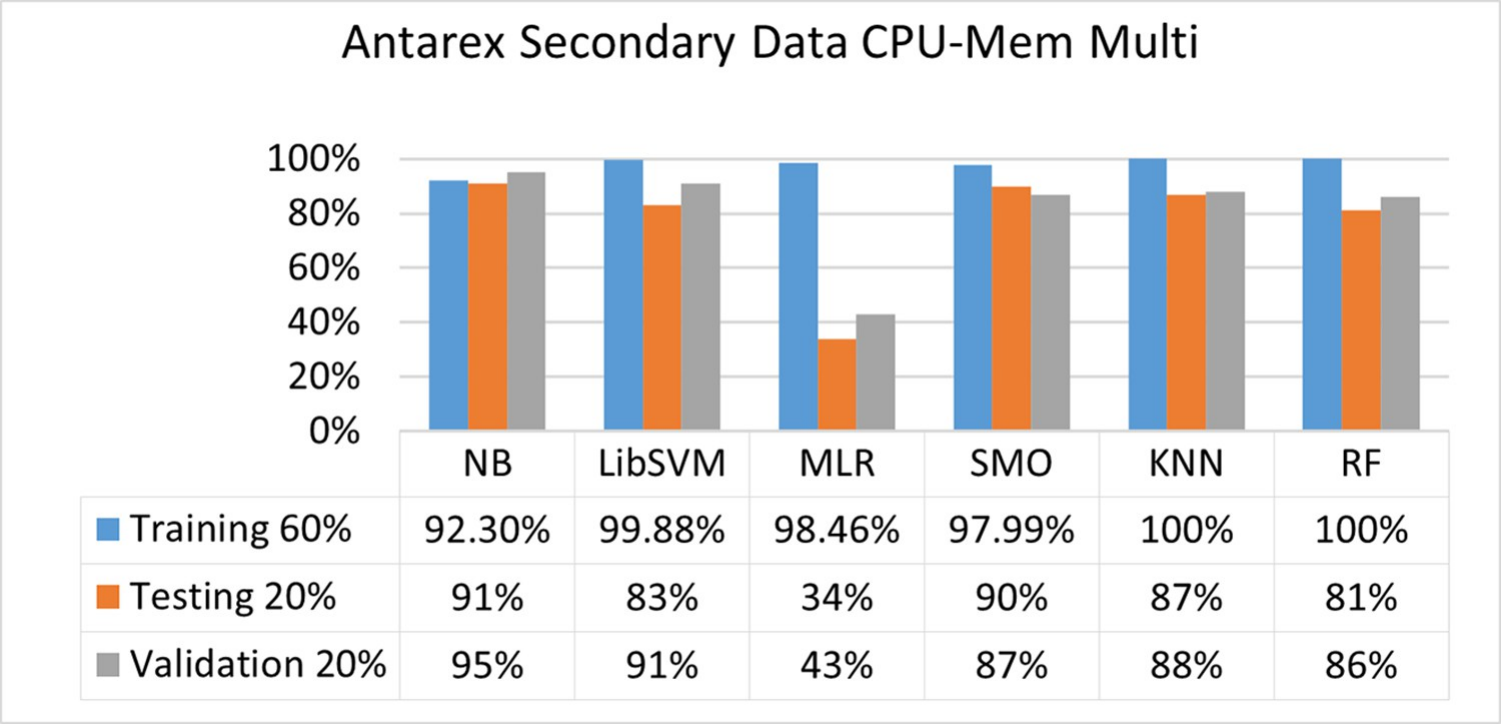
Accuracy by class (true/false) of CPU-mem multi on ML classifiers related to data validation results.

**Fig 28:**
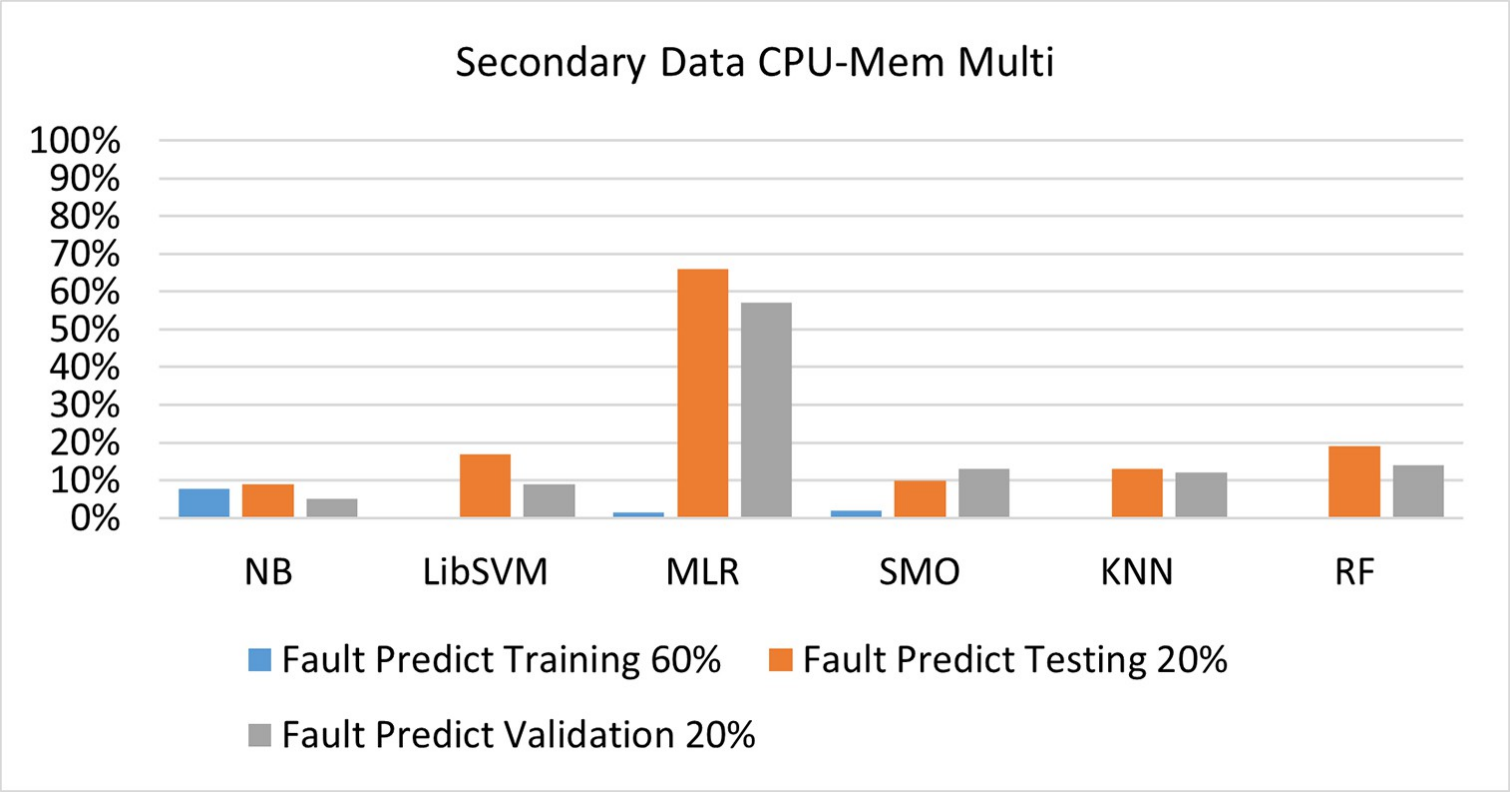
Fault prediction by class (true/false) of CPU-mem multi on ML classifiers related to data validation results.

**Fig 29:**
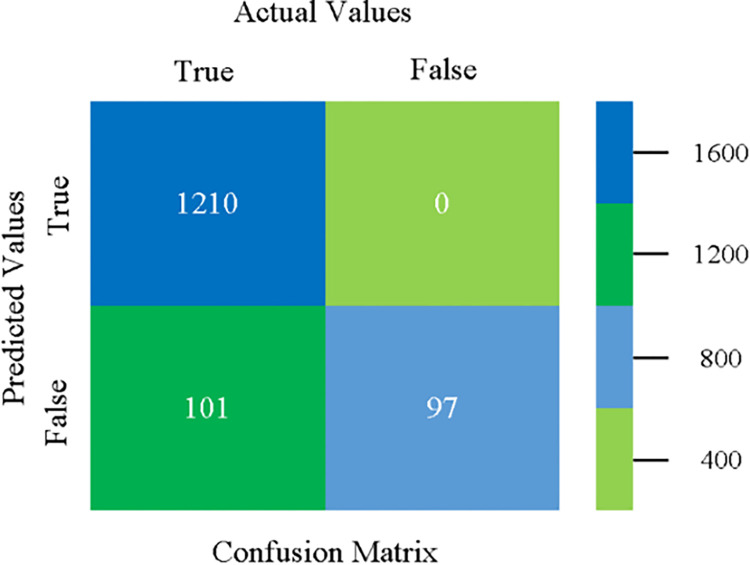
Confusion matrix of NB classifier based on CPU-mem multi in accuracy & fault prediction.

The secondary data and primary data results of each classifier are shown in (Figs 9–72) with the 80/20, 70/30, and 5 folds cross-validation in terms of high accuracy and less fault prediction. Furthermore, data validation of 60% of training, 20% of testing, and 20% of validation. In secondary data results (CPU-Mem Mono) gives the highest percentage of accuracy and less fault prediction on the NB classifier in terms of 80/20 (77.01%), 70/30 (76.05%), and 5 folds cross-validation (74.88%), and (CPU-Mem Multi)) in terms of 80/20 (89.72%), 70/30 (90.28%), and 5 folds cross-validation (92.83%). Furthermore, on (HDD Mono) the SMO classifier gives the highest percentage of accuracy and less fault prediction fault in terms of 80/20 (87.72%), 70/30 (89.41%), and 5 folds cross-validation (88.38%), and (HDD-Multi) in terms of 80/20 (93.64%), 70/30 (90.91%), and 5 folds cross-validation (88.20%).

**Fig 30:**
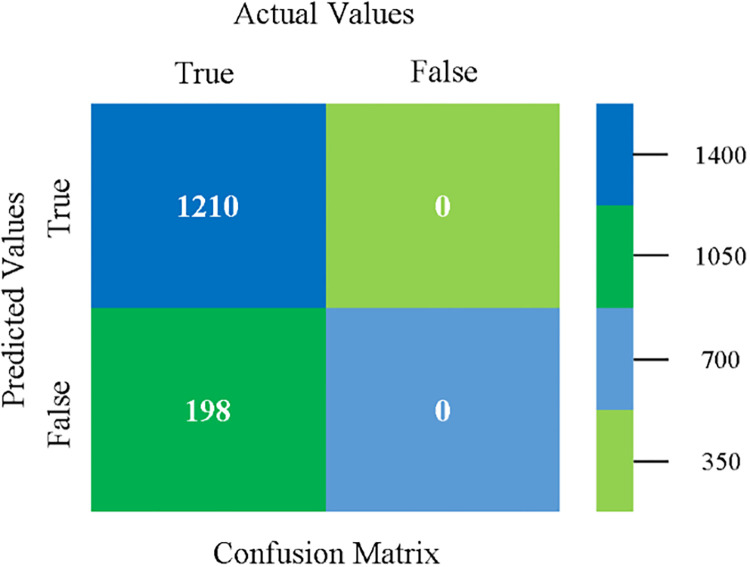
Confusion matrix of LIBSVM classifier based on CPU-mem multi in accuracy & fault prediction.

**Fig 31:**
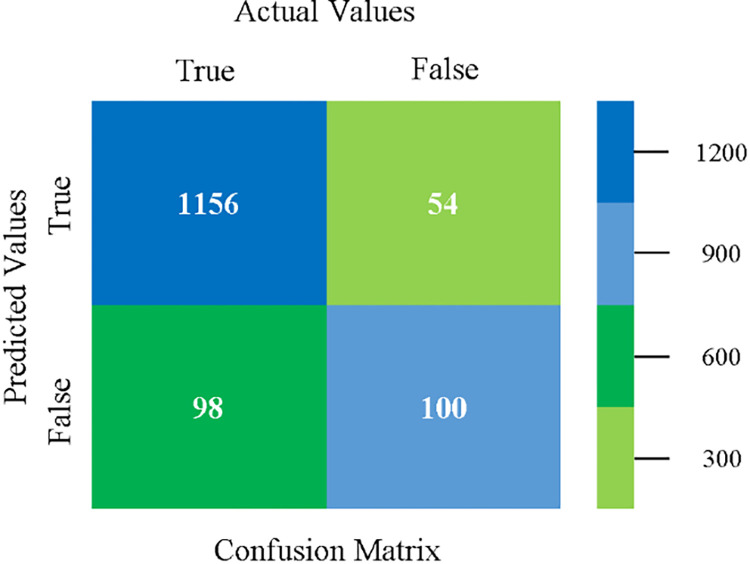
Confusion matrix of MLR classifier based on CPU-mem multi in accuracy & fault prediction.

**Fig 32:**
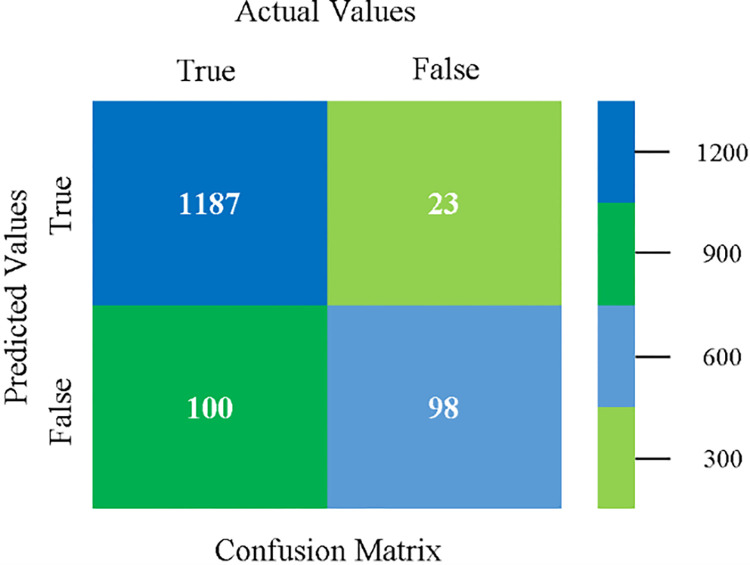
Confusion matrix of SMO classifier based on CPU-mem multi in accuracy & fault prediction.

**Fig 33:**
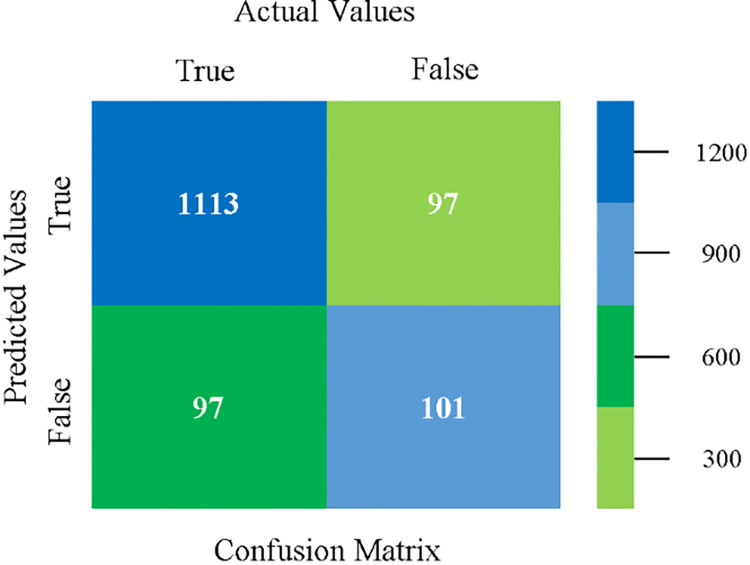
Confusion matrix of KNN classifier based on CPU-mem multi in accuracy & fault prediction.

**Fig 34:**
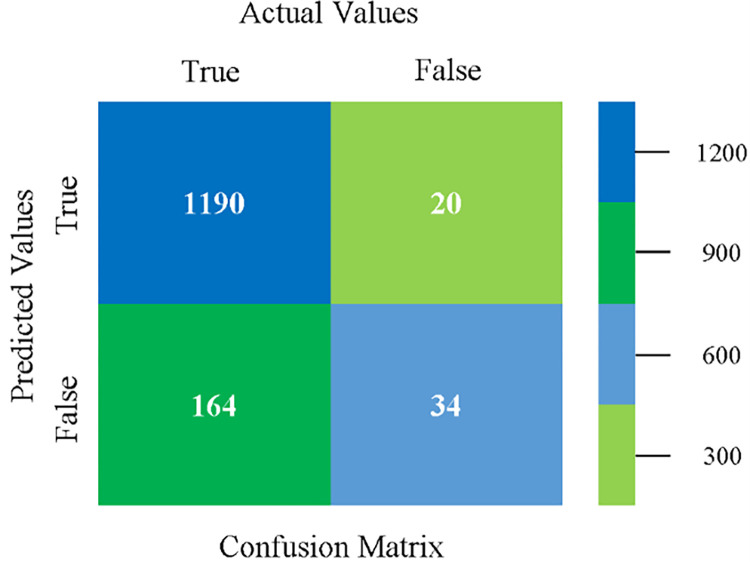
Confusion matrix of RF classifier based on CPU-mem multi in accuracy & fault prediction.

**Fig 35:**
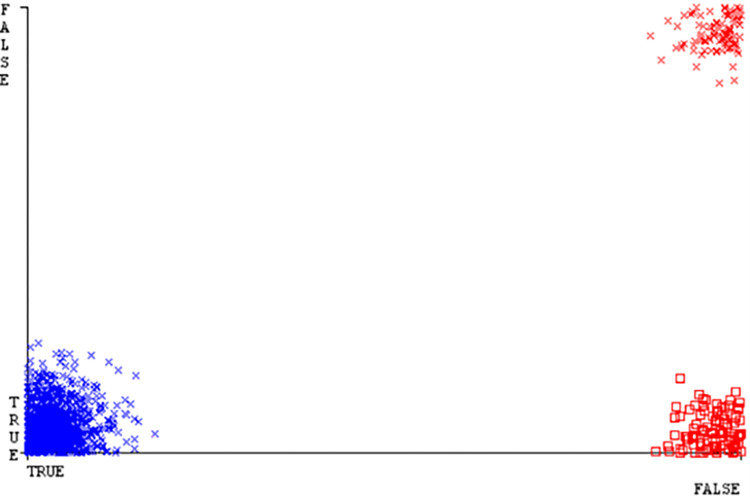
Classifier errors of NB classifier based on CPU-mem multi in accuracy & fault prediction.

**Fig 36:**
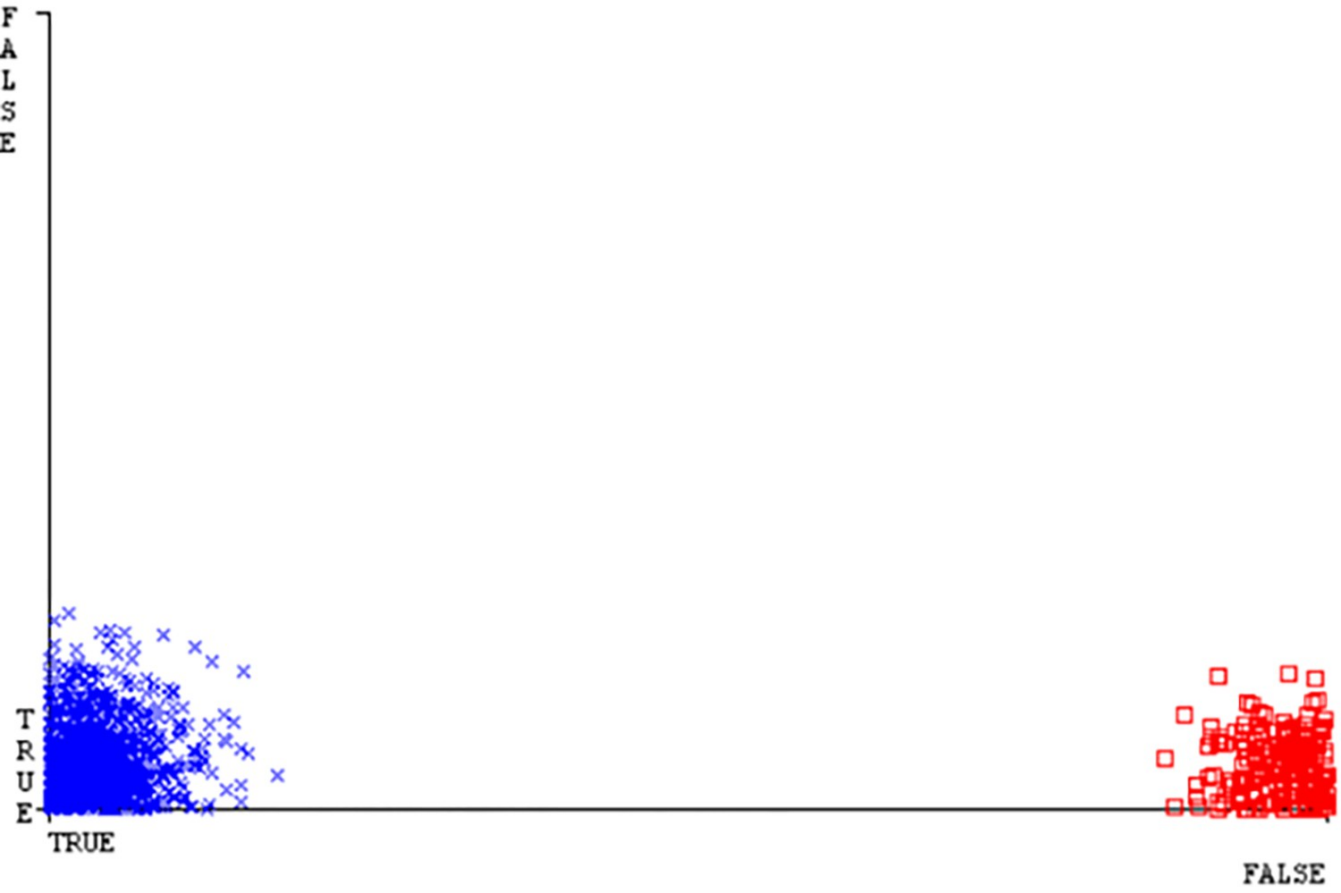
Classifier errors of LIBSVM classifier based on CPU-mem multi in accuracy & fault prediction.

**Fig 37:**
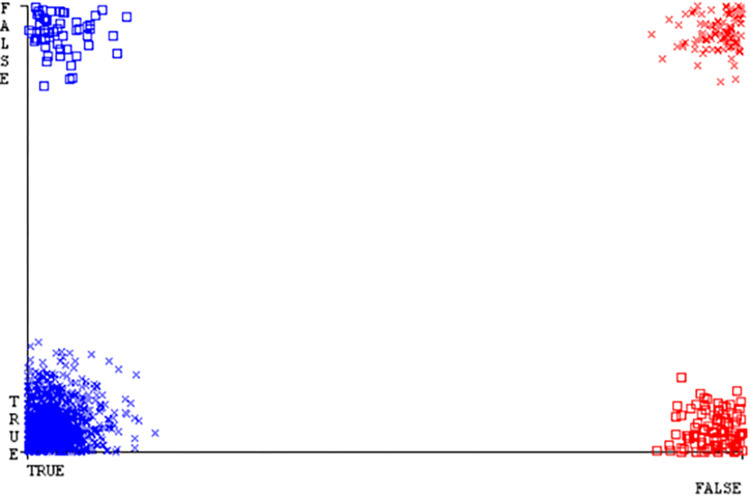
Classifier errors of MLR classifier based on CPU-mem multi in accuracy & fault prediction.

**Fig 38:**
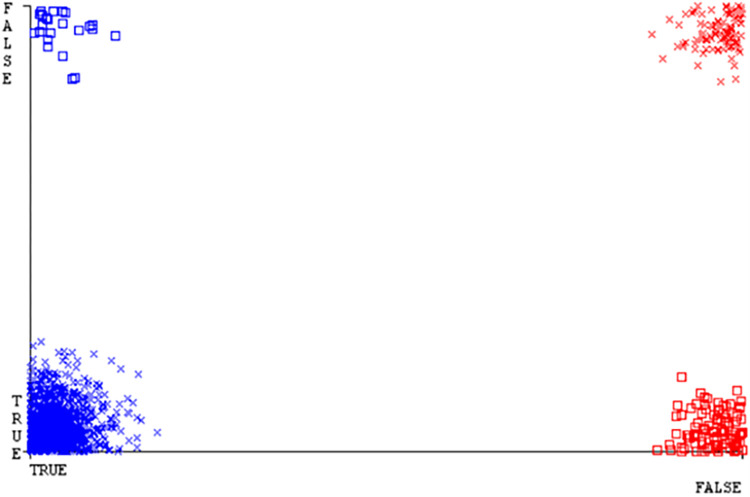
Classifier errors of SMO classifier based on CPU-mem multi in accuracy & fault prediction.

**Fig 39:**
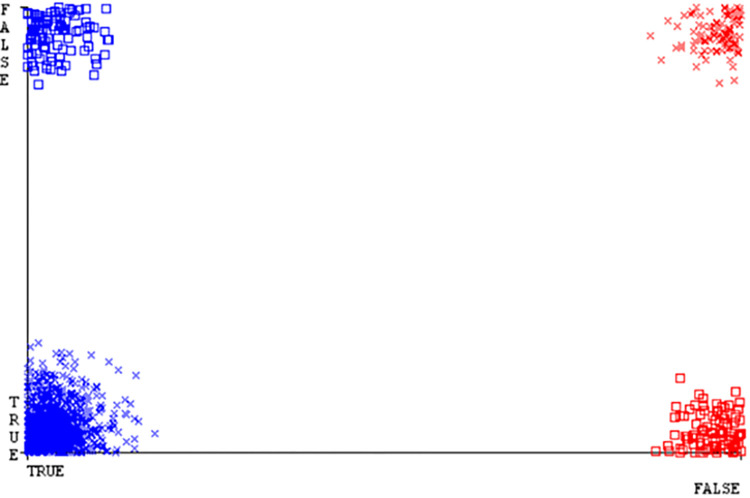
Classifier errors of KNN classifier based on CPU-mem multi in accuracy & fault prediction.

**Fig 40:**
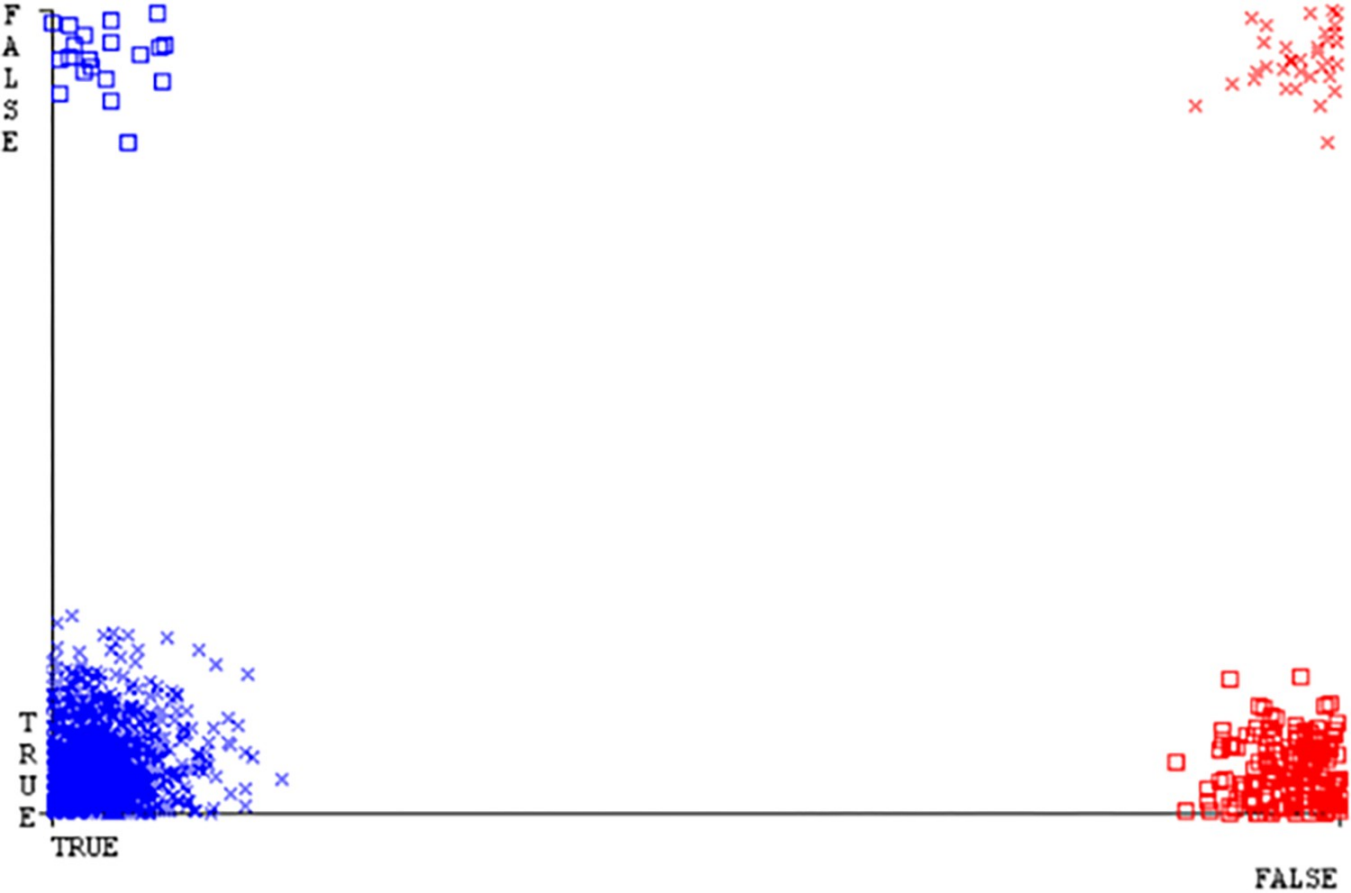
Classifier errors of RF classifier based on CPU-mem multi in accuracy & fault prediction.

**Fig 41:**
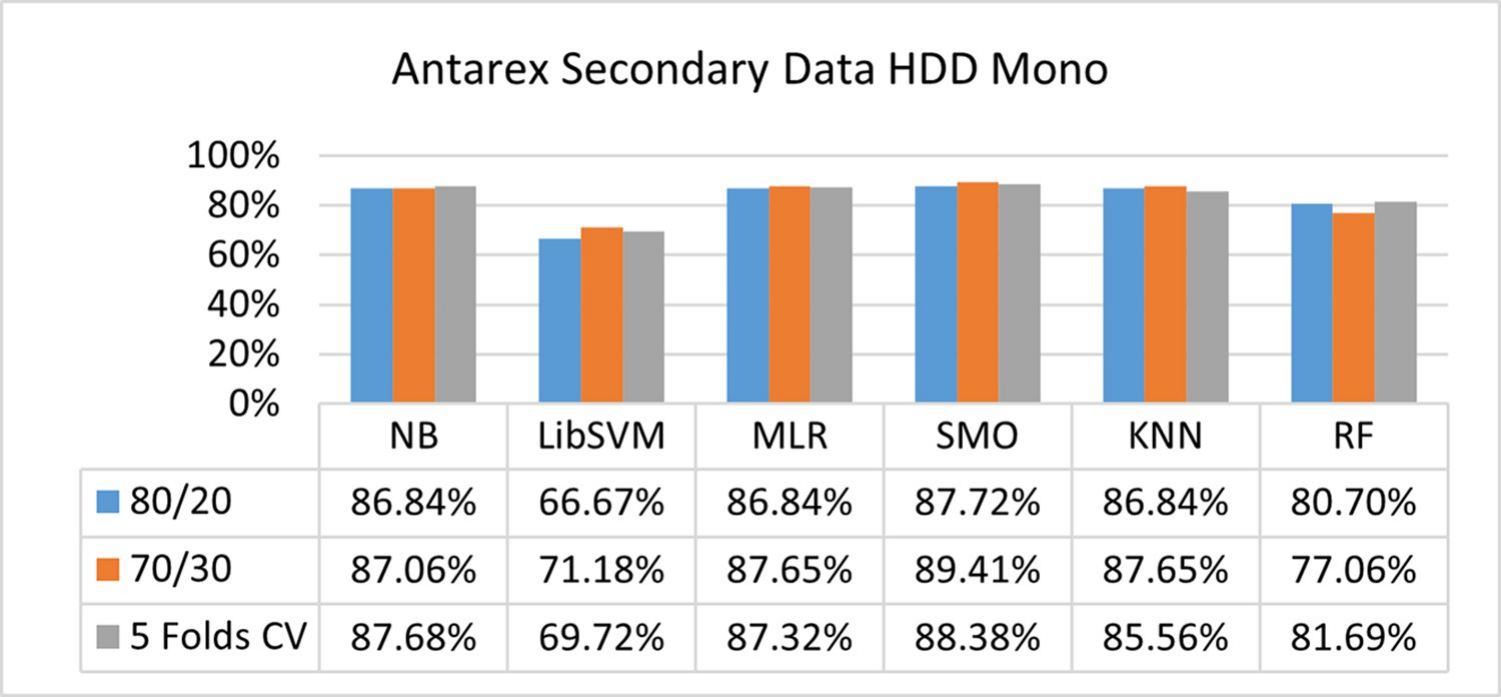
Accuracy by class (true/false) of HDD mono on ML classifiers.

**Fig 42:**
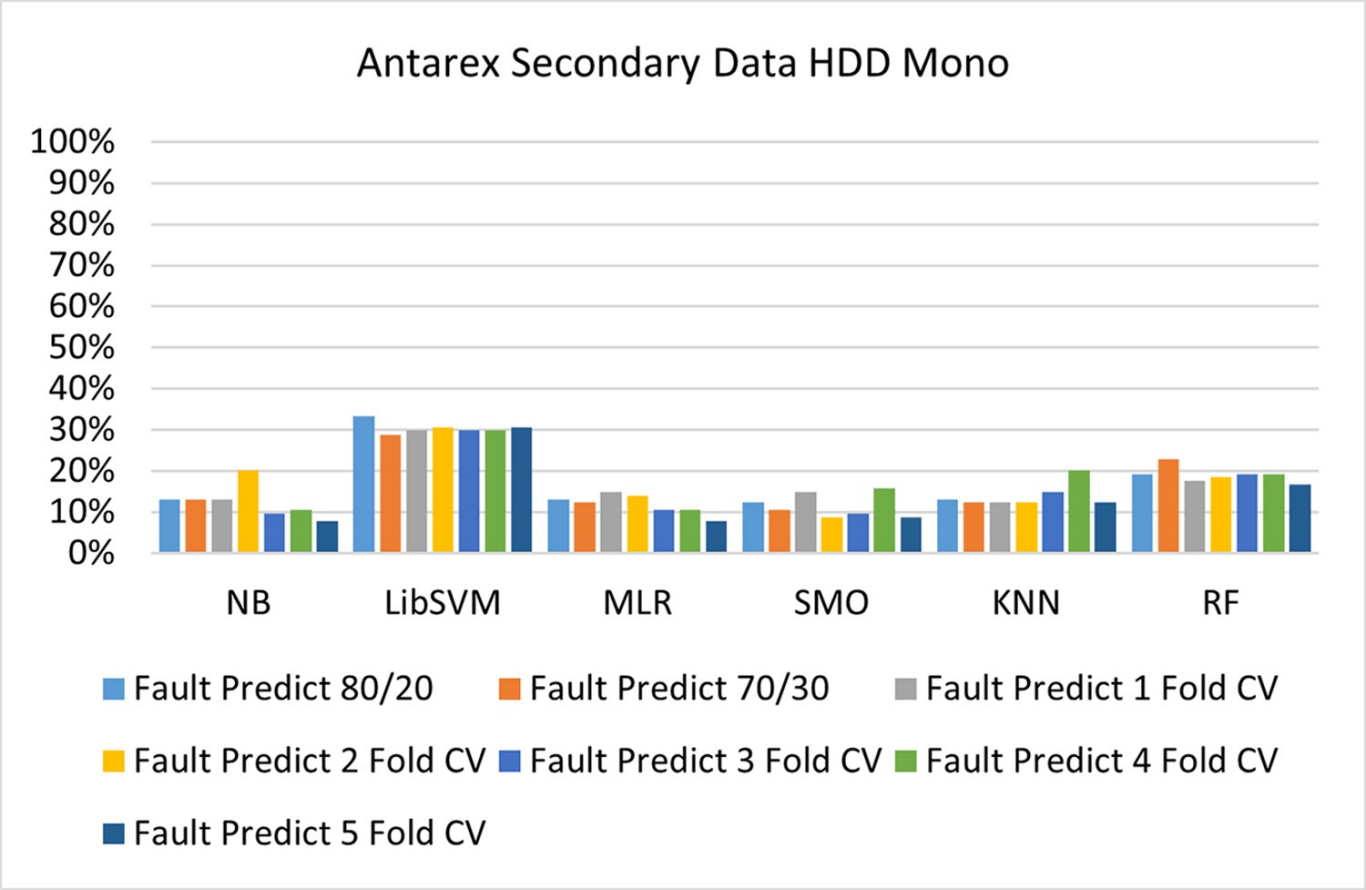
Fault prediction by class (true/false) of HDD mono on ML classifiers.

**Fig 43:**
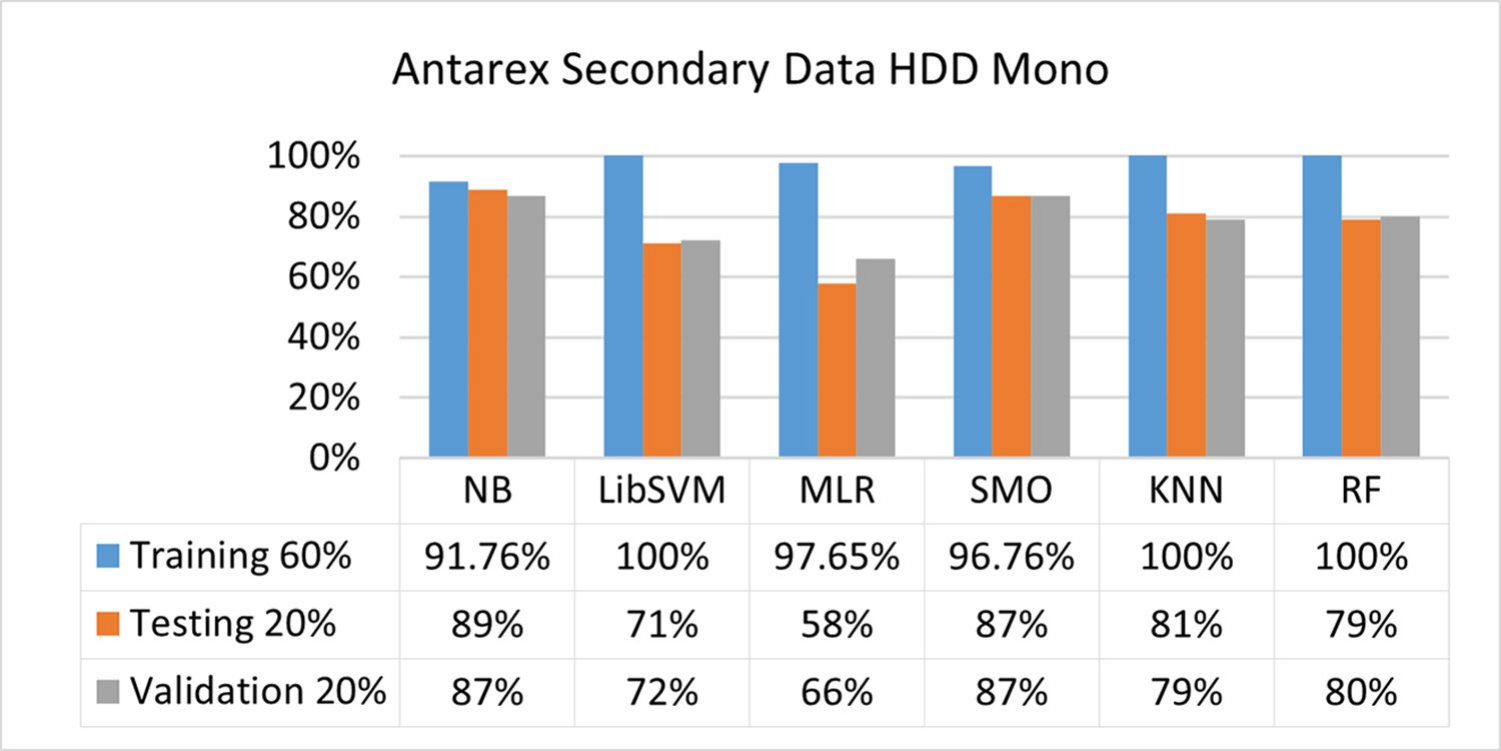
Accuracy by class (true/false) of HDD mono on ML classifiers related to data validation results.

**Fig 44:**
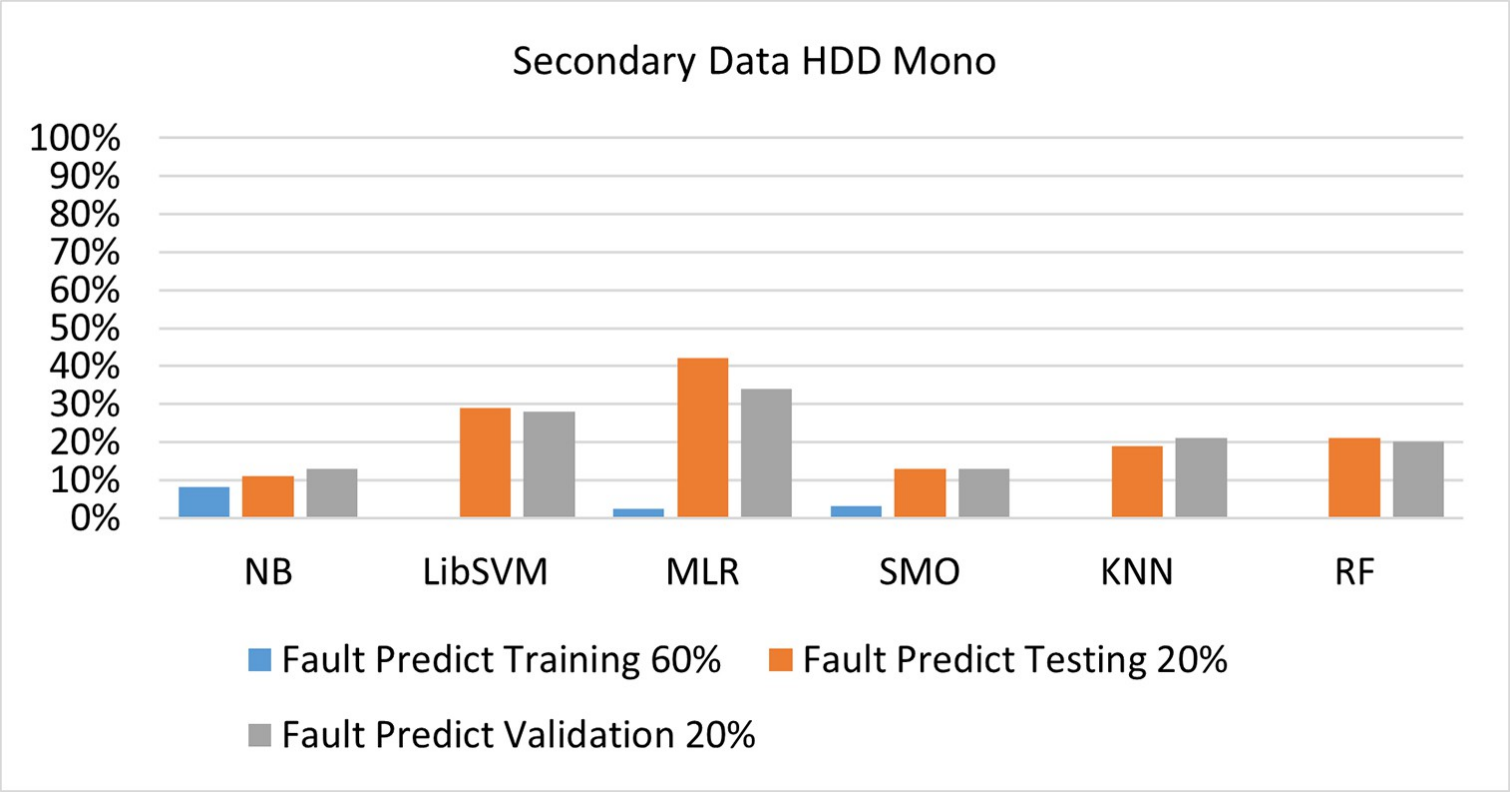
Fault Prediction by class (true/false) of HDD mono on ML classifiers related to data validation results.

**Fig 45:**
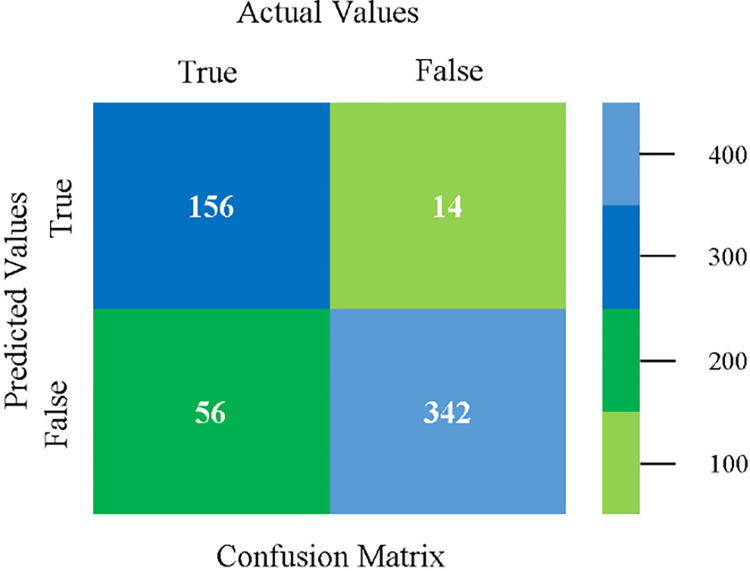
Confusion matrix of NB classifier based on HDD mono in accuracy & fault prediction.

**Fig 46:**
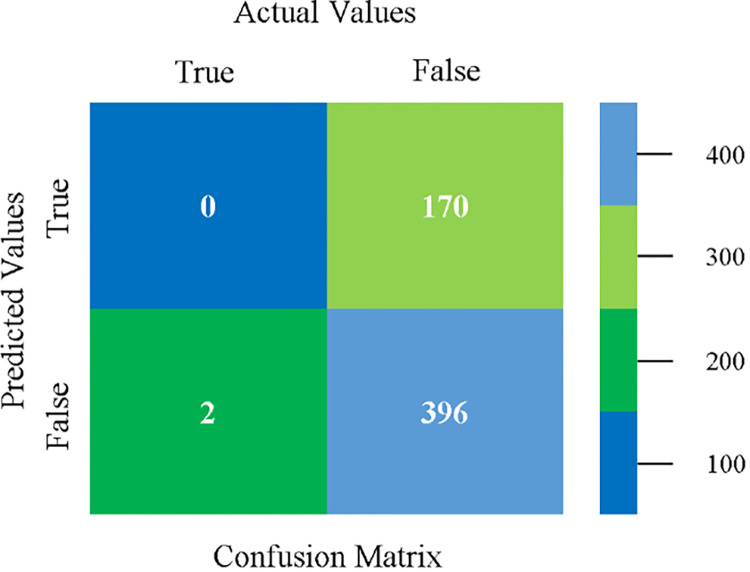
Confusion matrix of LIBSVM classifier based on HDD mono in accuracy & fault prediction.

**Fig 47:**
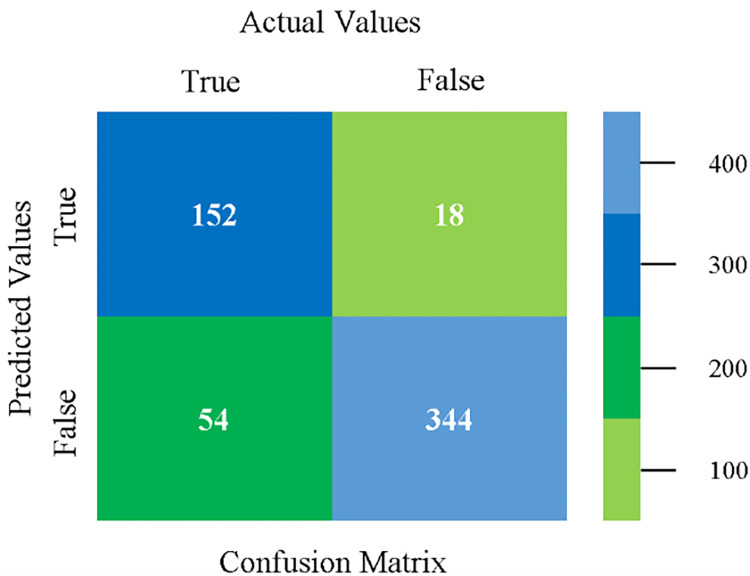
Confusion matrix of MLR classifier based on HDD mono in accuracy & fault prediction.

**Fig 48:**
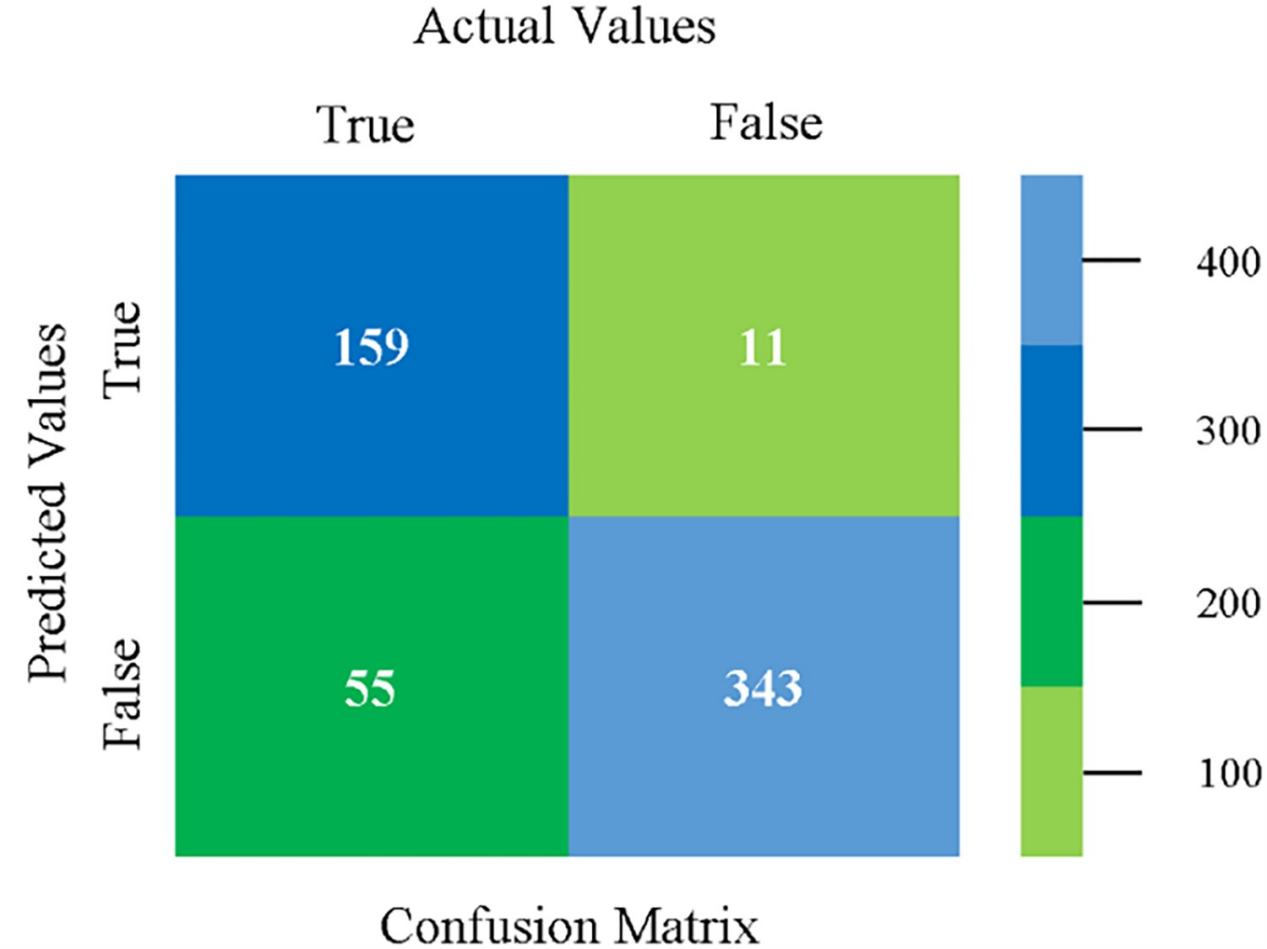
Confusion matrix of SMO classifier based on HDD mono in accuracy & fault prediction.

**Fig 49:**
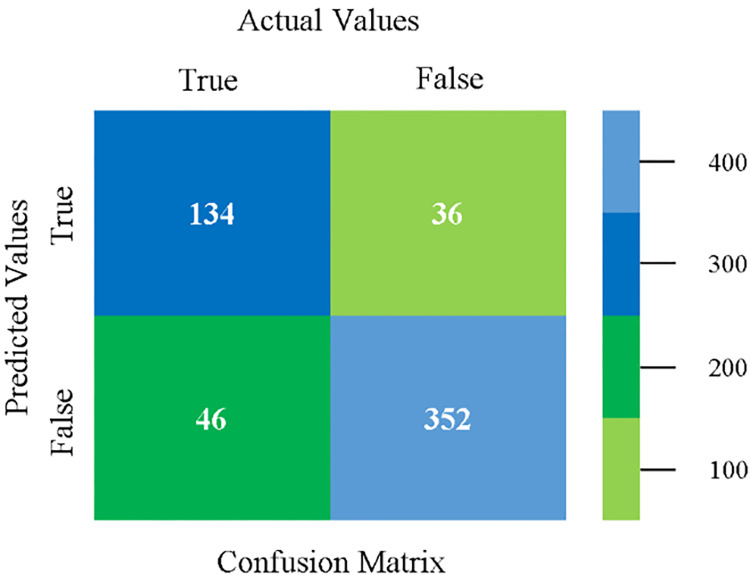
Confusion matrix of KNN classifier based on HDD mono in accuracy & fault prediction.

**Fig 50:**
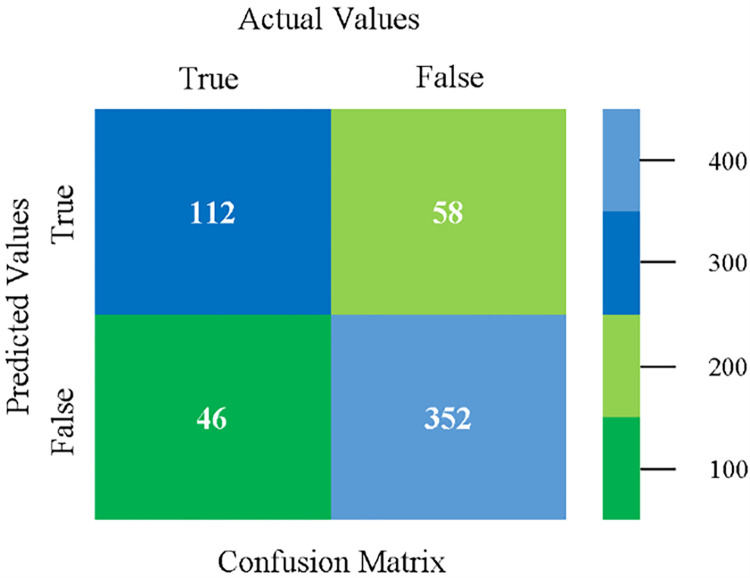
Confusion matrix of RF classifier based on HDD mono in accuracy & fault prediction.

**Fig 51:**
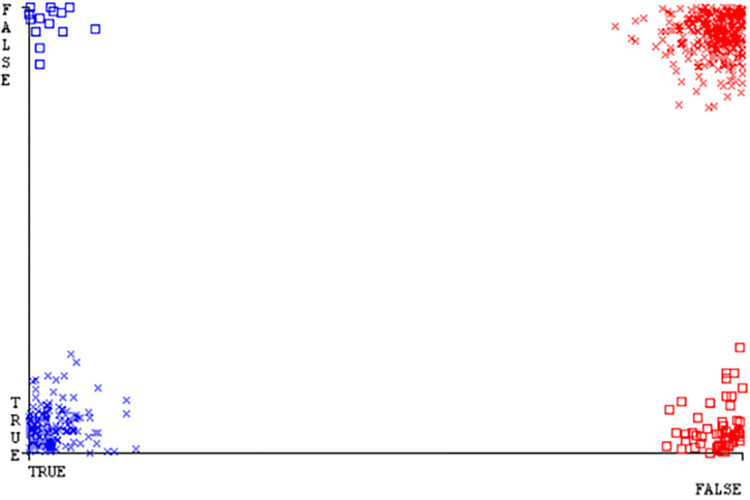
Classifier errors of NB classifier based on HDD mono in accuracy & fault prediction.

**Fig 52:**
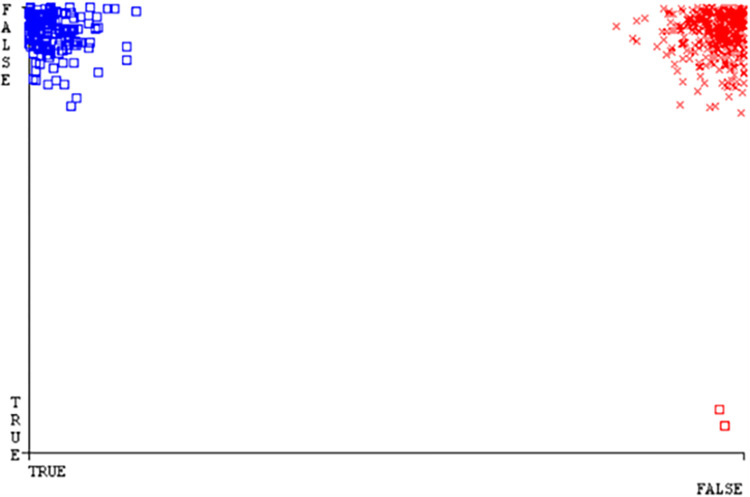
Classifier errors of LIBSVM classifier based on HDD mono in accuracy & fault prediction.

**Fig 53:**
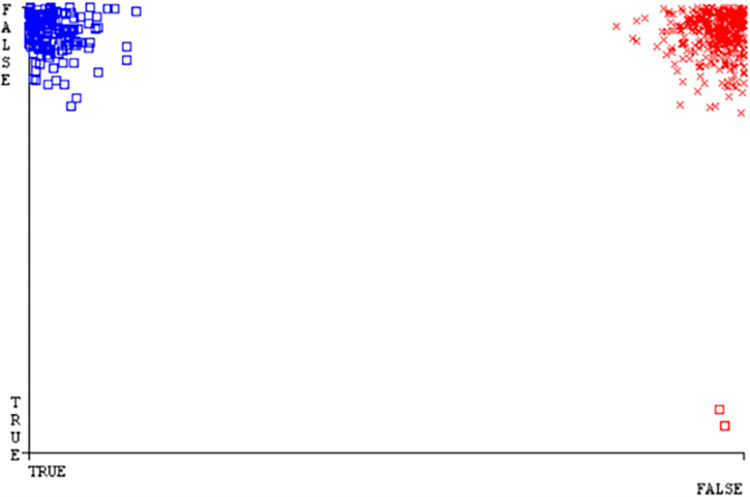
Classifier errors of MLR classifier based on HDD mono in accuracy & fault prediction.

**Fig 54:**
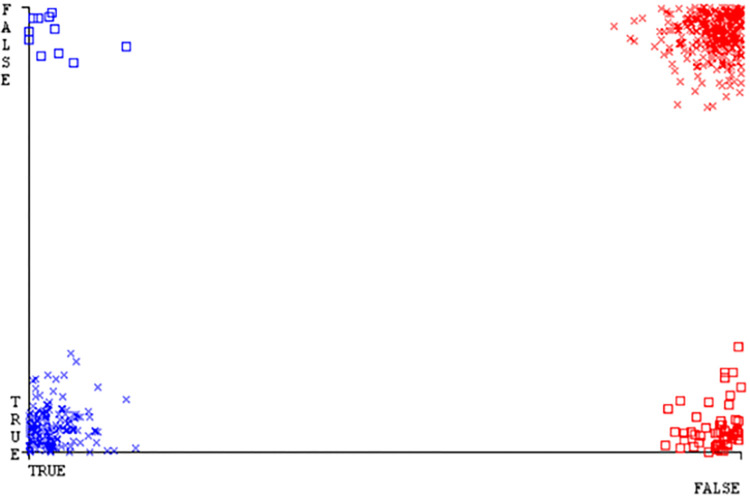
Classifier errors of SMO classifier based on HDD mono in accuracy & fault prediction.

**Fig 55:**
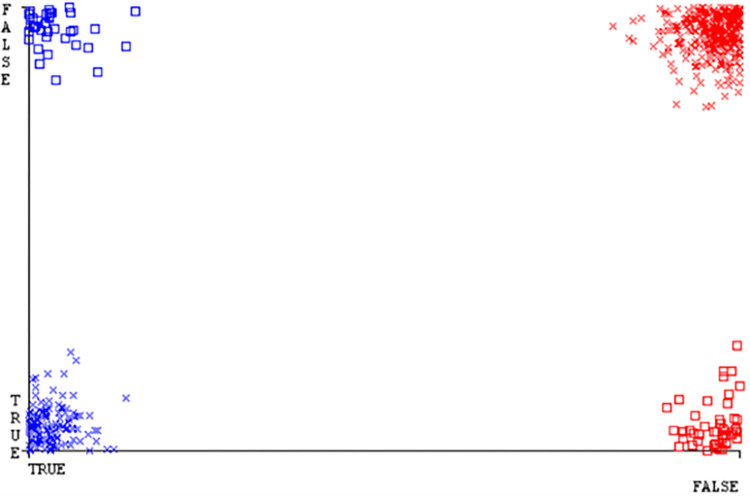
Classifier errors of KNN classifier based on HDD mono in accuracy & fault prediction.

**Fig 56:**
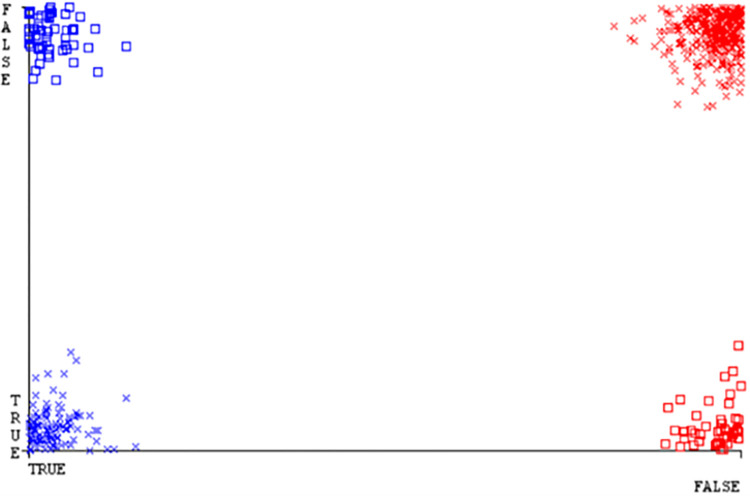
Classifier errors of RF classifier based on HDD mono in accuracy & fault prediction.

**Fig 57:**
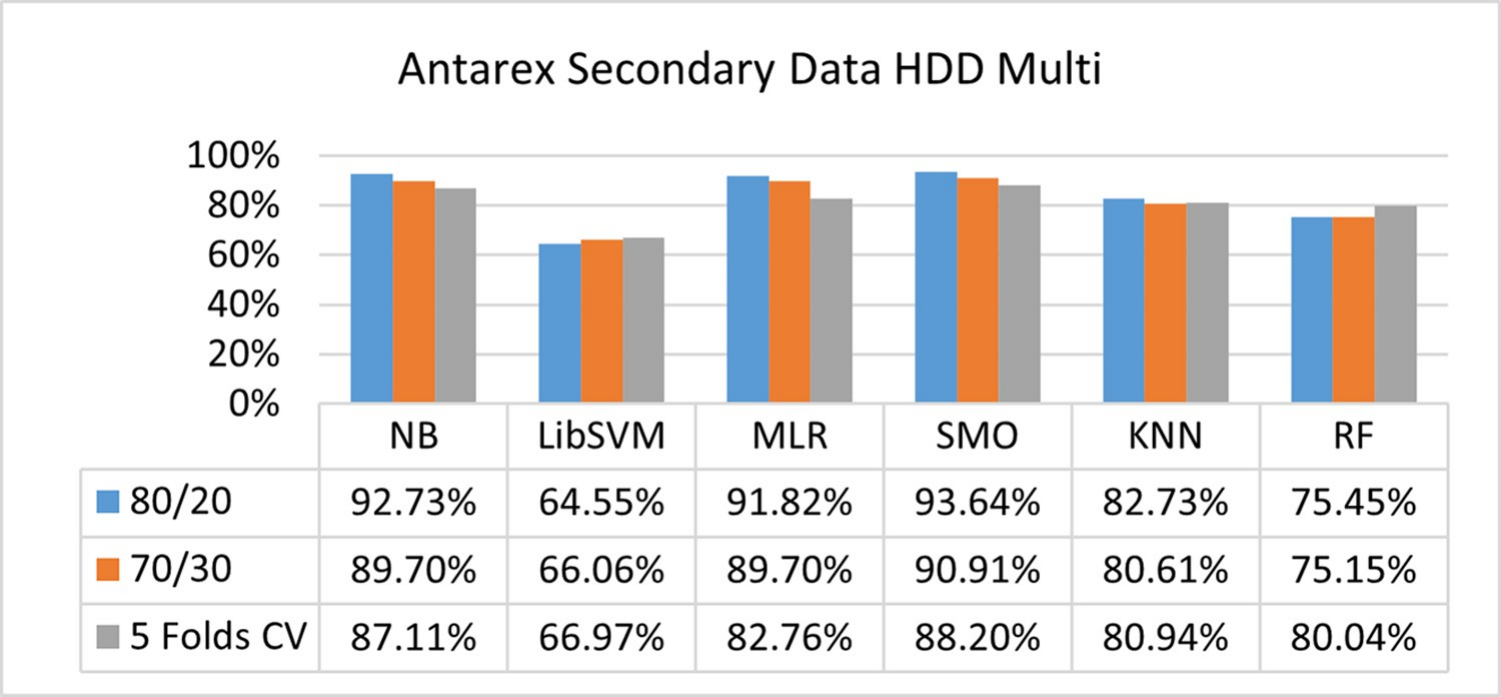
Accuracy by class (true/false) of HDD multi on ML classifiers.

**Fig 58:**
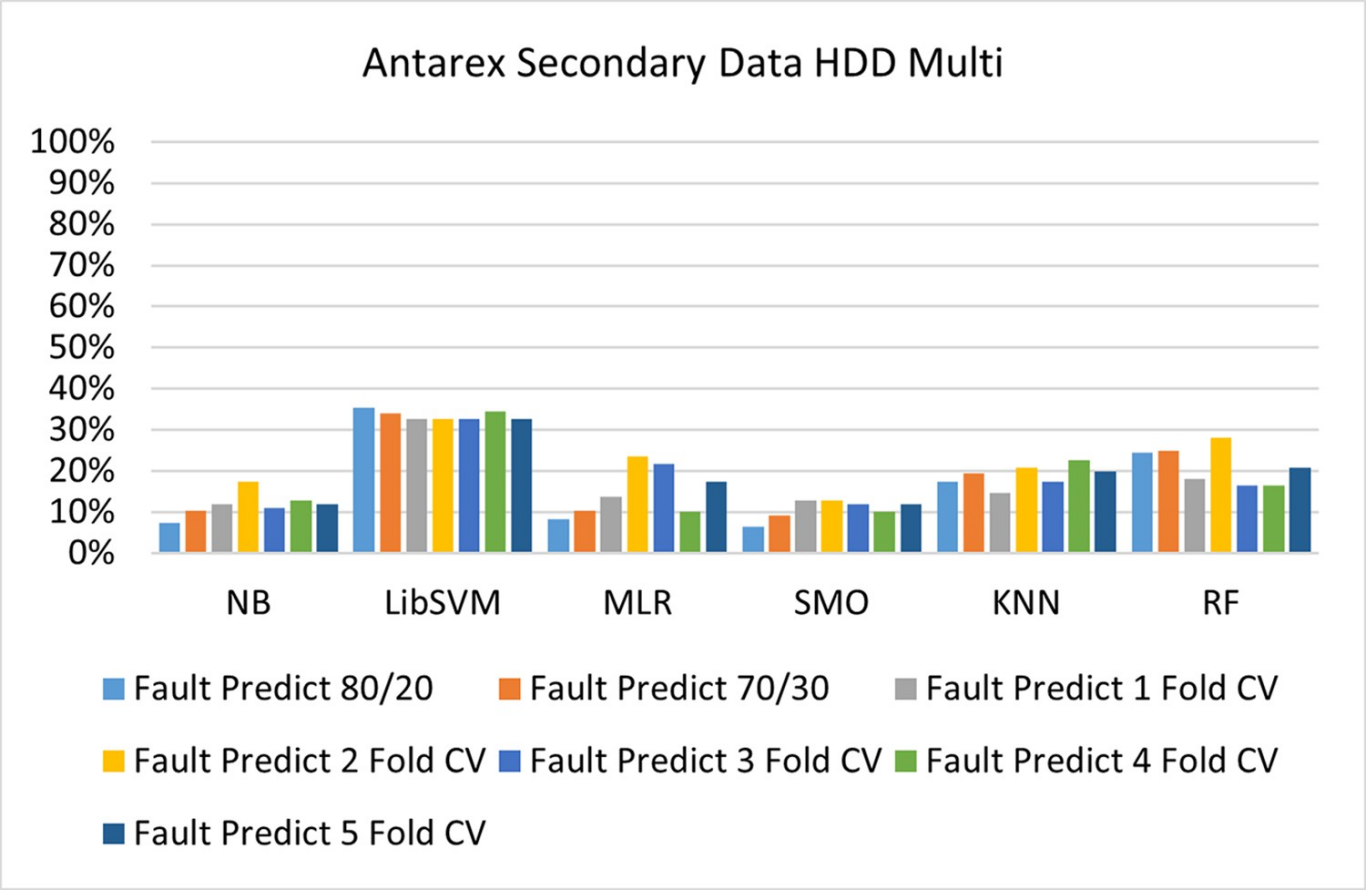
Fault prediction by class (true/false) of HDD multi on ML classifiers.

**Fig 59:**
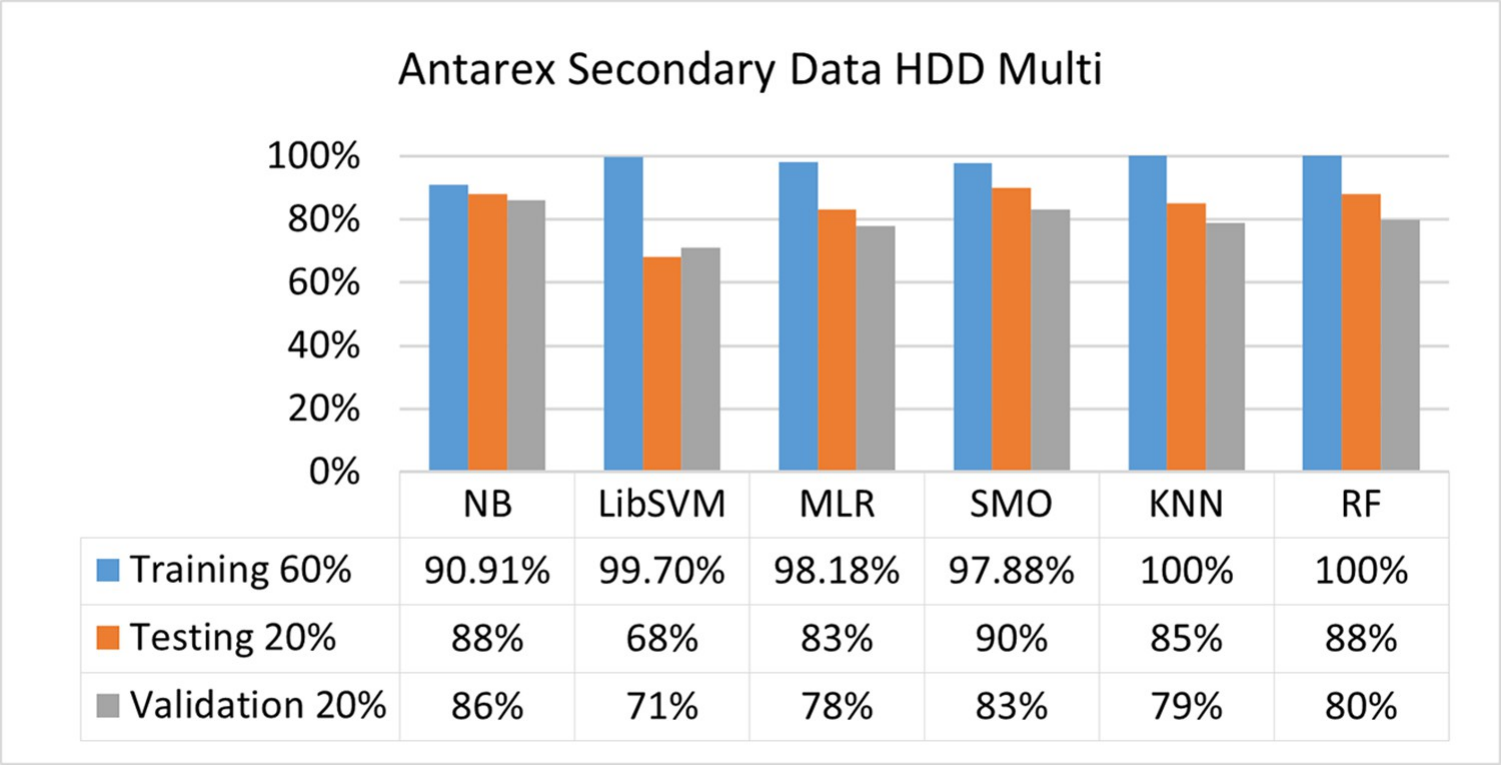
Accuracy by class (true/false) of HDD multi-on ML classifiers related to data validation results.

**Fig 60:**
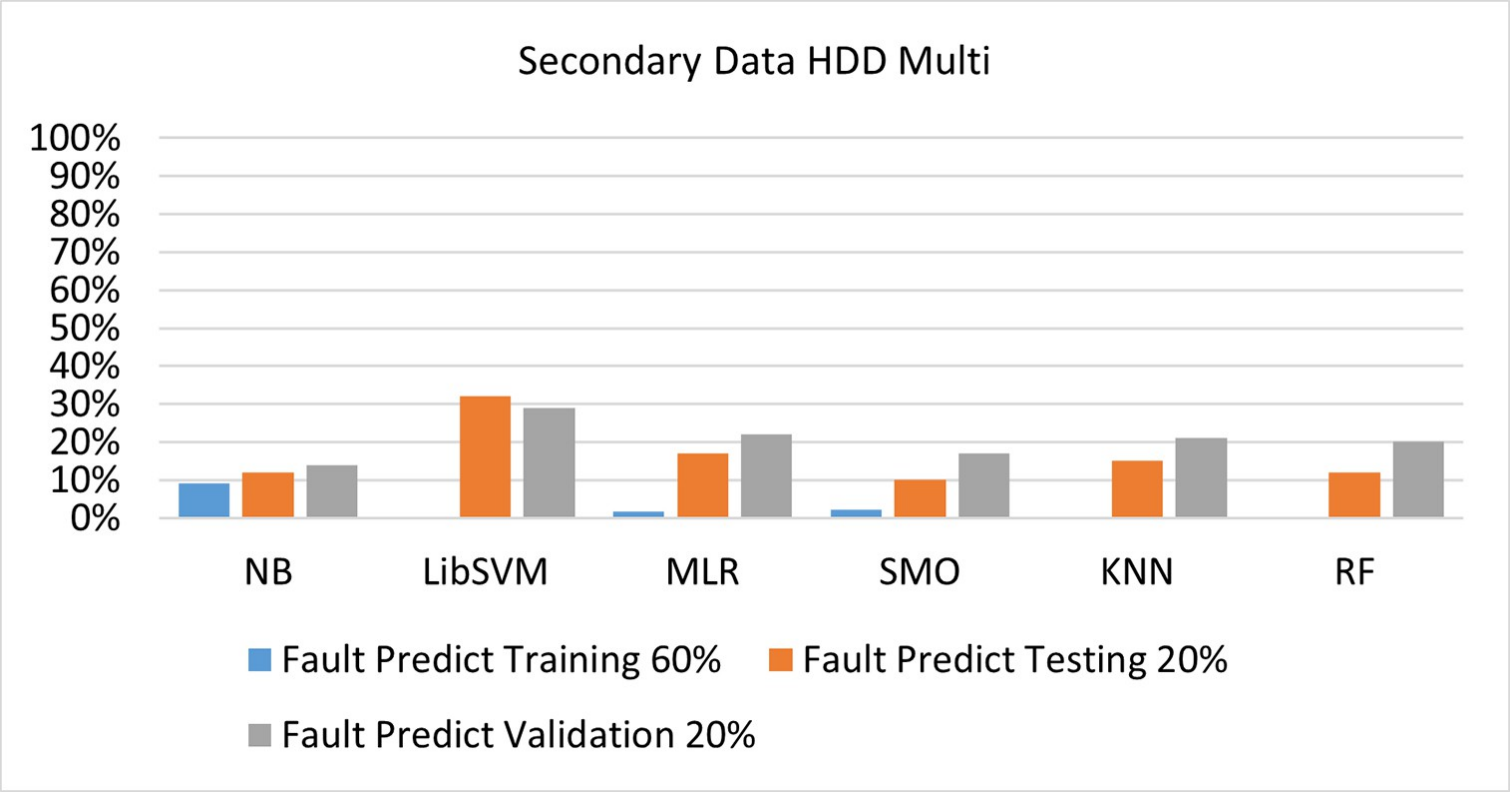
Fault prediction by class (true/false) of HDD multi-on ML classifiers related to data validation results.

**Fig 61:**
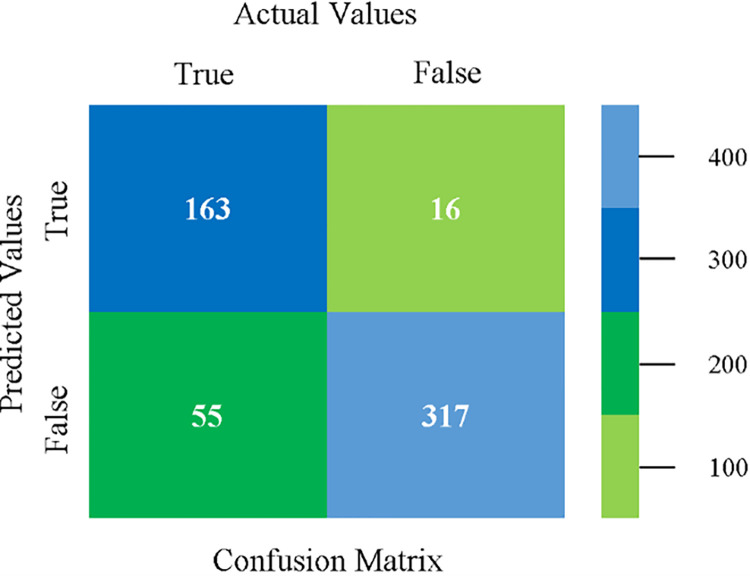
Confusion matrix of NB classifier based on HDD multi in accuracy & fault prediction.

**Fig 62:**
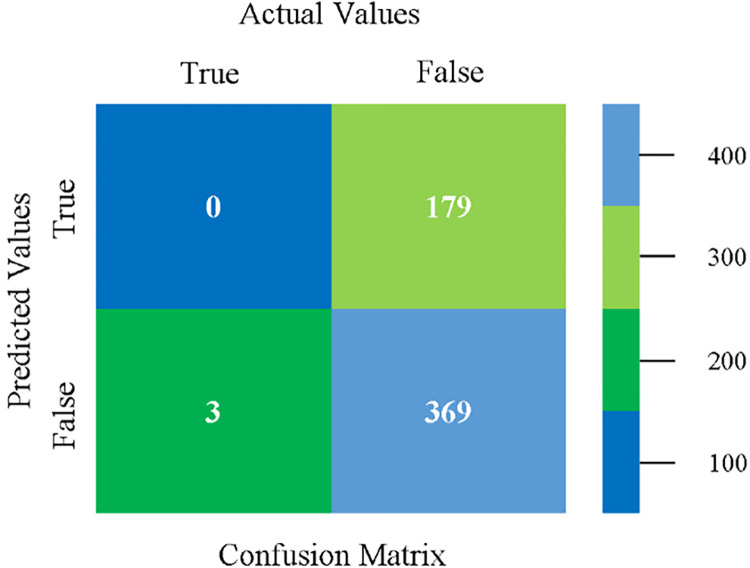
Confusion matrix of LIBSVM classifier based on HDD multi in accuracy & fault prediction.

**Fig 63:**
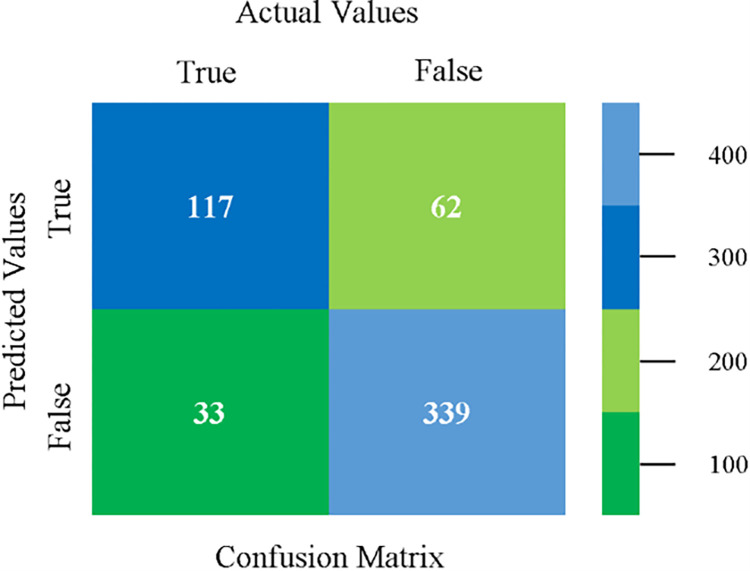
Confusion matrix of MLR classifier based on HDD multi in accuracy & fault prediction.

**Fig 64:**
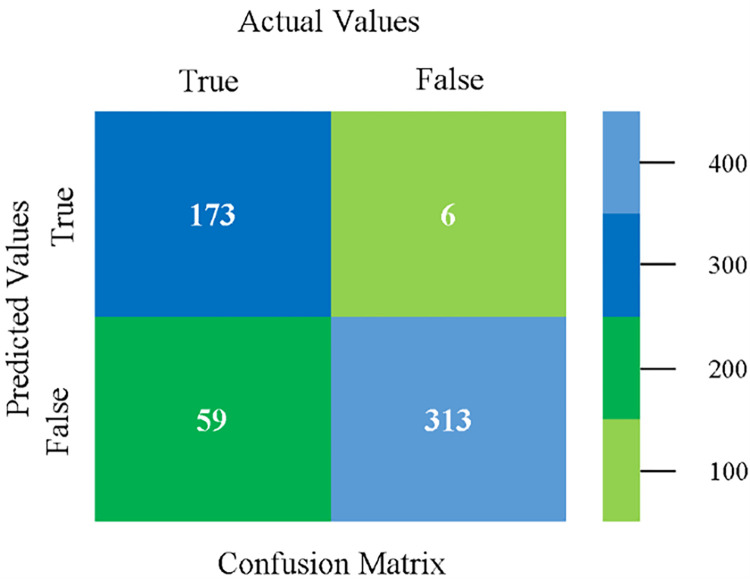
Confusion matrix of SMO classifier based on HDD multi in accuracy & fault prediction.

**Fig 65:**
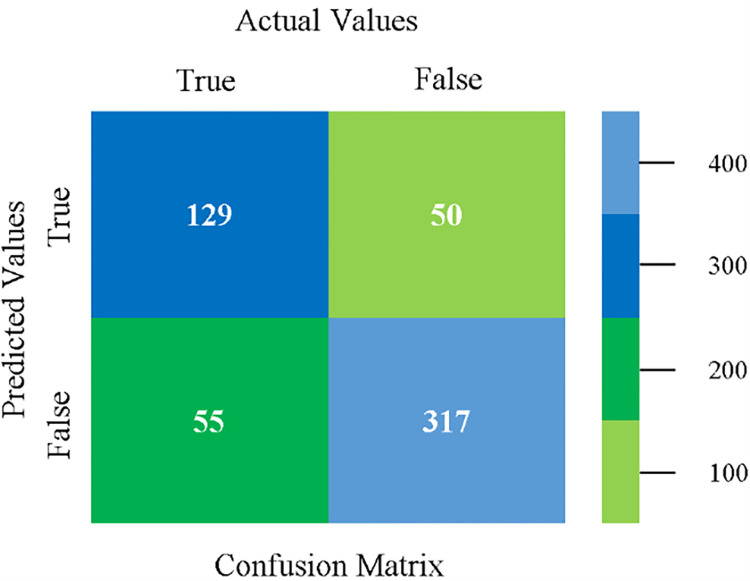
Confusion matrix of KNN classifier based on HDD multi in accuracy & fault prediction.

**Fig 66:**
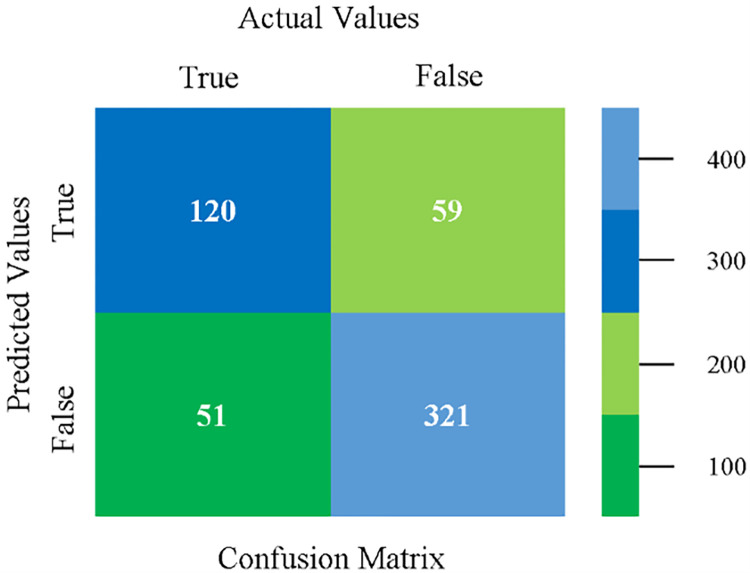
Confusion matrix of RF classifier based on HDD multi in accuracy & fault prediction.

**Fig 67:**
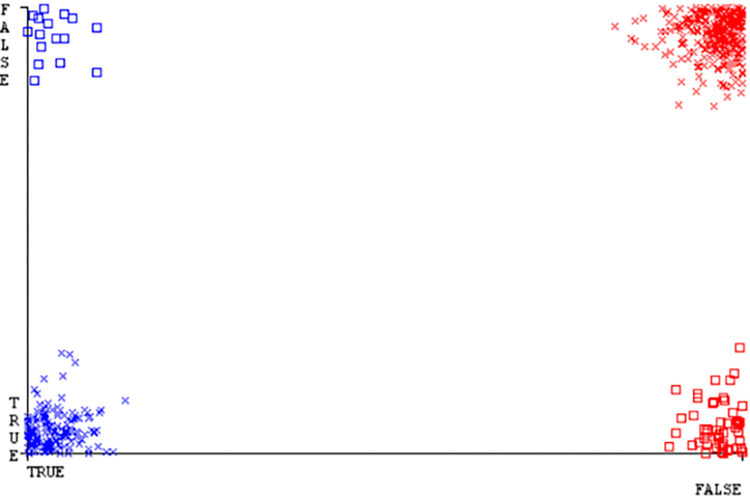
Classifier errors of NB classifier based on HDD multi in accuracy & fault prediction.

**Fig 68:**
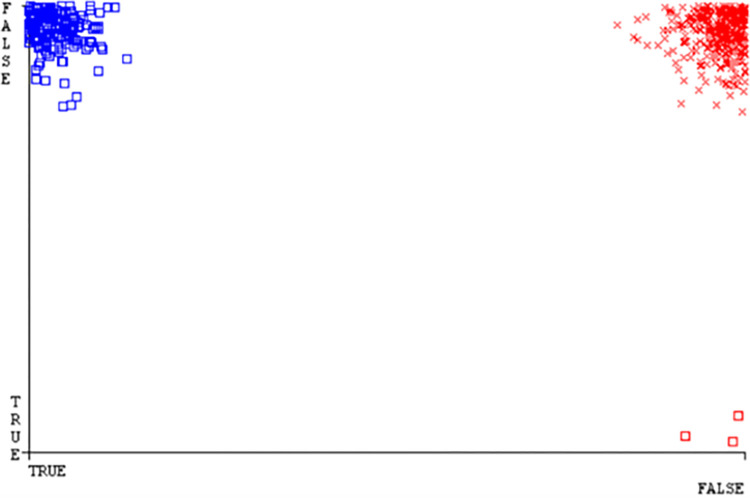
Classifier errors of LIBSVM classifier based on HDD multi in accuracy & fault prediction.

**Fig 69:**
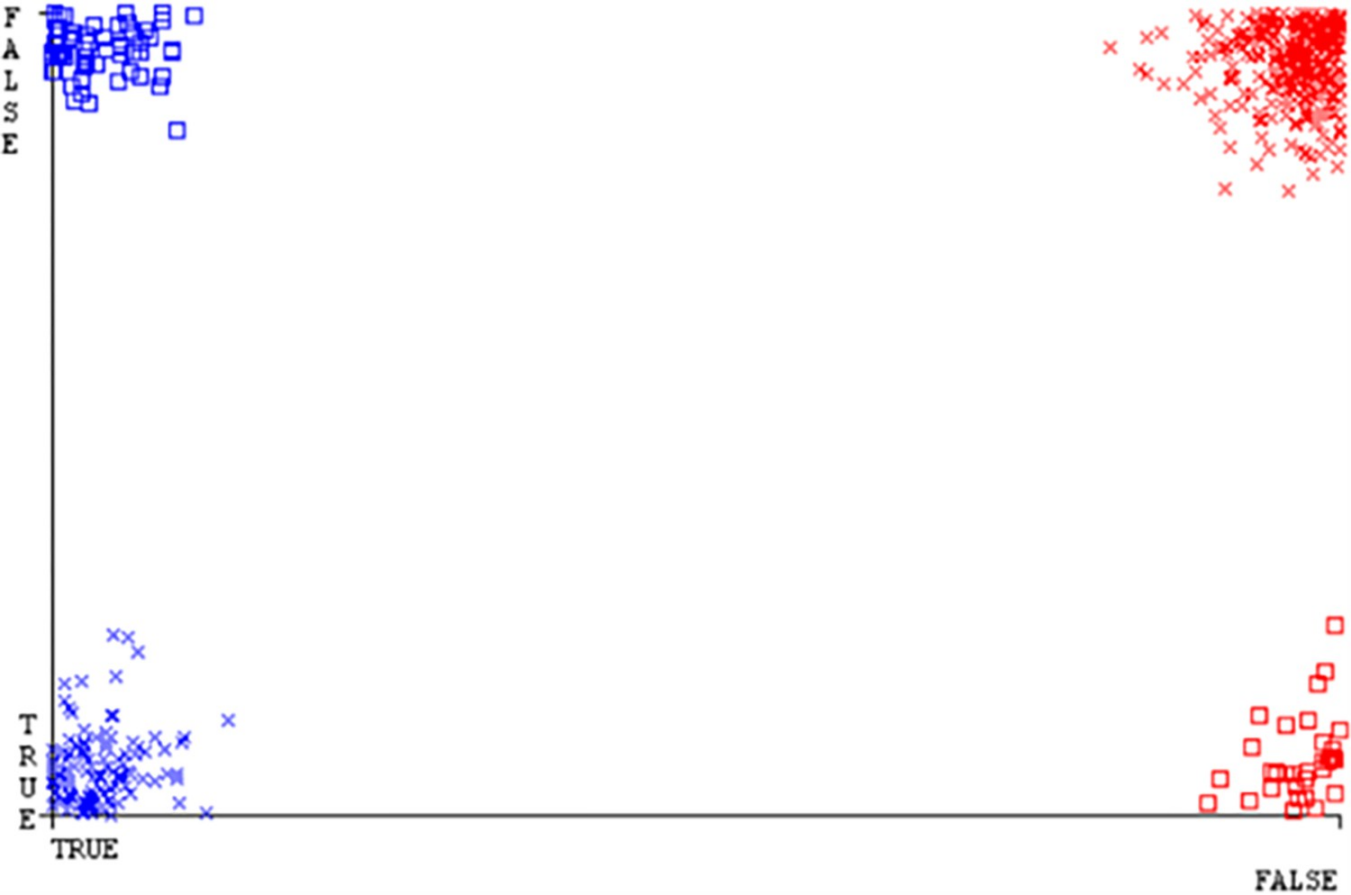
Classifier errors of MLR classifier based on HDD multi in accuracy & fault prediction.

**Fig 70:**
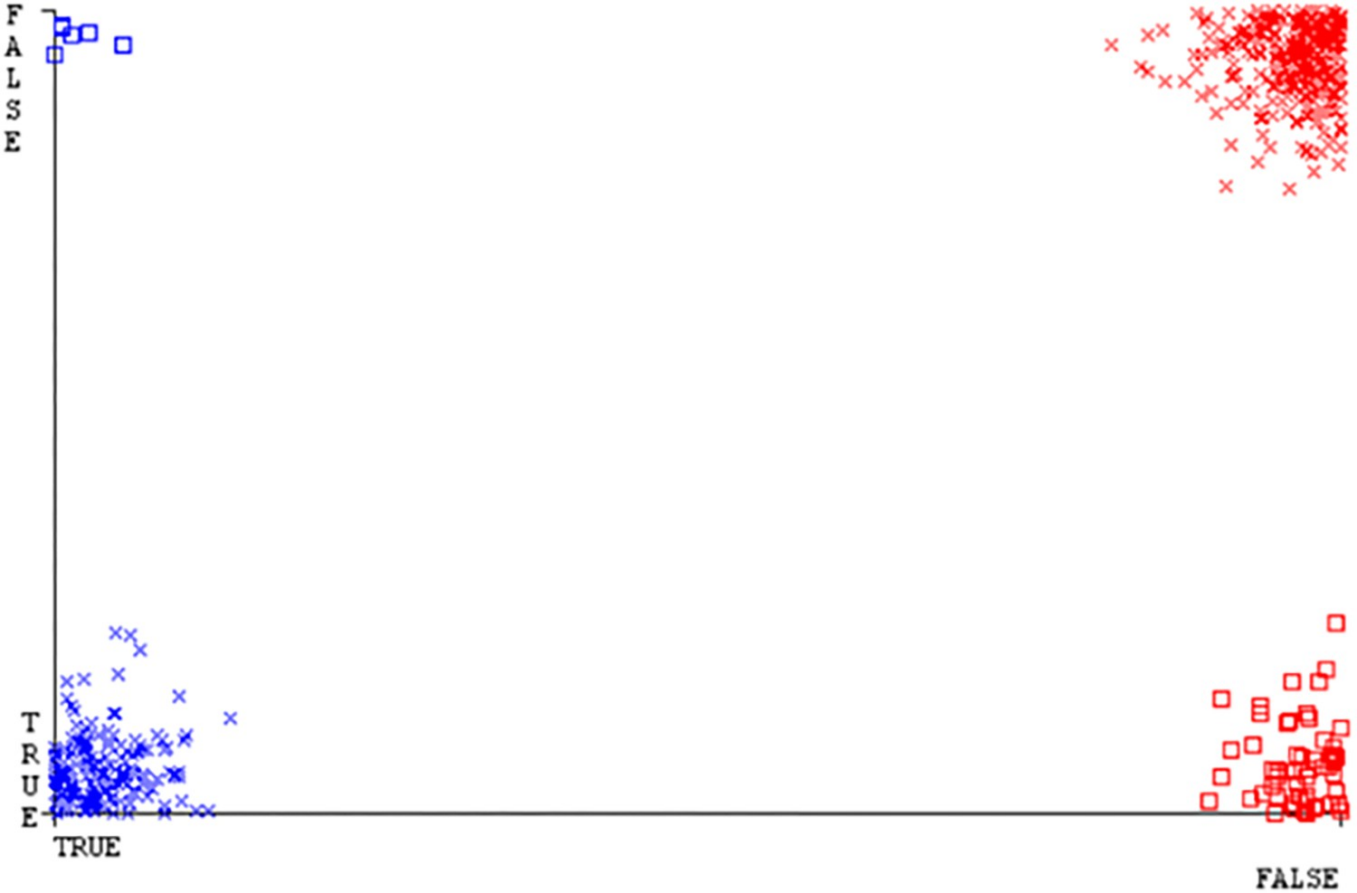
Classifier errors of SMO classifier based on HDD multi in accuracy & fault prediction.

**Fig 71:**
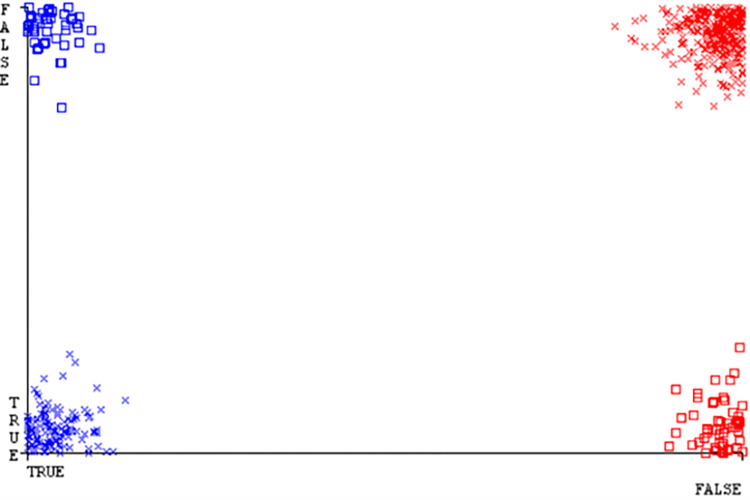
Classifier errors of KNN classifier based on HDD multi in accuracy & fault prediction.

**Fig 72:**
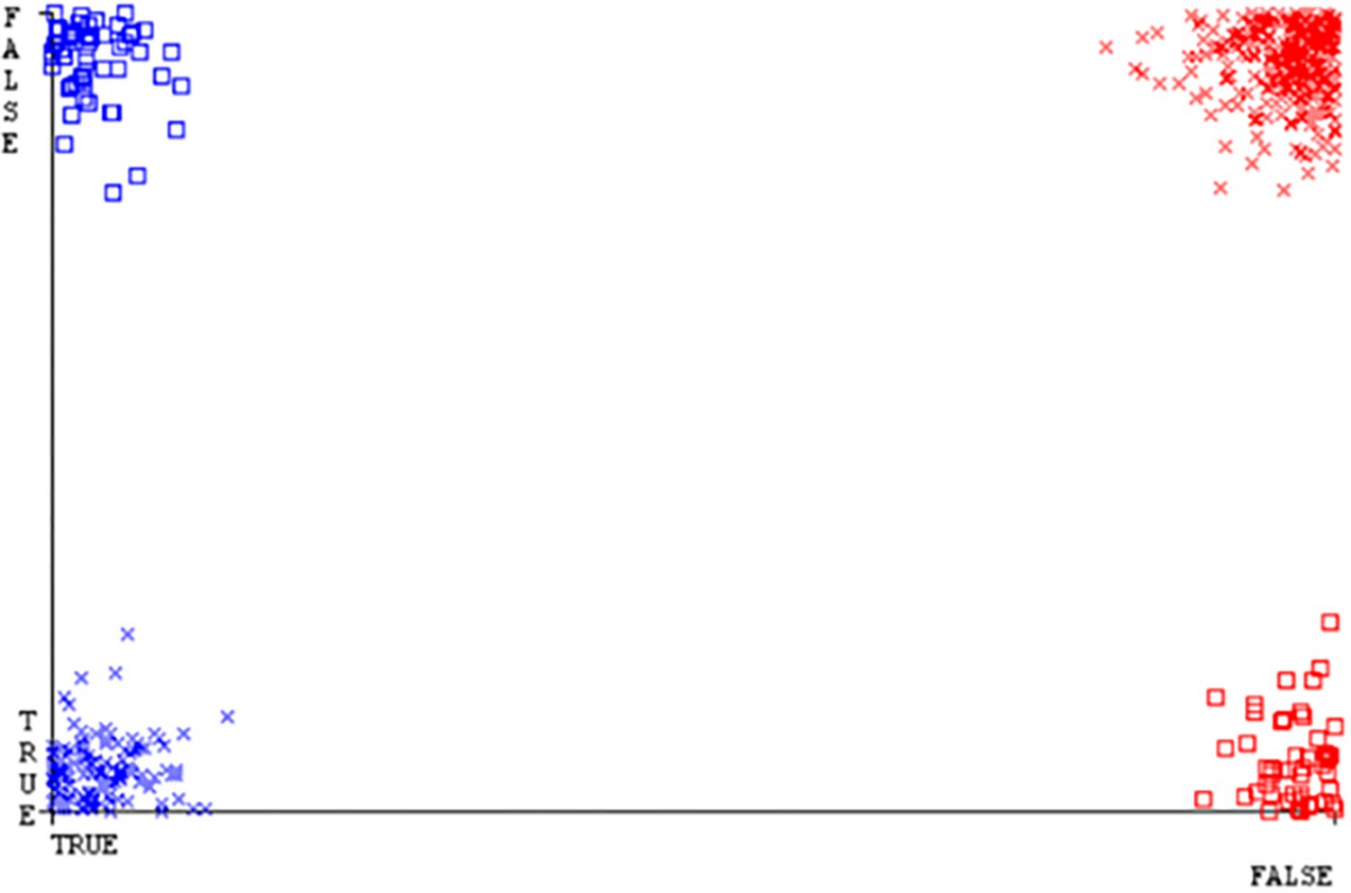
Classifier errors of RF classifier based on HDD multi in accuracy & fault prediction.

#### Secondary dataset CPU-mem mono block-I

(Figs 9–12) depict a comparison of the results of NB, LibSVM, MLR, SMO, KNN, and RF in CPU-Mem Mono-related detailed accuracy by class (True/False) and prediction on test split additional data validation.

The confusion matrix is used to calculate Accuracy, Precision, Recall, and F-Measure. It is used as an efficient technique for the classification of attributes based on qualitative response categories. (Figs 13–18) show the confusion matrix related to accuracy & fault prediction, achieved through NB, LibSVM, MLR, SMO, KNN, and RF. The following confusion matrix indicates that the NB classification model gives the highest percentage of accuracy & less fault prediction on CPU-Mem Mono.

(Figs 19–24) represent the error of the classifier which shows the values corresponding to true positive, true negative, false positive, and false negative values. In (Figs 19–24) the square box represents the errors in the actual class versus the predicted class.

#### Secondary dataset CPU-mem multi block-II

(Figs 25–28) depicts a comparison of the results of NB, LibSVM, MLR, SMO, KNN, and RF in CPU-Mem Multi related to detailed accuracy by class (True/False) and prediction on test split additional data validation.

The confusion matrix is used to calculate Accuracy, Precision, Recall, and F-Measure. It is used as an efficient technique for the classification of attributes based on qualitative response categories. (Figs 29–34) show the confusion matrix related to accuracy & fault prediction, achieved through NB, LibSVM, MLR, SMO, KNN, and RF. The following confusion matrix indicates that the NB classification model gives the highest percentage of accuracy & less fault prediction on CPU-Mem Multi.

(Figs 35–40) represent the error of the classifier which shows the values corresponding to true positive, true negative, false positive, and false negative values. In (Figs 35–40) the square box represents the errors in the actual class versus the predicted class.

#### Secondary dataset HDD mono block-III

(Figs 41–44) depicts a comparison of the results of NB, LibSVM, MLR, SMO, KNN, and RF in HDD Mono related to detailed accuracy by class (True/False) and prediction on test split additional data validation.

The confusion matrix is used to calculate Accuracy, Precision, Recall, and F-Measure. It is used as an efficient technique for the classification of attributes based on qualitative response categories. (Figs 45–50) show the confusion matrix related to accuracy & fault prediction, achieved through NB, LibSVM, MLR, SMO, KNN, and RF. The following confusion matrix indicates that the SMO classification model gives the highest percentage of accuracy & less fault prediction on HDD Mono.

(Figs 51–56) represent the error of the classifier which shows the values corresponding to true positive, true negative, false positive, and false negative values. In (Figs 51–56) the square box represents the errors in the actual class versus the predicted class.

#### Secondary dataset HDD multi block-IV

(Figs 57–60) show the result comparison of NB, LibSVM, MLR, SMO, KNN, and RF in HDD Multi-related detailed accuracy by class (True/False) and prediction on test split further data validation.

The confusion matrix is used to calculate Accuracy, Precision, Recall, and F-Measure. It is used as an efficient technique for the classification of attributes based on qualitative response categories. (Figs 61–66) show the confusion matrix related to accuracy & fault prediction, achieved through NB, LibSVM, MLR, SMO, KNN, and RF. The following confusion matrix indicates that the SMO classification model gives the highest percentage of accuracy & less fault prediction on HDD Multi.

(Figs 67–72) represent the error of the classifier which shows the values corresponding to true positive, true negative, false positive, and false negative values. In (Figs 67–72) the square box represents the errors in the actual class versus the predicted class.

### Comparison of classification models on a primary dataset

We are presenting results associated with different classifiers using the STATUS class in the primary dataset. For classification models, we opted for NB, LibSVM, MLR, RF, KNN, & SMO with the poly kernel.

**Fig 73:**
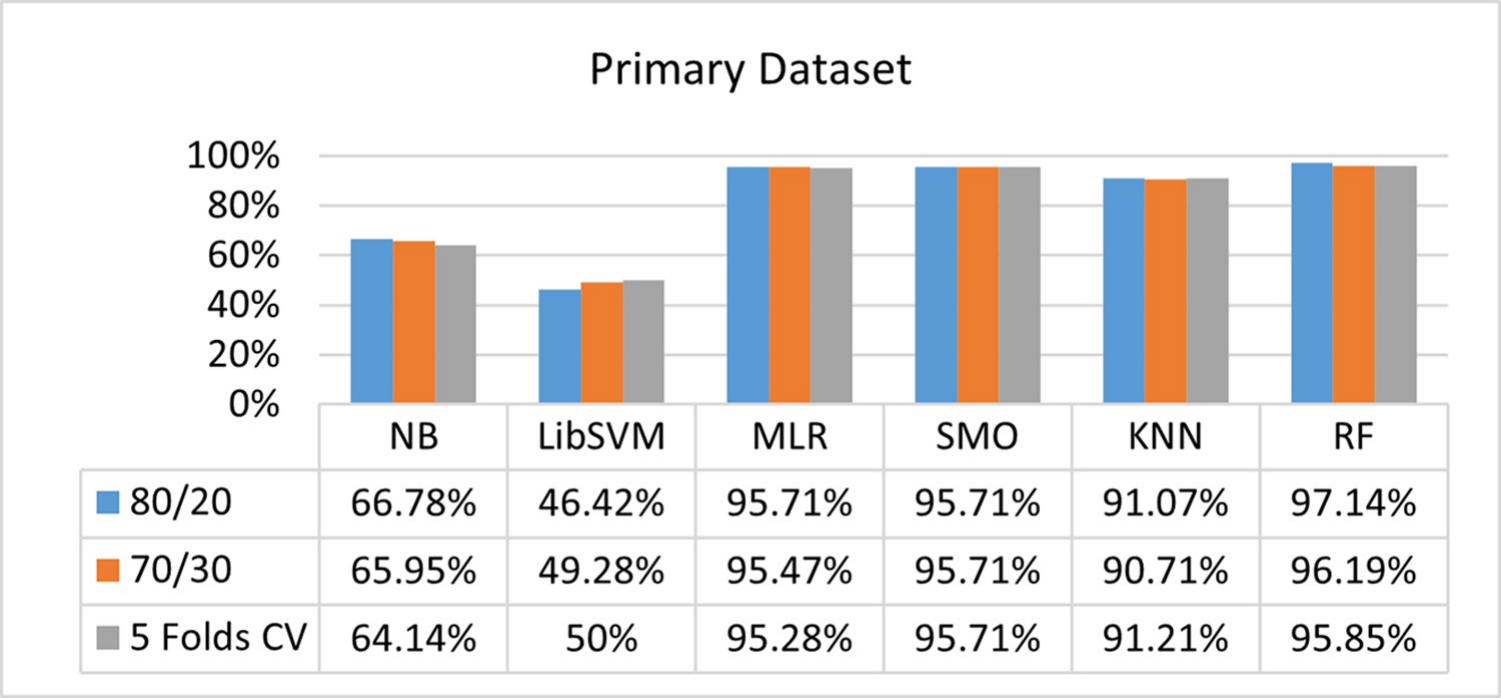
Accuracy by class (repair/failure) of the primary dataset on ML classifiers.

**Fig 74:**
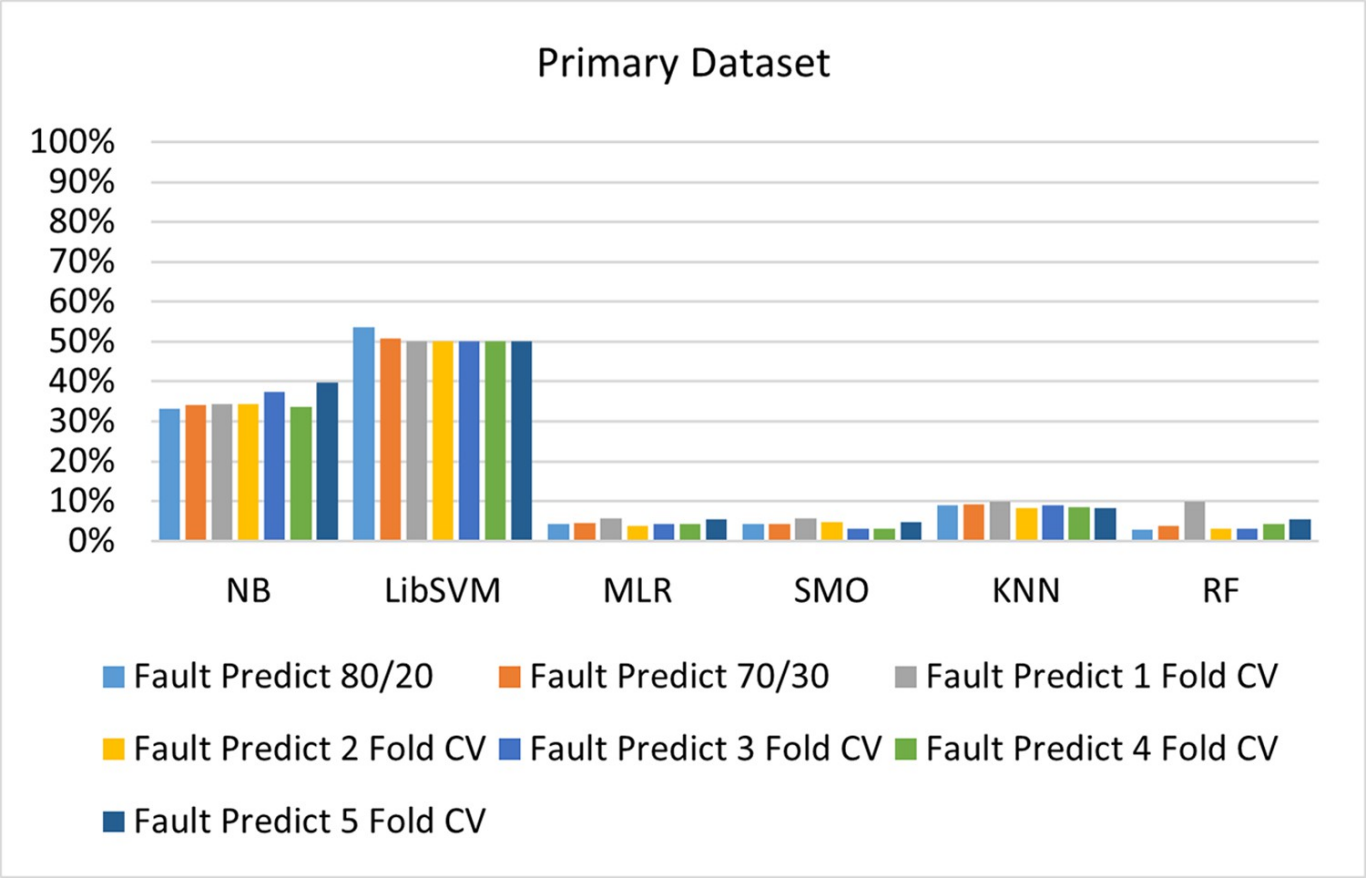
Fault prediction by class of (repair/failure) of the primary dataset on ML classifiers.

**Fig 75:**
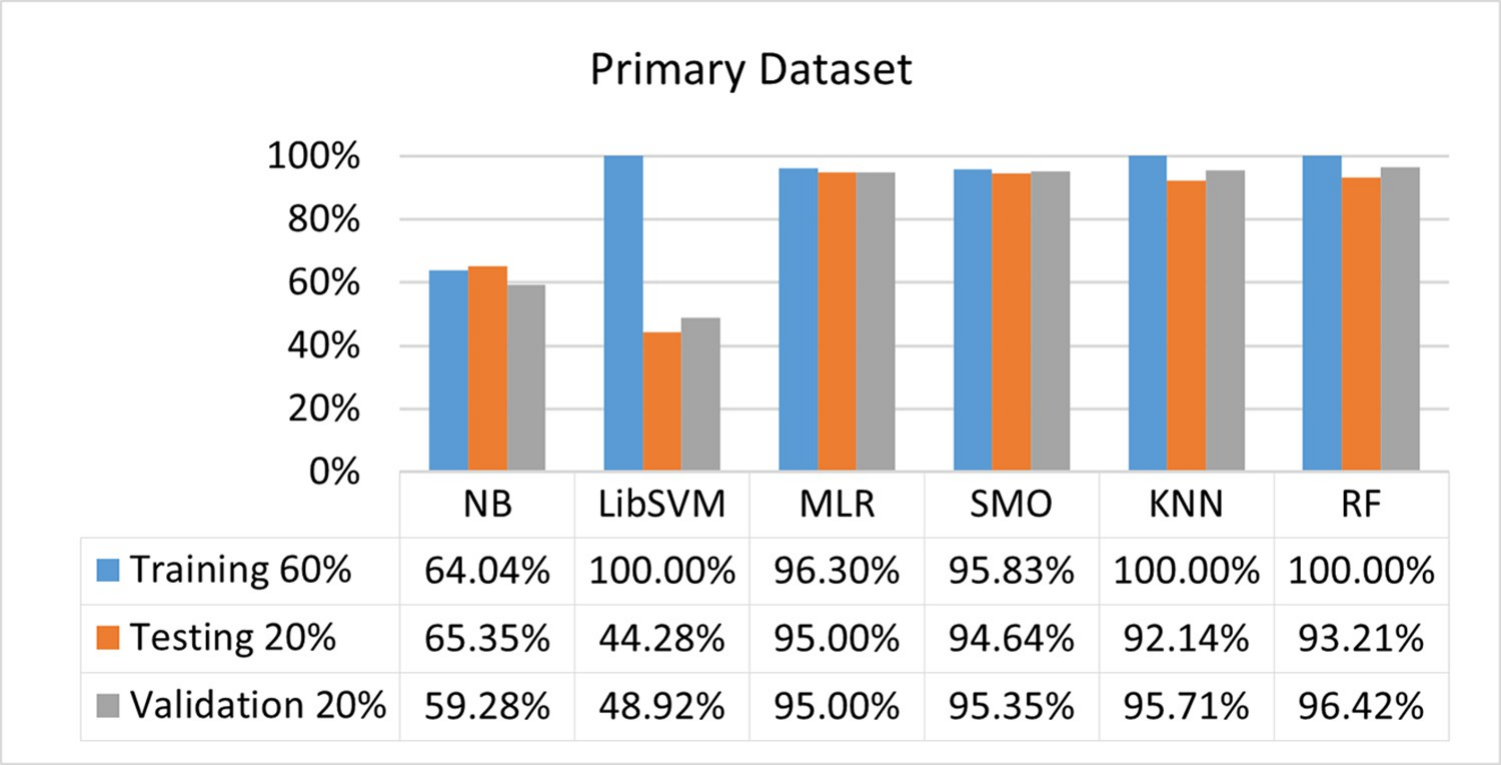
Accuracy by class (repair/failure) of the primary dataset on ML classifiers related to DV results.

**Fig 76:**
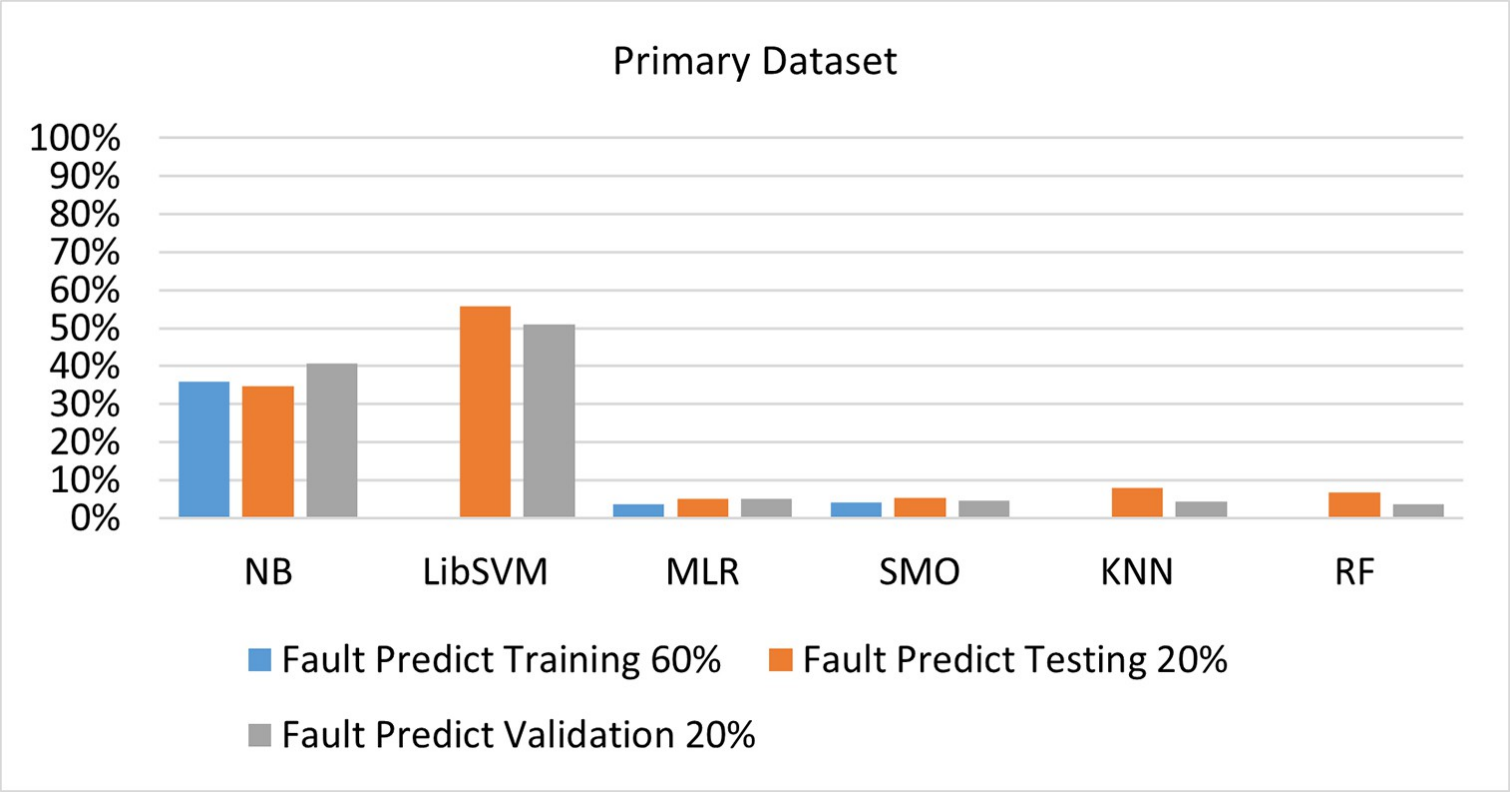
Fault prediction by class of (repair/failure) of the primary dataset on ML classifiers related to DV results.

The RF classifier gives the highest percentage of accuracy and less fault prediction in terms of 80/20 (97.14%), 70/30 (96.19%), and 5 folds cross-validation (95.85%) in the primary data results, but the algorithm complexity (0.17 seconds) is not good. In terms of 80/20 (95.71%), 70/30 (95.71%), and 5 folds cross-validation (95.71%), SMO has the second highest accuracy and less fault prediction, but the algorithm complexity is good (0.3 seconds). The difference in accuracy and less fault prediction between RF and SMO is only (.13%), and the difference in time complexity is (14 seconds).

(Figs 73–76) show a comparison of the results of NB, LibSVM, MLR, SMO, KNN, and RF in the Primary Dataset for detailed accuracy by class (Repair/Failure) and prediction on test split additional data validation.

**Fig 77:**
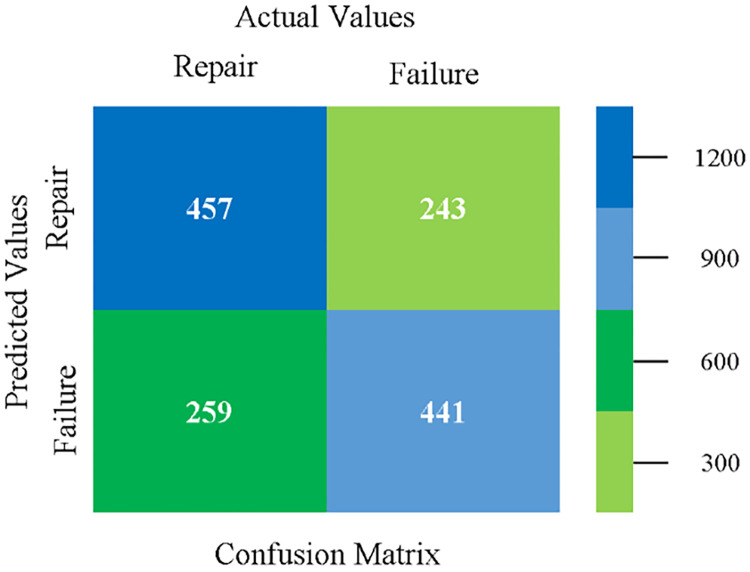
Confusion matrix of NB classifier based on primary data in accuracy & fault prediction.

**Fig 78:**
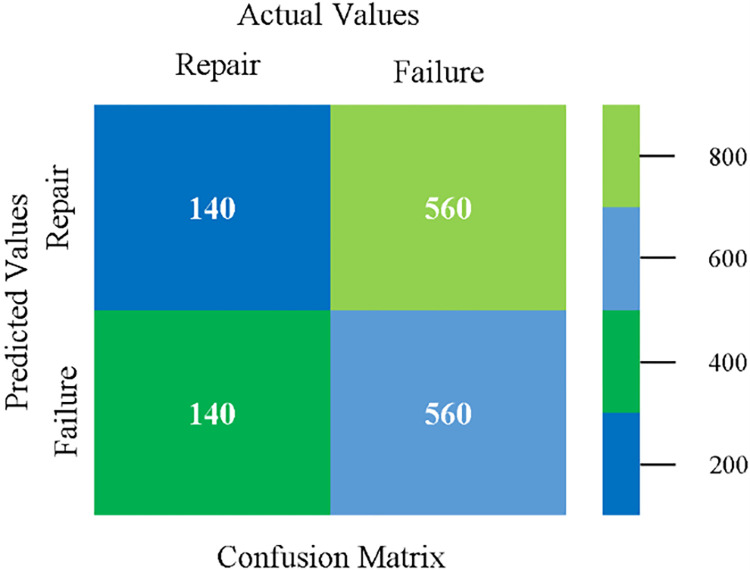
Confusion matrix of LIBSVM classifier basd on primary data in accuracy & fault prediction.

**Fig 79:**
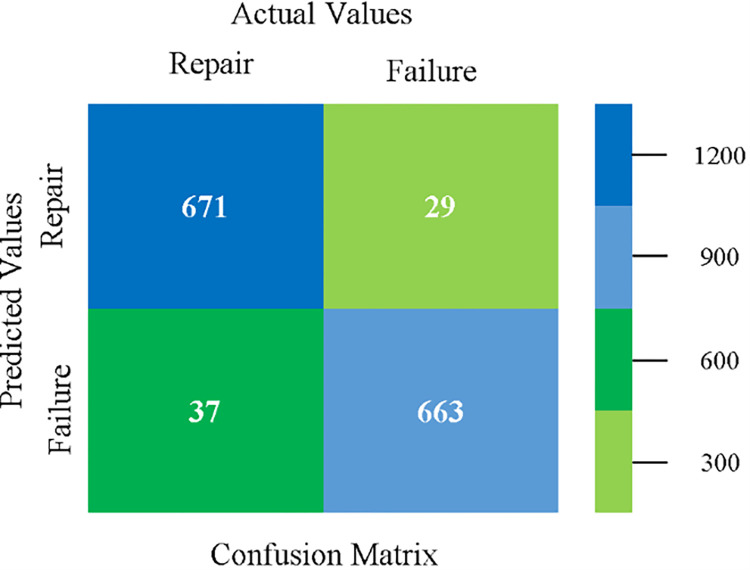
Confusion matrix of MLR classifier based on primary data in accuracy & fault prediction.

**Fig 80:**
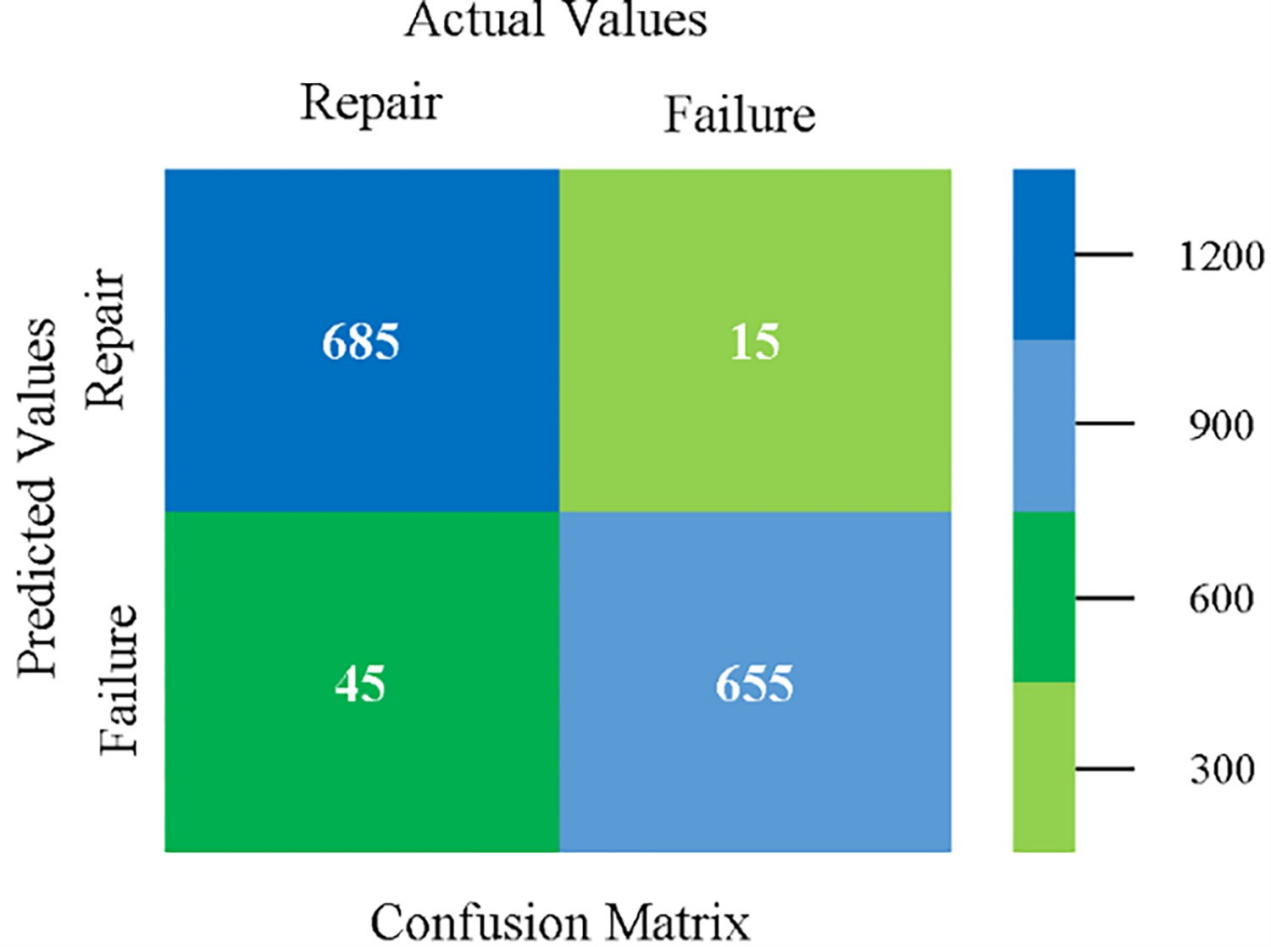
Confusion matrix of SMO classifier based on primary data in accuracy & fault prediction.

**Fig 81:**
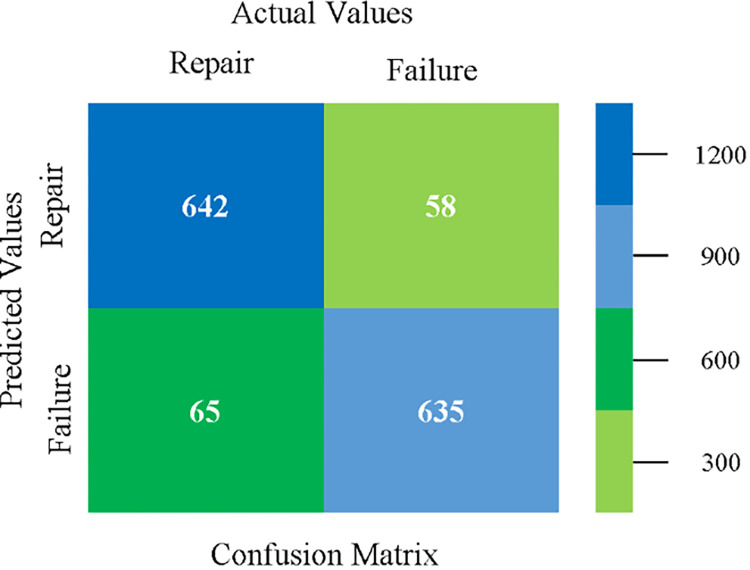
Confusion matrix of KNN classifier based on primary data in accuracy & fault prediction.

**Fig 82:**
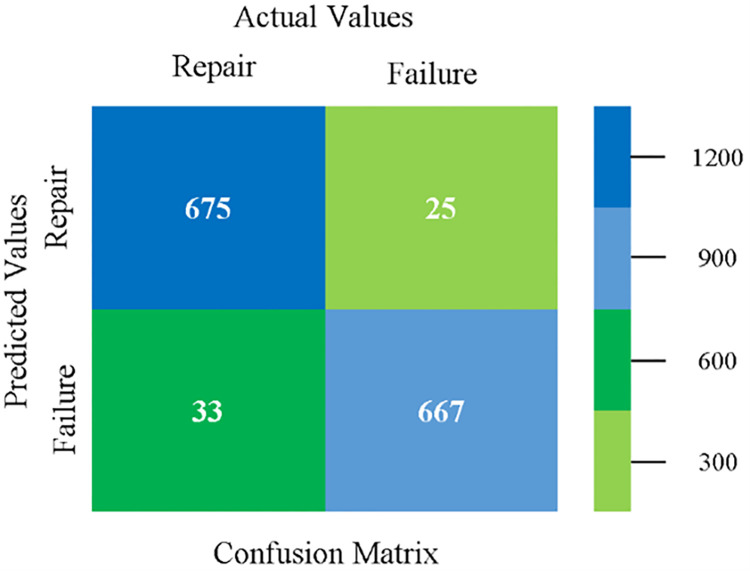
Confusion matrix of RF classifier based on primary data in accuracy & fault prediction.

The confusion matrix is used to calculate Accuracy, Precision, Recall, and F-Measure. It is used as an efficient technique for the classification of attributes based on qualitative response categories. (Figs 77–82) show the confusion matrix related to accuracy & fault prediction, achieved through NB, LibSVM, MLR, SMO, KNN, and RF. The following confusion matrix indicates that the RF classification model gives the highest percentage of accuracy & less fault prediction on the primary dataset, but the algorithm complexity (0.17 seconds) is not good.

**Fig 83:**
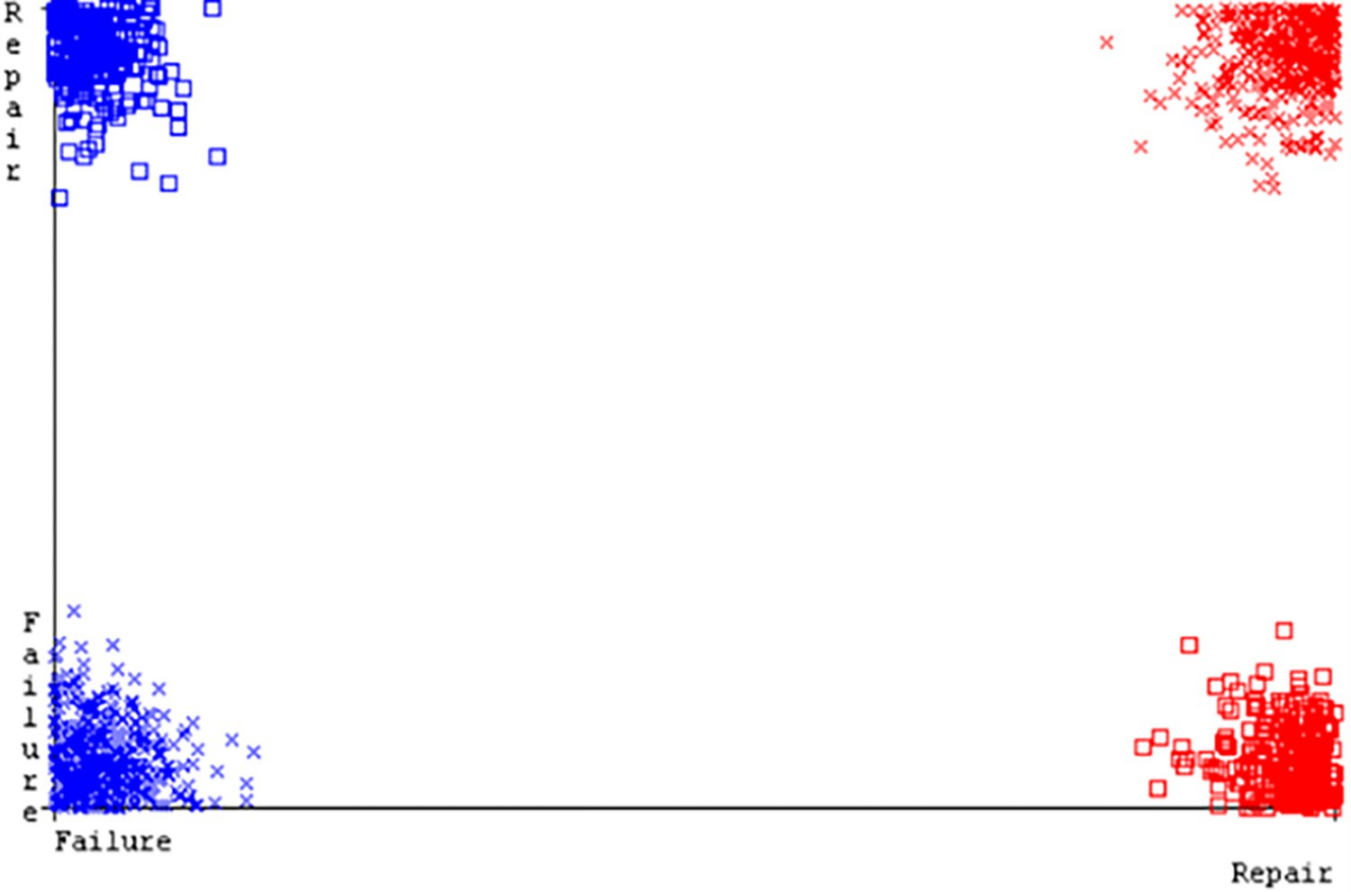
Classifier errors of NB classifier based on primary data in accuracy & fault prediction.

**Fig 84:**
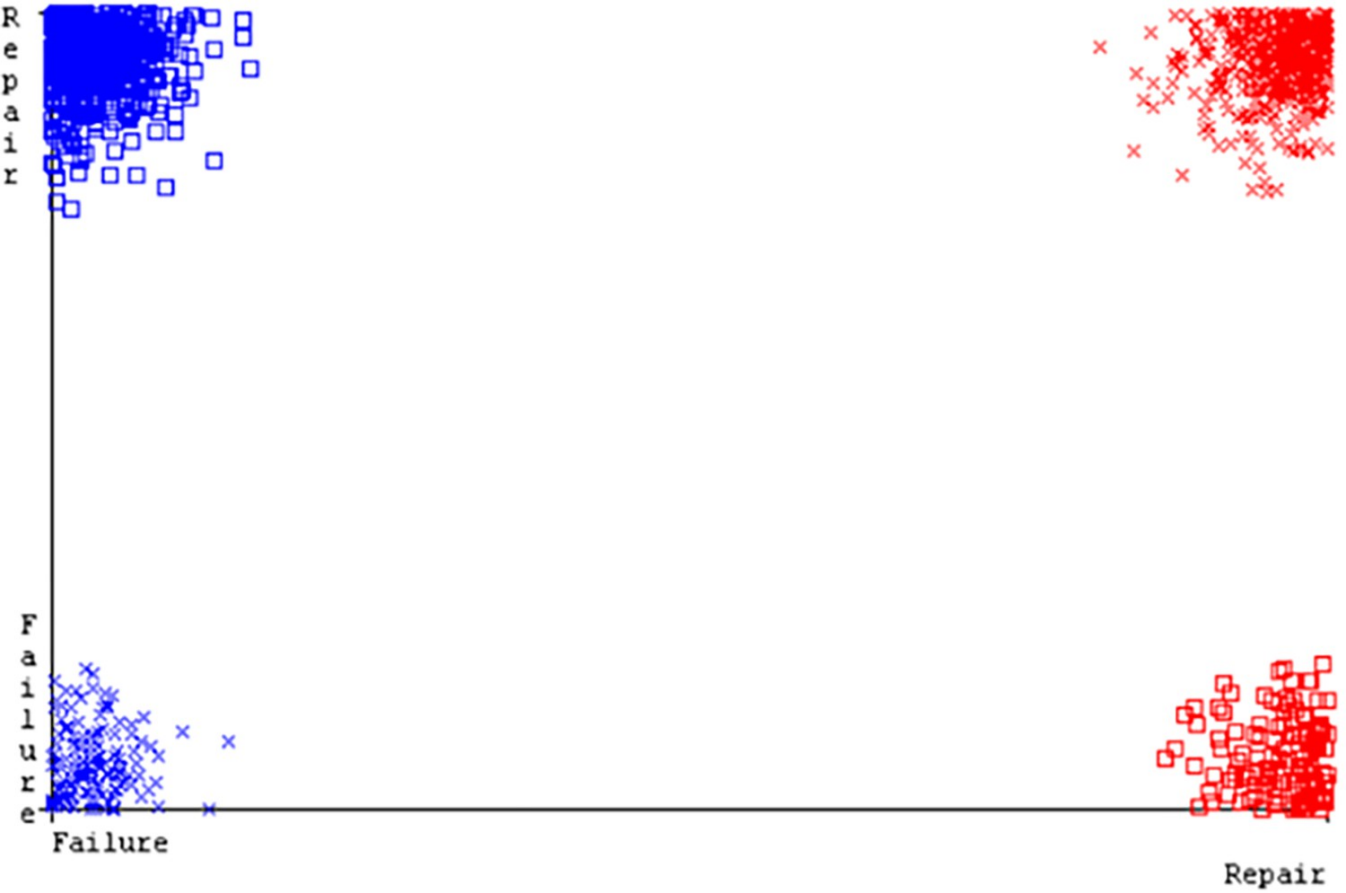
Classifier errors of LIBSVM classifier based on primary data in accuracy & fault prediction.

**Fig 85:**
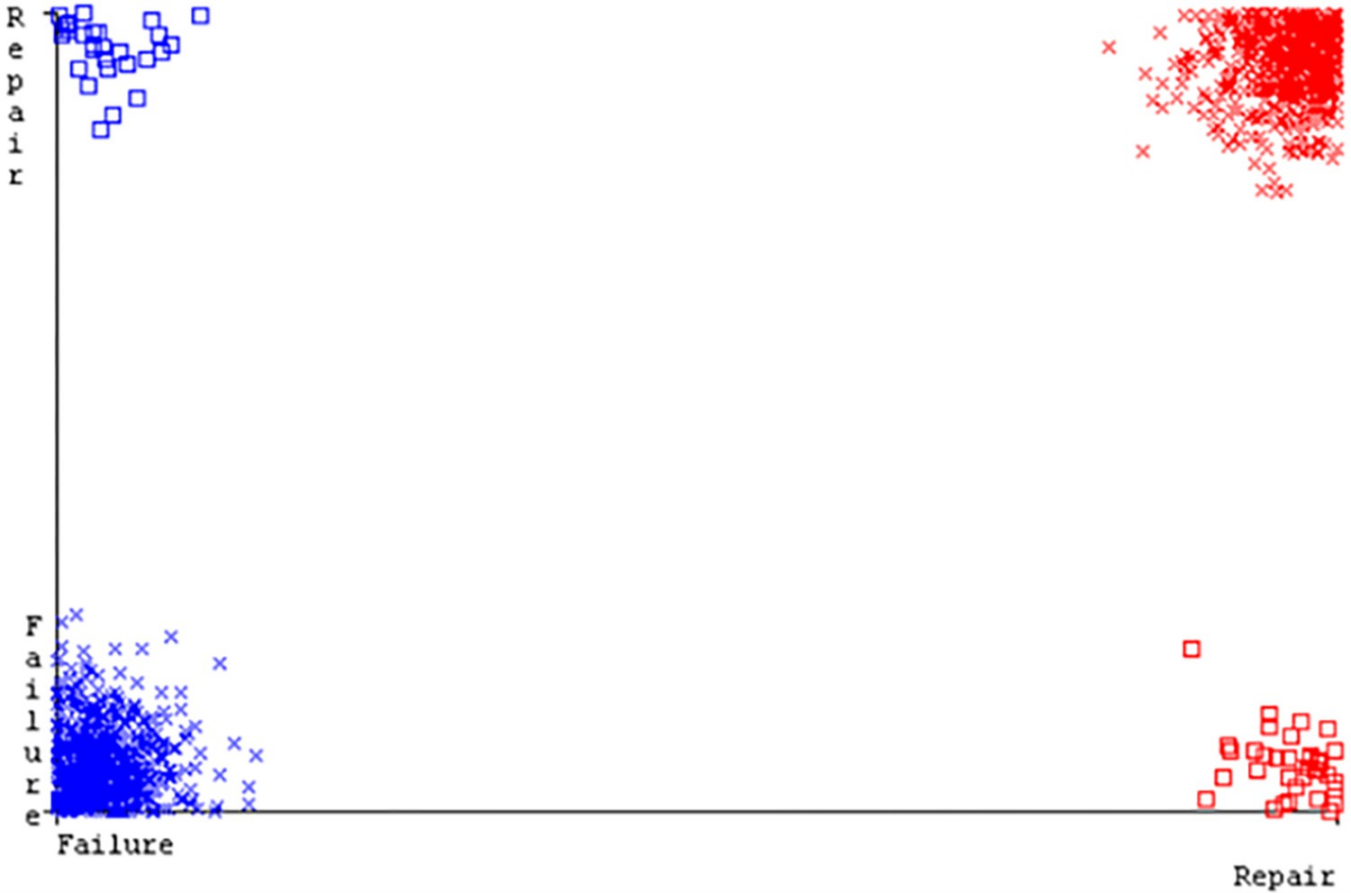
Classifier errors of MLR classifier based on primary data in accuracy & fault prediction.

**Fig 86:**
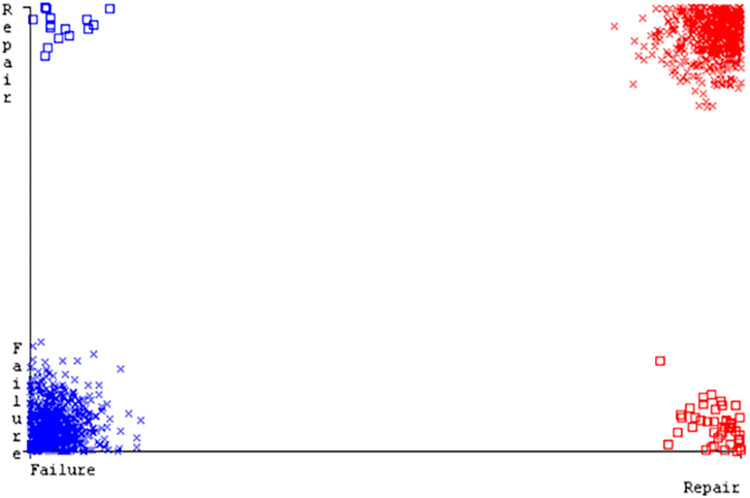
Classifier errors of SMO classifier based on primary data in accuracy & fault prediction.

**Fig 87:**
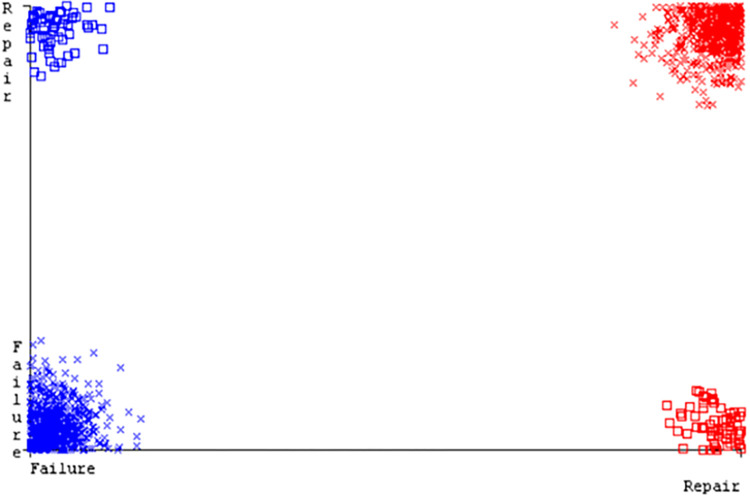
Classifier errors of KNN classifier based on primary data in accuracy & fault prediction.

**Fig 88:**
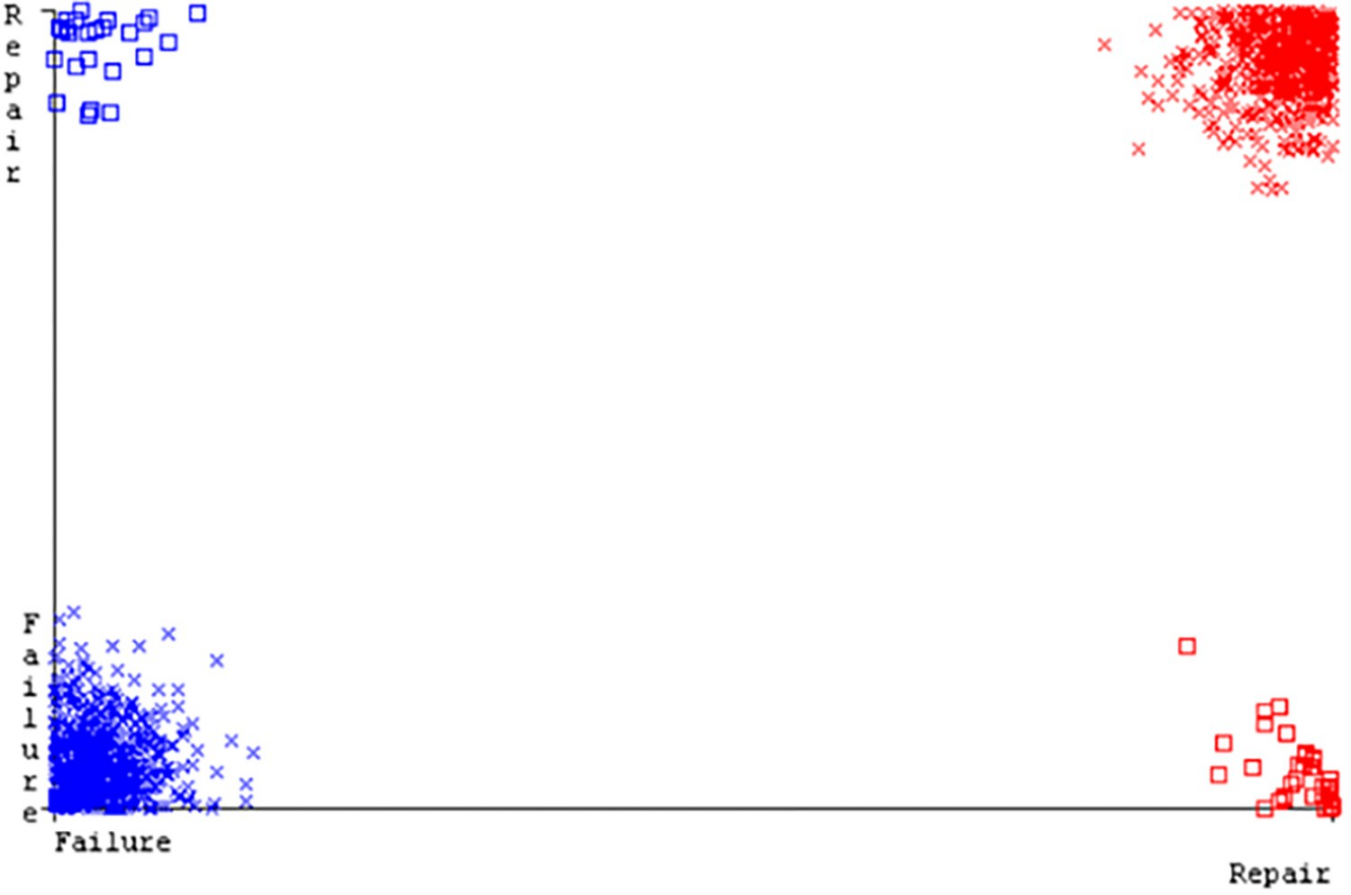
Classifier errors of RF classifier based on primary data in accuracy & fault prediction.

SMO gives the second-highest accuracy and less fault prediction but the algorithm complexity is good (0.3 seconds). The accuracy and less fault prediction difference between RF and SMO are just (.13%) and the time complexity difference is (14 seconds). (Figs 83–88) represent the error of the classifier which shows the values corresponding to true positive, true negative, false positive, and false negative values. In (Figs 83–88) the square box represents the errors in the actual class versus the predicted class.

### Modified sequential minimal optimization results

**Fig 89:**
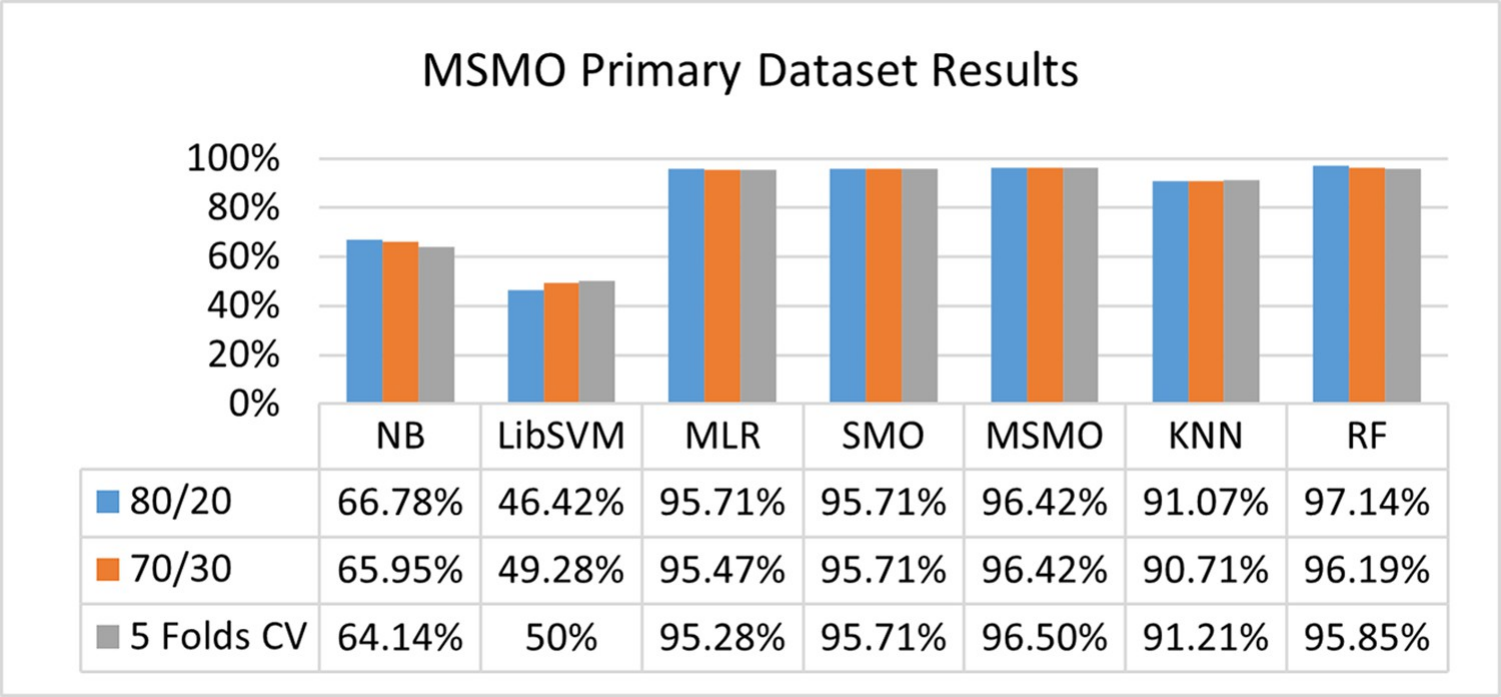
Comparison of ML classifiers with MSMO accuracy by class (repair/failure) of the primary dataset.

**Fig 90:**
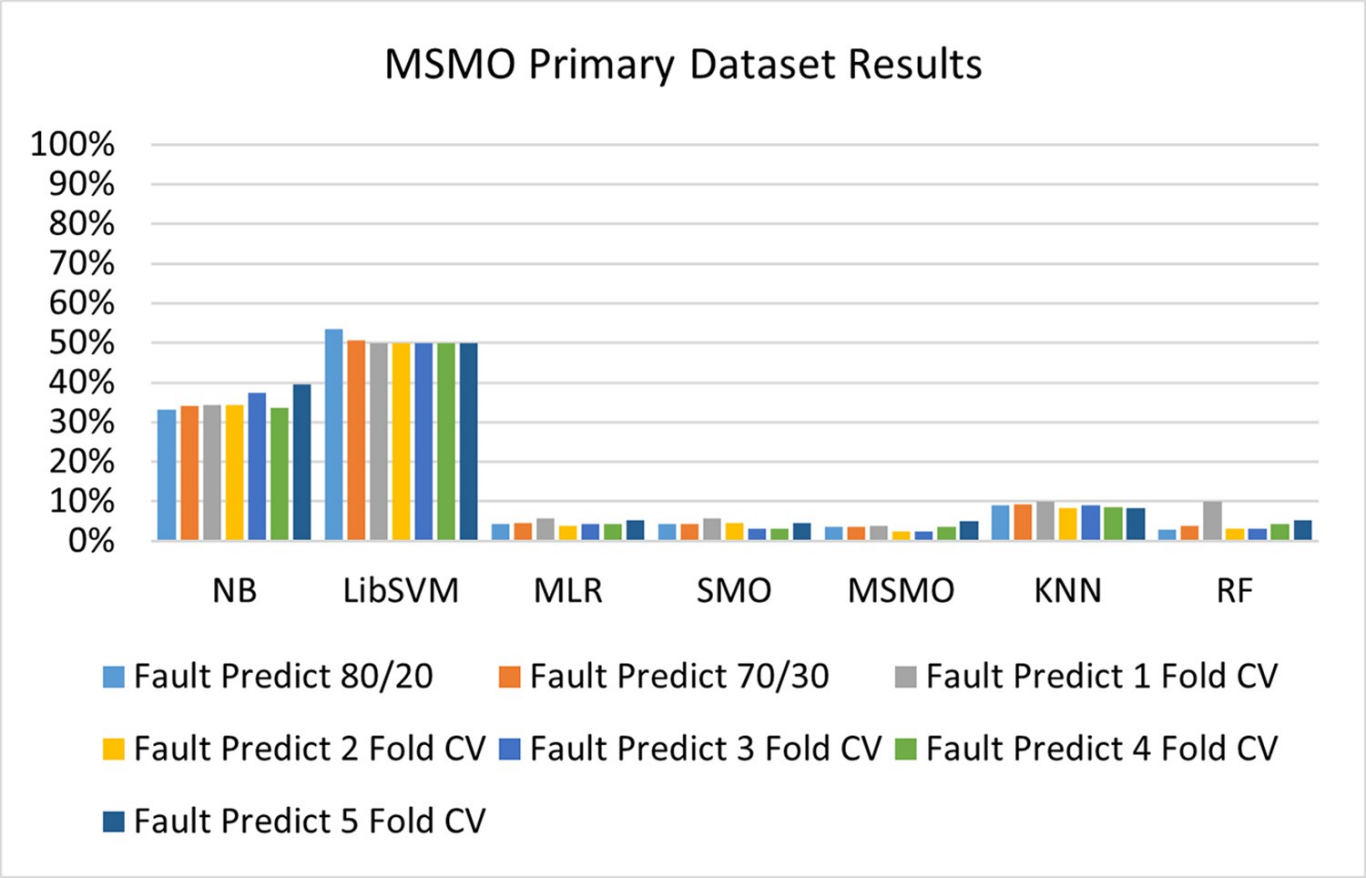
Comparison of ML classifiers with MSMO fault prediction by class (repair/failure) of the primary dataset.

**Fig 91:**
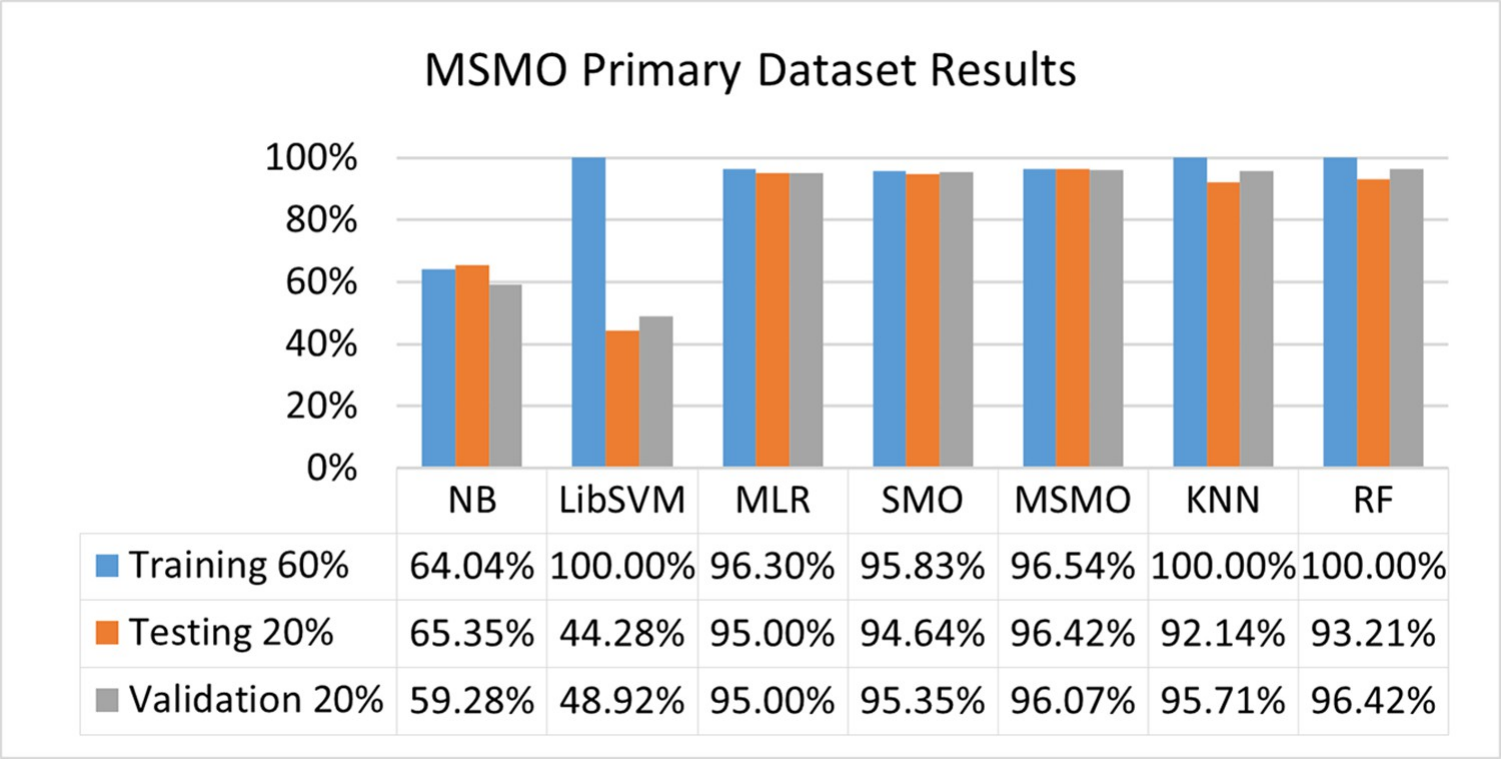
Comparison of ML classifiers with MSMO accuracy by class of primary dataset related to DV results.

**Fig 92:**
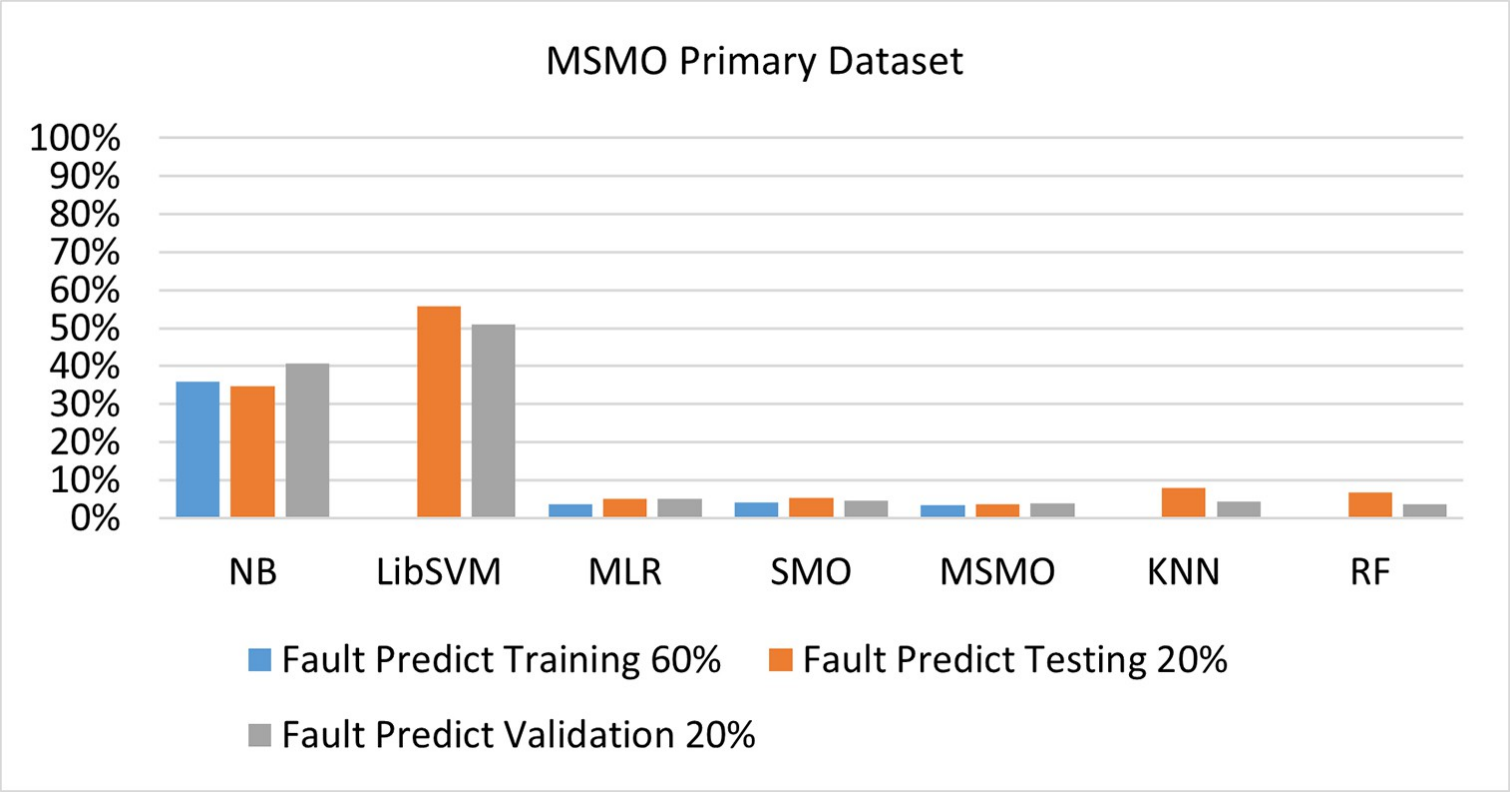
Comparison of ML classifiers with MSMO fault prediction by class of primary dataset related to DV results.

In this subsection, the results of the classification of the primary dataset results are shown in (Figs 89–92) indicating that the MSMO classification model gives the highest accuracy & less fault prediction errors in terms of 80/20 (96.42%), 70/30 (96.42%), & 5 fold cross validation (96.50%). The MSMO time complexity of the algorithm is (0.44 seconds) after modification.

(Figs 89–92) depicts a comparison of the results of NB, LibSVM, MLR, SMO, MSMO, KNN, and RF in the Primary Dataset in terms of detailed accuracy by class (Repair/Failure) and prediction on test split additional data validation.

**Fig 93:**
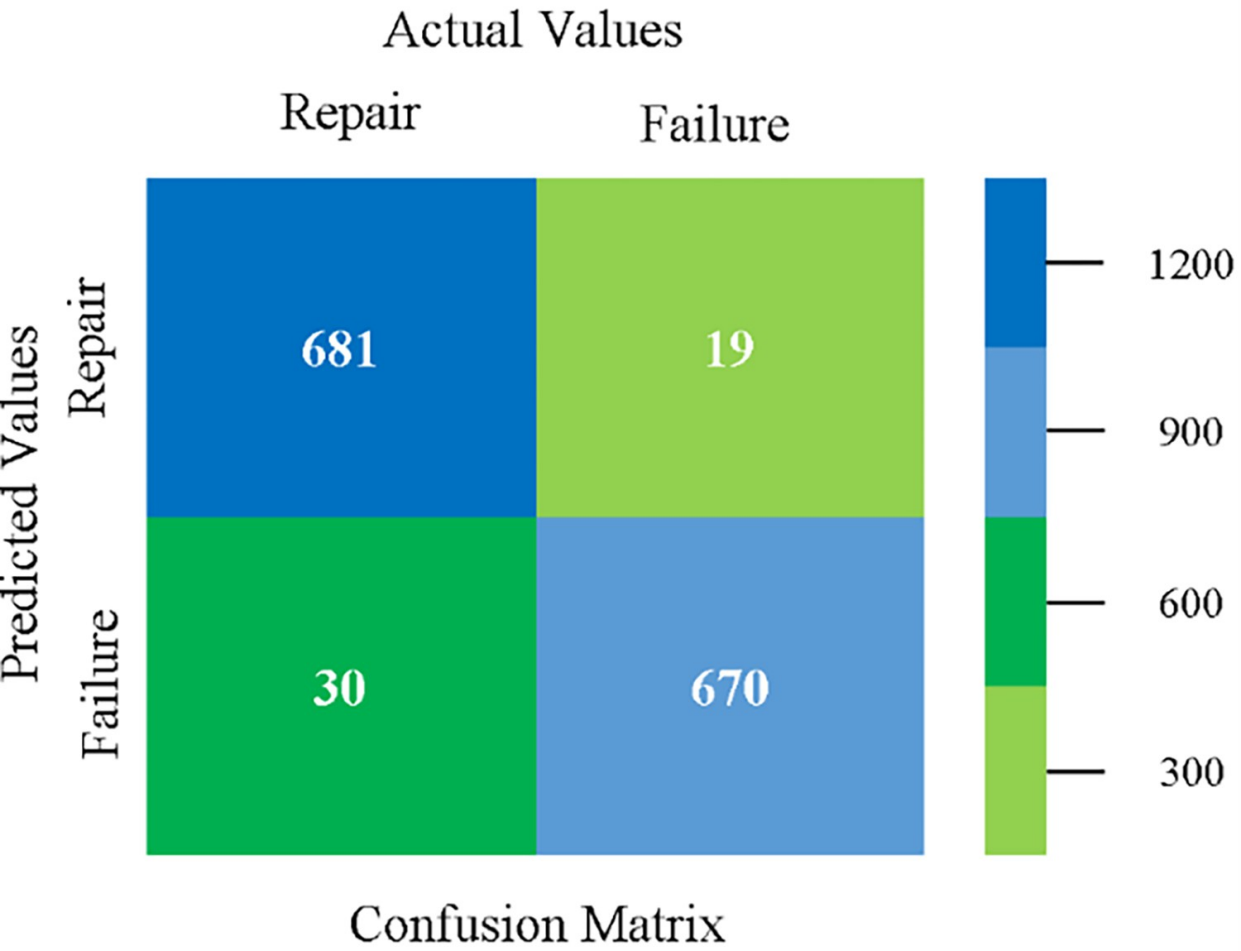
Confusion matrix of MSMO classifier based on primary data in high accuracy & less fault prediction error.

**Fig 94:**
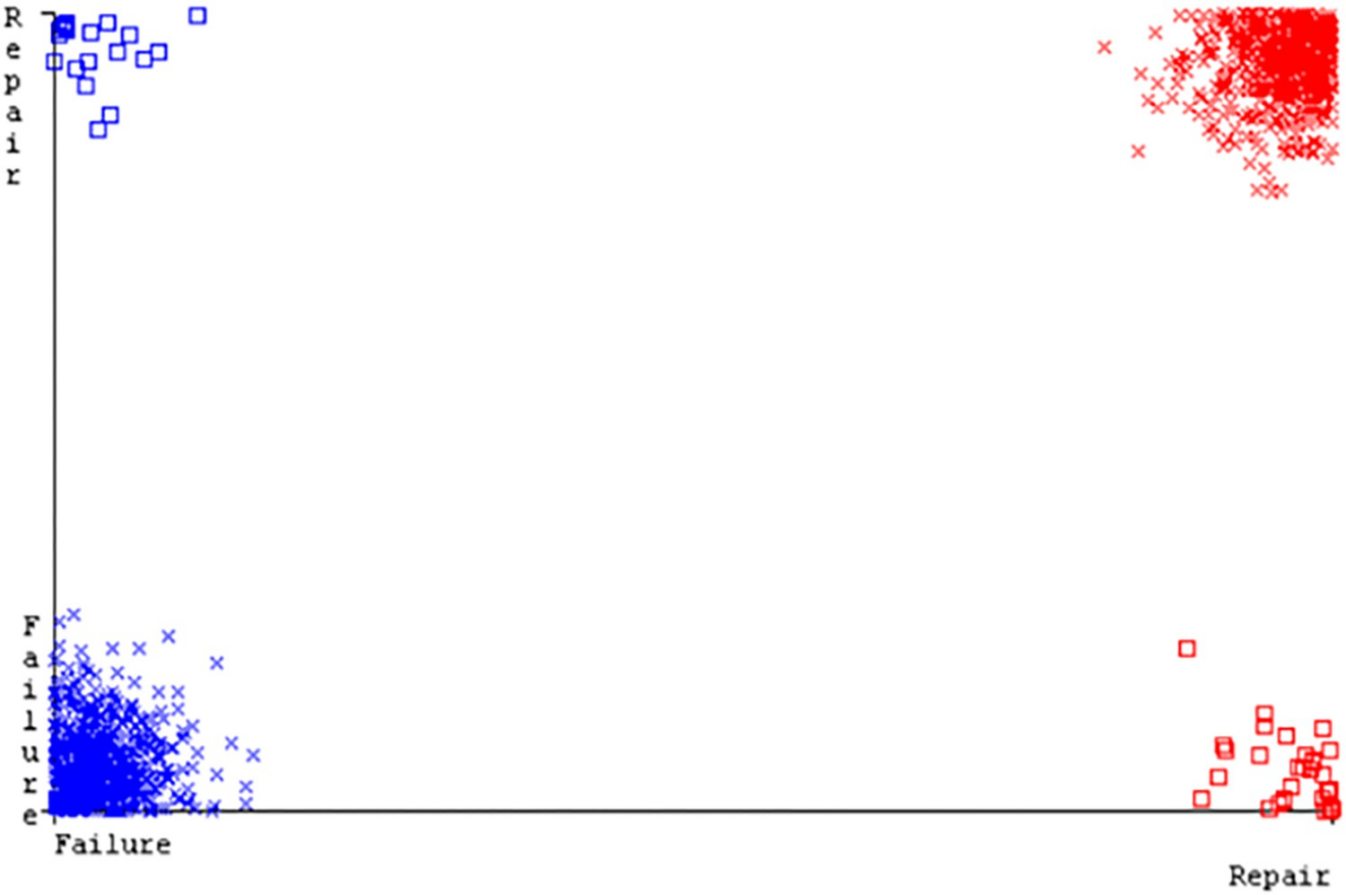
Classifier errors of MSMO classifier based on primary data in accuracy & fault prediction.

The confusion matrix is used to calculate Accuracy, Precision, Recall, and F-Measure. It is used as an efficient technique for the classification of attributes based on qualitative response categories. (Fig 93) shows the confusion matrix related to accuracy & fault prediction, achieved through MSMO. The following confusion matrix indicates that the MSMO classification model gives the highest percentage of accuracy & less fault prediction error on the primary dataset against NB, LibSVM, MLR, SMO, KNN, and RF.

(Fig 94) represents the error of the classifier which shows the values corresponding to true positive, true negative, false positive, and false negative values. In (Fig 94) the square box represents the errors in the actual class versus the predicted class.

## Discussion

This study was carried out to achieve high accuracy and less fault perdition to achieve reliability. The MSMO classifier was created to ensure the smooth execution of the research.

An MSMO classifier was applied to the primary data. According to the MSMO classifier results, the proposed strategy outperforms the existing classifier in terms of accuracy and fault prediction. The obtained results in the primary data were compared to the existing NB, LibSVM, MLR, SMO, KNN, and RF classifiers. High accuracy with low fault prediction is the most important parameter for judging the classifier’s performance level.

Simulated results were compared to NB, LibSVM, MLR, SMO, KNN, and RF classifiers, and it was demonstrated that the proposed classifier performed more accurately and quickly, with 96.5% of instances correctly classified than the available classifiers. The proposed research’s innovation is a collection of techniques that have been linked to high accuracy and less fault prediction to achieve reliability. MSMO was proposed using parameter tuning, which is considered a novel approach.

### Contributions

Our work makes numerous contributions. We began by acquiring the HPC fault dataset and evaluating a fault classification method based on supervised machine learning. This dataset and all test environment details are publicly available for use by the community. The Antarex secondary dataset will be based on trace data from the homonymous experimental HPC system at ETH Zurich during fault injection, which will be used to undertake machine learning-based fault prediction studies for researchers. The dataset will be separated into two sections, one for CPU and memory-related benchmark apps and fault programs, and another for hard drive-related applications and fault programs. Antarex dataset will have four folders, one for each dataset block, namely CPU/Memory and HDD, in single-core and multi-core forms [3].

Second, we generated a primary dataset through the Weibull distribution approach. The Weibull distribution is also often employed as a time-to-failure model for reliability. It extends the exponential model by including non-constant failure rate functions. This contains both rising and falling failure rate curves and has been successfully utilized to explain both initial burnings and wear-out failures [4]. We have coded different parameters in the java platform for primary data generated using the Weibull distribution approach.

Third, our analysis is based on (Antarex Secondary Datasets) obtained from the ZONODO website and (Primary Datasets) generated using the Weibull distribution approach [3, 4]. We present the results of our experiments, which were designed to determine which ML algorithms provide better results in terms of high accuracy and less fault prediction.

As a fourth & final contribution, high accuracy, and less fault prediction error, have been achieved using the MSMO classifier which has a good impact on users related to the CC environment.

## Conclusions

The results of this study were associated with various classifiers using ISFAULT in secondary data and STATUS in primary data to achieve high accuracy and less fault prediction errors.

Secondary data results (CPU-Mem Mono) give the highest percentage of accuracy and less fault prediction on the NB classifier in terms of 80/20 (77.01%), 70/30 (76.05%), and 5 folds cross-validation (74.88%), and (CPU-Mem Multi) give the highest percentage of accuracy and less fault prediction on the NB classifier in terms of 80/20 (89.72%), 70/30 (90.28%), and 5 folds cross-validation (92.83%). Furthermore, the SMO classifier gives the highest percentage of accuracy and the least amount of fault prediction fault on (HDD Mono) in terms of 80/20 (87.72%), 70/30 (89.41%), and 5 folds cross-validation (88.38%), and (HDD-Multi) in terms of 80/20 (93.64%), 70/30 (90.91%), and 5 folds cross-validation (88.20%).

In the primary data results, the RF classifier has the highest percentage of accuracy and less fault prediction 80/20 (97.14%), 70/30 (96.19%), and 5 folds cross-validation (95.85%), but the algorithm complexity (0.17 seconds) is poor. SMO has the second highest accuracy and less fault prediction in terms of 80/20 (95.71%), 70/30 (95.71%), and 5 folds cross-validation (95.71%), but the algorithm complexity is good (0.3 seconds). The difference in accuracy and fault prediction between RF and SMO is only (.13%), and the difference in time complexity is only (.13%). (14 seconds).

### Achievements of the objectives

Research objectives have been achieved successfully as shown in Table 4 with the help and literature review to achieve high accuracy and less fault prediction errors in CC.

**Table 4:** Research objectives achievement.

S.No	Objective	Input & Output	Achievements
1	Identify the best ML-based classifiers to improve accuracy and failure prediction.	An extensive literature review has been conducted. Keywords to search articles include “Popular ML techniques to achieve high accuracy and less fault prediction errors”, “ML classifiers”, “ML approach for fault classification and prediction in CC” etc, which were used to search in Google Scholar, Web of Science, and Science Direct databases.	Identified best ML classifiers to achieve high accuracy and less fault prediction errors.
2	To propose an ML- model to address low accuracy, and high failure prediction errors.	First, identify the best ML classifier that provides high accuracy and less fault prediction errors and then modify it for optimum results.	Identified the SMO classifier that provided high accuracy and less fault prediction errors and then modified it to achieve optimum results.

### Contribution towards cloud computing

The SMO classifier has been modified. Our proposed approach improves accuracy and gets fewer fault prediction errors for users in cloud computing environments. It was not an easy task to achieve high accuracy and less fault prediction to reliability to the existing approach. Almost all tuning parameters C parameter, random seed, kernel exponent, and lower order values have been adjusted to get better results in terms of accuracy, mean square error, and better fitness.

This research work has proven that the ML-based approach can be greatly contributed to cloud computing to achieve high accuracy and fewer fault prediction errors for cloud computing users.

### Limitations

1. Antarex secondary data collection is possible, but more computational resources are required because this is an HPC fault dataset, however, we can download this dataset through the ZONODO website.
2. The Weibull distribution was not provided to generate a fault dataset for primary data generation.
3. An effort was made to achieve the primary dataset using the Weibull distribution.

### Future directions

1. Using the Weibull distribution approach, a graphical user interface can be created to generate the primary dataset in CloudSim.
2. Tuning parameters can be automatically adjusted using code, but keep in mind that to find the best tuning parameter value, the code must not become stuck.
3. Random Forest can be implemented to achieve high accuracy and less fault prediction errors, but more work on the algorithm’s complexity is required. Comparative analysis can also be performed with this proposed work.
4. Deep Learning algorithms can also be used to achieve high accuracy while predicting less faults. The sample size should be increased. The larger the sample size, the more accurate and reliable the results. When the dataset is large, DL techniques outperform ML techniques.

## Supporting information

S1 Dataset (RAR)

S1 Appendix (DOCX)
