# Supplementary material for: Improved accuracy and less fault prediction errors via modified sequential minimal optimization algorithm
Source: PLoS One. 2023 Apr 13;18(4):e0284209. doi: 10.1371/journal.pone.0284209 (PMC10101450; doi:10.1371/journal.pone.0284209)
Supplement: S1 Appendix — (DOCX) [file pone.0284209.s002.docx]

**Appendix**

**Table 5. A summary of the literature review.**

| **Ref** | **Author**  **Name** | **Year** | **Benefits** | **Drawbacks** |
| --- | --- | --- | --- | --- |
| [6] | Shahid et al. | 2020 | They identify the need for FT efficiency metrics in algorithms in this article, which is one of the main concerns in cloud environments. | They do not provide quality service in terms of reliability. |
| [7] | Shahid et al. | 2021 | The newly developed Resilient Methods aim to reduce the amount of time it takes for a device to detect a malfunction. Machine Learning and Artificial Intelligence were critical in mapping the recovery period to a task to be configured in the context of Resilient Methods. | Lack of defects to manage in conventional approaches. |
| [8] | Ahmed et al. | 2020 | This paper examines the cloud computing system, its technologies, and the best technologies to use with it based on a variety of factors and criteria such as procedure cost, speed, cons, and pros. | Lack of increase in the execution time of data nodes. |
| [9] | Kamiri & Mariga | 2021 | The primary goals of this paper were to investigate current machine learning research methods, emerging themes, and the implications of those themes in machine learning research. | They do not offer content analysis for machine learning applications such as supervised learning, text analytics, classification, and prediction. |
| [10] | Sarker | 2021 | He provides a comprehensive overview of machine learning algorithms, which can be used to improve an application's intelligence and capabilities. | Lack of analysis on machine learning algorithms. |
| [11] | Butt et al. | 2020 | They present an analysis of CC security threats, issues, and solutions that used one or more ML algorithms in this review paper. | Lack of a proposed solution to achieve reliability based on VM failure. |
| [12] | Sun et al. | 2019 | In this article, they use ML algorithms to improve accuracy. | Lack of challenges and open problems in ML optimization methods. |
| [13] | Kochhar et al. | 2017 | The proactive fault tolerance technique is used in this article, and they propose using the NB classifier to classify the nodes. | Lack of use of other classification algorithms to improve accuracy and less fault prediction. |
| [14] | Chang & Lin | 2022 | In this article, they present the implementation of LibSVM & discuss all issues. | Lack of ensuring good system reliability. |
| [15] | Mohamad | 2016 | This study is using MLR model to determine fault prediction. | Lack of use of other classification algorithms to determine fault prediction. |
| [16] | C.R. LI & J. GUO | 2015 | The authors of this paper proposed an improved version of SVM that can avoid falling into endless loops. | Inability to determine the optimal parameter in an n-way that can speed up training. |
| [17] | Sun et al. | 2020 | This paper attempts to compare different types of classification algorithms precisely and widely used based on some basic concepts. Although it is obvious that a complete and comprehensive review and survey of all the supervised learning classification algorithms is impossible. | Lack of performance evaluation of supervised learning classification algorithms. |

**Table 6. Short overview of secondary dataset (CPU-mem mono) block-I [22].**

| **Timestamp** | **Type** | **Args** | **Seqnum** | **Duration** | **Cores** | **Error** | **Isfault** |
| --- | --- | --- | --- | --- | --- | --- | --- |
| 1525853147 | command_session_s | None | None | None | None | None | None |
| 1525853168 | status_start | sudo/home/anetti/finj/faultlib/cpufreq 1007 | 0 | 1007 | 0 | None | TRUE |
| 1525854175 | status_end | sudo/home/anetti/finj/faultlib/cpufreq 1007 | 0 | 1007 | 0 | None | TRUE |
| 1525854475 | status_start | sudo/home/anetti/finj/faultlib/pagefail 260 | 1 | 260 | 0 | None | TRUE |
| 1525854735 | status_end | sudo/home/anetti/finj/faultlib/pagefail 260 | 1 | 260 | 0 | None | TRUE |
| 1525854900 | status_start | /home/anetti/benchmarks/dgemm/dgemm.out 2000 | 2 | 1710 | 0 | None | FALSE |
| 1525855464 | status_start | /home/anetti/finj/faultlib/leak 256 l | 3 | 256 | 0 | None | TRUE |
| 1525855525 | status_restart | /home/anetti/benchmarks/dgemm/dgemm.out 2000 | 2 | 1710 | 0 | None | FALSE |
| 1525855720 | status_end | /home/anetti/finj/faultlib/leak 256 l | 3 | 256 | 0 | None | TRUE |
| 1525856003 | status_start | /home/anetti/finj/faultlib/ddot 264 | 4 | 264 | 0 | None | TRUE |
| 1525856267 | status_end | /home/anetti/finj/faultlib/ddot 264 | 4 | 264 | 0 | None | TRUE |
| 1525856280 | status_restart | /home/anetti/benchmarks/dgemm/dgemm.out 2000 | 2 | 1710 | 0 | None | FALSE |
| 1525856595 | status_start | /home/anetti/finj/faultlib/memeater 228 | 5 | 228 | 0 | None | TRUE |
| 1525856610 | status_end | /home/anetti/benchmarks/dgemm/dgemm.out 2000 | 2 | 1710 | 0 | None | FALSE |
| 1525856823 | status_end | /home/anetti/finj/faultlib/memeater 228 | 5 | 228 | 0 | None | TRUE |

**Table 7. Short overview of secondary dataset (CPU-mem multi) block-II [22].**

| **Timestamp** | **Type** | **Args** | **Seqnum** | **Duration** | **Cores** | **Error** | **Isfault** |
| --- | --- | --- | --- | --- | --- | --- | --- |
| 1529849044 | command_session_s | None | None | None | None | None | None |
| 1529849065 | status_start | /home/anetti/finj/faultlib/ddot 290 | 0 | 290 | 7 | None | TRUE |
| 1529849355 | status_end | /home/anetti/finj/faultlib/ddot 290 | 0 | 290 | 7 | None | TRUE |
| 1529849640 | status_start | /home/anetti/finj/faultlib/leak 205 l | 1 | 205 | 7 | None | TRUE |
| 1529849845 | status_end | /home/anetti/finj/faultlib/leak 205 l | 1 | 205 | 7 | None | TRUE |
| 1529850189 | status_start | sudo/home/anetti/finj/faultlib/cpufreq 230 | 2 | 230 | 4 | None | TRUE |
| 1529850419 | status_end | sudo/home/anetti/finj/faultlib/cpufreq 230 | 2 | 230 | 4 | None | TRUE |
| 1529850684 | status_start | /home/anetti/benchmarks/dgemm/dgemm.sh | 3 | 1697 | 4 |  | FALSE |
| 1529850866 | status_start | /home/anetti/finj/faultlib/leak 251 l | 4 | 251 | 2 | None | TRUE |
| 1529851104 | status_restart | /home/anetti/benchmarks/dgemm/dgemm.sh | 3 | 1697 | 4 |  | FALSE |
| 1529851117 | status_end | /home/anetti/finj/faultlib/leak 251 l | 4 | 251 | 2 | None | TRUE |
| 1529851415 | status_start | /home/anetti/finj/faultlib/ddot 279 l | 5 | 279 | 7 | None | TRUE |
| 1529851587 | status_restart | /home/anetti/benchmarks/dgemm/dgemm.sh | 3 | 1697 | 4 |  | FALSE |
| 1529851694 | status_end | /home/anetti/finj/faultlib/ddot 279 l | 5 | 279 | 7 | None | TRUE |
| 1529852052 | status_start | /home/anetti/finj/faultlib/ddot 223 | 6 | 223 | 3 | None | TRUE |
| 1529852053 | status_restart | /home/anetti/benchmarks/dgemm/dgemm.sh | 3 | 1697 | 4 |  | FALSE |
| 1529852275 | status_end | /home/anetti/finj/faultlib/ddot 223 | 6 | 223 | 3 | None | TRUE |

**Table 8. Short overview of secondary dataset (HDD mono) block-III [22].**

| **Timestamp** | **Type** | **Args** | **Seqnum** | **Duration** | **Cores** | **Error** | **Isfault** |
| --- | --- | --- | --- | --- | --- | --- | --- |
| 1527171947 | command_session_s | None | None | None | None | None | None |
| 1527171968 | status_start | sudo/home/anetti/finj/faultlib/ioerr 233 l | 0 | 233 | 0 | None | TRUE |
| 1527172201 | status_end | sudo/home/anetti/finj/faultlib/ioerr 233 l | 0 | 233 | 0 | None | TRUE |
| 1527172587 | status_start | /home/anetti/finj/faultlib/copy 325 l | 1 | 325 | 0 | None | TRUE |
| 1527172913 | status_end | /home/anetti/finj/faultlib/copy 325 l | 1 | 325 | 0 | None | TRUE |
| 1527173724 | status_start | /home/anetti/benchmarks/bonnie++/bonnie++.sh | 2 | 1723 | 0 | None | FALSE |
| 1527174407 | status_restart | /home/anetti/benchmarks/bonnie++/bonnie++.sh | 2 | 1723 | 0 | None | FALSE |
| 1527174998 | status_start | sudo/home/anetti/finj/faultlib/ioerr 223 l | 3 | 223 | 0 | None | TRUE |
| 1527175067 | status_restart | /home/anetti/benchmarks/bonnie++/bonnie++.sh | 2 | 1723 | 0 | 1 | FALSE |
| 1527175221 | status_end | Sudo/home/anetti/finj/faultlib/ioerr 223 l | 3 | 223 | 0 | None | TRUE |
| 1527175449 | status_end | /home/anetti/benchmarks/bonnie++/bonnie++.sh | 2 | 1723 | 0 | None | FALSE |
| 1527175556 | status_start | /home/anetti/finj/faultlib/copy 469 l | 4 | 469 | 0 | None | TRUE |
| 1527176026 | status_end | /home/anetti/finj/faultlib/copy 469 l | 4 | 469 | 0 | None | TRUE |

**Table 9. Short overview of secondary dataset (HDD multi) block-IV [22].**

| **Timestamp** | **Type** | **Args** | **Seqnum** | **Duration** | **Cores** | **Error** | **Isfault** |
| --- | --- | --- | --- | --- | --- | --- | --- |
| 1528278158 | command_session_s | None | None | None | None | None | None |
| 1528278179 | status_start | /home/anetti/finj/faultlib/copy 239 l | 0 | 239 | TRUE | 2 | None |
| 1528278418 | status_end | /home/anetti/finj/faultlib/copy 239 l | 0 | 239 | TRUE | 2 | None |
| 1528278800 | status_start | /home/anetti/finj/faultlib/copy 234 l | 1 | 234 | TRUE | 6 | None |
| 1528279034 | status_end | /home/anetti/finj/faultlib/copy 234 l | 1 | 234 | TRUE | 6 | None |
| 1528279414 | status_start | /home/anetti/finj/faultlib/copy 232 l | 2 | 232 | TRUE | 5 | None |
| 1528279646 | status_end | /home/anetti/finj/faultlib/copy 232 l | 2 | 232 | TRUE | 5 | None |
| 1528279892 | status_start | /home/anetti/benchmarks/bonnie++/bonnie++.sh | 3 | 1741 | FALSE | 0 | None |
| 1528280183 | status_start | sudo/home/anetti/finj/faultlib/ioerr 320 l | 4 | 320 | TRUE | 0 | None |
| 1528280503 | status_end | sudo/home/anetti/finj/faultlib/ioerr 320 l | 4 | 320 | TRUE | 0 | None |
| 1528280565 | status_restart | /home/anetti/benchmarks/bonnie++/bonnie++.sh | 3 | 1741 | FALSE | 0 | None |
| 1528280727 | status_start | sudo/home/anetti/finj/faultlib/ioerr 237 l | 5 | 237 | TRUE | 5 | None |

**Table 10. Short overview of secondary dataset (CPU-mem mono) block-I after data pre-processing.**

| **Timestamp** | **Type** | **Args** | **Seqnum** | **Duration** | **Cores** | **Error** | **Isfault** |
| --- | --- | --- | --- | --- | --- | --- | --- |
| 1525853168 | status_start | sudo/home/anetti/finj/faultlib/cpufreq 1007 | 0 | 1007 | 0 | None | TRUE |
| 1525854175 | status_end | sudo/home/anetti/finj/faultlib/pagefail 260 | 1 | 260 | 0 | None | TRUE |
| 1525854475 | status_start | /home/anetti/benchmarks/dgemm/dgemm.out 2000 | 2 | 1710 | 0 | None | TRUE |
| 1525854735 | status_end | /home/anetti/finj/faultlib/leak 256 l | 3 | 256 | 0 | None | TRUE |
| 1525854900 | status_start | /home/anetti/finj/faultlib/ddot 264 | 4 | 264 | 0 | None | FALSE |
| 1525855464 | status_start | /home/anetti/finj/faultlib/memeater 228 | 5 | 228 | 0 | None | TRUE |
| 1525855525 | status_restart | /home/anetti/benchmarks/hpcc/hpcc | 6 | 1734 | 0 | None | FALSE |
| 1525855720 | status_end | /home/anetti/finj/faultlib/memeater 244 l | 7 | 244 | 0 | None | TRUE |
| 1525856003 | status_start | /home/anetti/finj/faultlib/memeater 259 | 8 | 259 | 0 | None | TRUE |
| 1525856267 | status_end | sudo/home/anetti/finj/faultlib/pagefail 238 l | 9 | 238 | 0 | None | TRUE |
| 1525856280 | status_restart | /home/anetti/finj/faultlib/dial 240 | 10 | 240 | 0 | None | FALSE |
| 1525856595 | status_start | /home/anetti/finj/faultlib/memeater 303 l | 11 | 303 | 0 | None | TRUE |
| 1525856610 | status_end | /home/anetti/benchmarks/stream/stream.out | 12 | 1733 | 0 | None | FALSE |

**Table 11. Short overview of secondary dataset (CPU-mem multi) block-II after data pre-processing.**

| **Timestamp** | **Type** | **Args** | **Seqnum** | **Duration** | **Cores** | **Error** | **Isfault** |
| --- | --- | --- | --- | --- | --- | --- | --- |
| 1529849065 | status_start | /home/anetti/finj/faultlib/ddot 290 | 0 | 290 | 7 | None | TRUE |
| 1529849355 | status_end | /home/anetti/finj/faultlib/leak 205 l | 1 | 205 | 7 | None | TRUE |
| 1529849640 | status_start | sudo/home/anetti/finj/faultlib/cpufreq 230 | 2 | 230 | 7 | None | TRUE |
| 1529849845 | status_end | /home/anetti/finj/faultlib/leak 251 l | 4 | 251 | 7 | None | TRUE |
| 1529850189 | status_start | /home/anetti/finj/faultlib/ddot 279 l | 5 | 279 | 4 | None | TRUE |
| 1529850419 | status_end | /home/anetti/finj/faultlib/ddot 223 | 6 | 223 | 4 | None | TRUE |
| 1529850866 | status_start | /home/anetti/finj/faultlib/ddot 294 l | 8 | 294 | 2 | None | TRUE |
| 1529851117 | status_end | /home/anetti/finj/faultlib/memeater 234 l | 9 | 234 | 2 | None | TRUE |
| 1529851415 | status_start | sudo/home/anetti/finj/faultlib/pagefail 449 | 10 | 449 | 7 | None | TRUE |
| 1529851694 | status_end | /home/anetti/finj/faultlib/ddot 272 l | 11 | 272 | 7 | None | TRUE |
| 1529852052 | status_start | sudo/home/anetti/finj/faultlib/pagefail 317 | 13 | 317 | 3 | None | TRUE |
| 1529852275 | status_end | /home/anetti/finj/faultlib/ddot 239 l | 14 | 239 | 3 | None | TRUE |
| 1529853113 | status_start | /home/anetti/finj/faultlib/dial 233 l | 15 | 233 | 6 | None | TRUE |
| 1529853407 | status_end | /home/anetti/finj/faultlib/leak 294 | 16 | 294 | 6 | None | TRUE |
| 1529853664 | status_start | /home/anetti/finj/faultlib/memeater 229 l | 18 | 229 | 5 | None | TRUE |
| 1529853898 | status_end | /home/anetti/finj/faultlib/leak 217 | 19 | 217 | 5 | None | TRUE |

**Table 12. Short overview of secondary dataset (HDD mono) block-III after data pre-processing.**

| **Timestamp** | **Type** | **Args** | **Seqnum** | **Duration** | **Cores** | **Error** | **Isfault** |
| --- | --- | --- | --- | --- | --- | --- | --- |
| 1527171968 | status_start | sudo/home/anetti/finj/faultlib/ioerr 233 l | 0 | 233 | 0 | None | TRUE |
| 1527172201 | status_end | /home/anetti/finj/faultlib/copy 325 l | 1 | 325 | 0 | None | TRUE |
| 1527172587 | status_start | /home/anetti/benchmarks/bonnie++/bonnie++.sh | 2 | 1723 | 0 | None | TRUE |
| 1527172913 | status_end | sudo/home/anetti/finj/faultlib/ioerr 223 l | 3 | 223 | 0 | None | TRUE |
| 1527173724 | status_start | /home/anetti/finj/faultlib/copy 469 l | 4 | 469 | 0 | None | FALSE |
| 1527174407 | status_restart | /home/anetti/benchmarks/iozone/iozone.sh | 5 | 1715 | 0 | None | FALSE |
| 1527174998 | status_start | sudo/home/anetti/finj/faultlib/ioerr 289 l | 6 | 289 | 0 | None | TRUE |
| 1527175067 | status_restart | /home/anetti/finj/faultlib/copy 258 | 7 | 258 | 0 | 1 | FALSE |
| 1527175221 | status_end | sudo/home/anetti/finj/faultlib/ioerr 249 | 8 | 249 | 0 | None | TRUE |
| 1527175449 | status_end | /home/anetti/finj/faultlib/copy 228 | 9 | 228 | 0 | None | FALSE |
| 1527175556 | status_start | /home/anetti/benchmarks/bonnie++/bonnie++.sh | 10 | 1734 | 0 | None | TRUE |
| 1527176026 | status_end | sudo/home/anetti/finj/faultlib/ioerr 236 l | 11 | 236 | 0 | None | TRUE |
| 1527176032 | status_start | /home/anetti/finj/faultlib/copy 247 l | 12 | 247 | 0 | None | FALSE |
| 1527176215 | status_start | /home/anetti/finj/faultlib/copy 204 | 13 | 204 | 0 | None | TRUE |
| 1527176236 | status_restart | /home/anetti/finj/faultlib/copy 228 | 14 | 228 | 0 | None | FALSE |

**Table 13. Short overview of secondary dataset (HDD multi) block-IV after data pre-processing.**

| **Timestamp** | **Type** | **Args** | **Seqnum** | **Duration** | **Cores** | **Error** | **Isfault** |
| --- | --- | --- | --- | --- | --- | --- | --- |
| 1528278179 | status_start | /home/anetti/finj/faultlib/copy 239 l | 0 | 239 | 2 | None | TRUE |
| 1528278418 | status_end | /home/anetti/finj/faultlib/copy 234 l | 1 | 234 | 2 | None | TRUE |
| 1528278800 | status_start | /home/anetti/finj/faultlib/copy 232 l | 2 | 232 | 6 | None | TRUE |
| 1528279034 | status_end | /home/anetti/benchmarks/bonnie++/bonnie++.sh | 3 | 1741 | 6 | None | TRUE |
| 1528279414 | status_start | sudo/home/anetti/finj/faultlib/ioerr 320 l | 4 | 320 | 5 | None | TRUE |
| 1528279646 | status_end | sudo/home/anetti/finj/faultlib/ioerr 237 l | 5 | 237 | 5 | None | TRUE |
| 1528279892 | status_start | sudo/home/anetti/finj/faultlib/ioerr 253 l | 6 | 253 | 0 | None | FALSE |
| 1528280183 | status_start | /home/anetti/benchmarks/bonnie++/bonnie++.sh | 7 | 1735 | 0 | None | TRUE |
| 1528280503 | status_end | sudo/home/anetti/finj/faultlib/ioerr 253 | 8 | 253 | 0 | None | TRUE |
| 1528280565 | status_restart | /home/anetti/finj/faultlib/copy 221 l | 9 | 221 | 0 | None | FALSE |
| 1528280727 | status_start | sudo/home/anetti/finj/faultlib/ioerr 228 l | 10 | 228 | 5 | None | TRUE |
| 1528280964 | status_end | /home/anetti/benchmarks/iozone/iozone.sh | 11 | 1736 | 5 | None | TRUE |
| 1528281238 | status_restart | /home/anetti/finj/faultlib/copy 246 l | 12 | 246 | 0 | None | FALSE |
| 1528281634 | status_end | /home/anetti/finj/faultlib/copy 230 l | 13 | 230 | 0 | None | FALSE |
| 1528281668 | status_start | /home/anetti/finj/faultlib/copy 237 l | 14 | 237 | 1 | None | TRUE |
| 1528281921 | status_end | /home/anetti/finj/faultlib/copy 277 l | 15 | 277 | 1 | None | TRUE |
